# Challenges in Developing a Validated Biomarker for Angiogenesis Inhibitors: The Motesanib Experience

**DOI:** 10.1371/journal.pone.0108048

**Published:** 2014-10-14

**Authors:** Michael B. Bass, Bin Yao, Yong-Jiang Hei, Yining Ye, Gerard J. Davis, Michael T. Davis, Barbara A. Kaesdorf, Sabrina S. Chan, Scott D. Patterson

**Affiliations:** 1 Molecular Sciences and Computational Biology, Amgen Inc., Thousand Oaks, CA, United States of America; 2 Global Biostatistical Science, Amgen Inc., Thousand Oaks, CA, United States of America; 3 Oncology Development, Amgen Inc., Thousand Oaks, CA, United States of America; 4 Global Biostatistical Science, Amgen Inc. South San Francisco, San Francisco, CA, United States of America; 5 Diagnostics Division Research and Development, Abbott Laboratories, Abbott Park, IL, United States of America; 6 Medical Sciences, Amgen Inc., Thousand Oaks, CA, United States of America; University Clinic of Navarra, Spain

## Abstract

**Purpose:**

We sought to develop placental growth factor as a predictive pharmacodynamic biomarker for motesanib efficacy as first-line therapy in patients with advanced nonsquamous non–small-cell lung cancer.

**Experimental Design:**

Placental growth factor was evaluated at baseline and study week 4 (after 3 weeks treatment) in an exploratory analysis of data from a randomized phase 2 study of motesanib 125 mg once daily plus carboplatin/paclitaxel and in a prespecified analysis of data from a randomized, double-blind phase 3 study of motesanib 125 mg once daily plus carboplatin/paclitaxel vs placebo plus carboplatin/paclitaxel (MONET1). Associations between fold-change from baseline in placental growth factor and overall survival were evaluated using Cox proportional hazards models.

**Results:**

In the phase 2 study, serum placental growth factor increased from baseline a mean 2.8-fold at study week 4. Patients with ≥2.2-fold change from baseline in placental growth factor (n = 18) had significantly longer overall survival than those with <2.2-fold change (n = 19; 22.9 vs 7.9 months; hazard ratio, 0.30; 95% CI, 0.12–0.74; *P* = 0.009). Consequently, placental growth factor was investigated as a pharmacodynamic biomarker in the phase 3 MONET1 study. There was no association between log-transformed placental growth factor fold-change from baseline to week 4 (continuous variable) and overall survival (hazard ratio, 0.98; 95% CI, 0.79–1.22; *P* = 0.868). MONET1 did not meet its primary endpoint of overall survival. Likewise, median overall survival was similar among patients with ≥2.0-fold change in placental growth factor (n = 229) compared with <2.0-fold change (n = 127; 14.8 vs 13.8 months; hazard ratio, 0.88; 95% CI, 0.67–1.15, *P* = 0.340).

**Conclusions:**

Our results illustrate the challenges of successfully translating phase 2 biomarker results into phase 3 studies.

**Trial Registration:**

ClinicalTrials.gov NCT00460317, NCT00369070

## Introduction

The important role of angiogenesis in tumor development, growth, and metastasis is well established [1]. Angiogenesis inhibitors, including the anti−vascular endothelial growth factor (VEGF) monoclonal antibody bevacizumab; the recombinant anti-VEGF fusion protein aflibercept; and the receptor tyrosine kinase inhibitors sorafenib, sunitinib, axitinib, vandetanib, and pazopanib, have been shown to improve outcomes for patients with certain cancer types, either as monotherapy or combined with chemotherapy [2]–[4]. However, only a fraction of treated patients typically derive clinical benefit. Predictive biomarkers identifying patients most likely to respond would allow for a more targeted approach to treatment and, therefore, would be of significant clinical value. To date, no validated biomarker has been identified for any angiogenesis inhibitor despite extensive investigation. Lambrechts et al [5] recently described efforts to identify predictive biomarkers for bevacizumab: although potential markers have been identified in certain tumor types, as yet none have proven robust. A recent prospective study found an association between low VEGF-A levels and both progression-free survival and overall survival in patients with nonsquamous NSCLC [6]. However, because the study did not include a control arm it was not possible to differentiate between prognostic and predictive value of the biomarker.

Motesanib is a potent small-molecule inhibitor of VEGF receptors (VEGFR) 1, 2, and 3; platelet-derived growth factor receptor; and Kit [7], with demonstrated antitumor activity as monotherapy [8]–[10] and in combination with chemotherapy [11]. In the motesanib first-in-human study, analysis of potential biomarker candidates showed a strong pharmacodynamic response of placental growth factor (PLGF) and further suggested that increased levels of PLGF from baseline were associated with increased motesanib exposure and possibly correlated with tumor shrinkage [8]. PLGF is a VEGF-A homolog and a VEGFR1 ligand that is up-regulated during hypoxia [12], [13], and may be involved in pathologic angiogenesis, possibly by increasing the responsiveness of endothelial cells to VEGF-A [14]–[17]. The increase in PLGF following motesanib treatment possibly represents a compensatory upregulation in response to VEGF pathway blockade. Subsequent phase 2 studies with motesanib showed a consistent association between increased levels from baseline in PLGF and outcomes across different tumor types, including thyroid cancer, breast cancer, and non–small-cell lung cancer (NSCLC) [8], [18]–[20]. Furthermore, other inhibitors of the VEGF pathway have been known to induce pharmacodynamic changes in PLGF [21]–[26], which, in some cases, have been associated with outcomes including objective response and OS. Taken together, the data suggested that PLGF may serve as a biomarker for the biologic effect of VEGF receptor inhibitors, and as such, it may be a potential biomarker identifying a population most likely to benefit from continued treatment with these agents.

 The PLGF data collected in motesanib phase 2 studies [8], [18]–[20] formed a strong body of evidence that supported further prospective testing of PLGF as a potential biomarker in the large international phase 3 Motesanib NSCLC Efficacy and Tolerability (MONET1) study of motesanib plus carboplatin/paclitaxel versus placebo plus carboplatin/paclitaxel in patients with nonsquamous NSCLC. However, the study did not meet its primary endpoint, and PLGF analysis with a validated assay developed specifically as a companion diagnostic test did not reveal an association between change from baseline in PLGF and OS [27]. To date, MONET1 remains the only large, prospective study of a biomarker candidate for an angiogenesis inhibitor. Considering the body of evidence for PLGF as a biomarker for motesanib and the rigorous analysis of data that formed the basis of the PLGF hypothesis for MONET1, the study’s negative biomarker results demonstrate the challenges in the development of a valid predictive biomarker. Here we describe the processes we undertook in an effort to develop PLGF as a pharmacodynamic biomarker for motesanib using an ongoing phase 3 study of motesanib in patients with NSCLC and supporting data from the preceding phase 2 study of motesanib in NSCLC. We hope that our experiences will help others who intend to develop predictive biomarkers based on early biomarker data by highlighting the challenges of applying late-emerging biomarker data to ongoing clinical trials.

## Methods

The protocol for this trial and supporting CONSORT checklist are available as supporting information; see Checklist S1 and Protocol S1.

### Clinical Studies

PLGF was evaluated as a pharmacodynamic biomarker for motesanib in patients with advanced nonsquamous NSCLC in an exploratory analysis of a randomized phase 2 study (ClinicalTrials.gov, NCT00369070; http://clinicaltrials.gov/ct2/show/NCT00369070) [11], and as a secondary endpoint in a prespecified analysis of a randomized placebo-controlled phase 3 study (MONET1; ClinicalTrials.gov, NCT00460317; http://clinicaltrials.gov/ct2/show/NCT00460317) [27].

The phase 2 study [11] enrolled patients (≥18 years) with unresectable stage IIIB nonsquamous NSCLC with pericardial or pleural effusion or stage IV/recurrent nonsquamous NSCLC (histologically or cytologically confirmed), measurable disease per Response Evaluation Criteria in Solid Tumors (RECIST) version 1.0 [28], Eastern Cooperative Oncology Group (ECOG) performance status of ≤1, and life expectancy ≥3 months. Patients received up to six 3-week cycles of paclitaxel (200 mg/m^2^) plus carboplatin (target area under the curve of 6 mg/mL•min) administered in 3-week cycles and were randomized 1∶1∶1 to also receive motesanib 125 mg once daily (QD) continuously (Arm A), motesanib 75 mg twice daily 5 days on/2 days off (Arm B), or bevacizumab 15 mg/kg once every 3 weeks (Arm C). Treatment with motesanib/bevacizumab could continue for up to 3 years or until radiographic disease progression or unacceptable toxicity occurred. Administration of each study drug could be delayed or doses reduced according to protocol-specific rules if patients experienced toxicity. The primary endpoint was the objective response rate (ORR) per RECIST [28] as evaluated by an independent central review (Radpharm Inc., Princeton, NJ); PFS and OS were secondary endpoints.

The phase 3 MONET1 study initially enrolled patients with NSCLC of all histologies but was amended to enroll only patients with nonsquamous histology owing to unacceptable toxicity in patients with squamous histology who received motesanib [27]. Following this amendment, patients (≥18 years) were eligible if they had histologically confirmed unresectable stage IIIB nonsquamous NSCLC with pericardial/pleural effusion or stage IV recurrent nonsquamous NSCLC, measurable or nonmeasurable disease per RECIST version 1.0 [28], ECOG performance status ≤1, and life expectancy ≥3 months. Patients received up to six 3-week cycles of carboplatin/paclitaxel (at the same dose and schedule as in the phase 2 study) and were randomized to also receive motesanib 125 mg QD (Arm A) or placebo (Arm B). Randomization was stratified by disease stage (IIIB vs IV/recurrent), weight loss (≥5%) in the previous 6 months, sex, and prior adjuvant chemotherapy. Treatment continued until disease progression or unacceptable toxicity occurred. OS (the primary endpoint), PFS, and ORR (secondary endpoints) were evaluated for all nonsquamous patients and for the subset of patients with adenocarcinoma. The study was planned to enroll 1060 patients with nonsquamous histology and was estimated to have 80% power to detect a hazard ratio (HR) of 0.80 for OS with an α = 0.03 (two-sided) and 80% power to detect an HR of 0.77 for OS with an α = 0.02 in the adenocarcinoma subset.

### Ethics Statement

Study procedures were conducted in accordance with the principles expressed in the Declaration of Helsinki and approved by an independent ethics committee/institutional review board at each study site. All patients provided written informed consent.

### Biomarker and Pharmacokinetic Samples

In the phase 2 study, serum and EDTA plasma samples were collected from patients before administration of therapy at study weeks 1 (baseline), 4, 7, 13, 19, 25, 31, 37, 43, 49, and 55, and 30 days after study completion (safety follow-up visit). Plasma samples for motesanib pharmacokinetic analyses were collected at 1 and 24 hours postdose on day 1 of cycle 1. In the phase 3 study, serum and EDTA plasma samples were obtained before administration of treatment on the first day of cycles 1, 2, and 3 and at 6-week intervals thereafter. In both studies, samples were stored at −80°C, and serum was prepared as previously described [20], [29].

### Placental Growth Factor Assays

In the phase 2 study, PLGF in the serum was measured using a Meso-Scale Discovery (MSD, Gaithersburg, MD) electrochemiluminescent multiplexed sandwich immunoassay [29]. This assay has been previously described [20], and its performance was characterized according to previously reported procedures [29]. The lower limit of quantitation (LLOQ) was 10 pg/mL which was sufficient to measure PLGF in all study patients. The upper limit of quantitation (ULOQ) was 4500 pg/mL.

In the phase 3 study, plasma PLGF concentrations were analyzed using an ARCHITECT investigational use immunoassay (Abbott Diagnostics, Abbott Park, IL). Fifty microliters of sample were incubated for 18 minutes with a PLGF-specific mouse monoclonal capture antibody linked to paramagnetic microparticle beads. Following a wash step, an acridinium-conjugated mouse monoclonal anti-PLGF F(ab′)_2_ detection fragment was added, and incubation continued for an additional 4 minutes. After a second wash step, pretrigger and trigger buffers were added and the chemiluminescent signal was measured using ARCHITECT *i* System optics. The assay had a LLOQ of 3.4 pg/mL, a linear range from 4 to 1500 pg/mL without dilution and to 3000 pg/mL with automated 2-fold dilution. Total precision (%CV) for assay controls and serum panels ranged from 2 to 6.5% across two instruments and two reagent lots across 20 days. For accuracy, there is no certified standard commercially available for the PLGF analyte. A commercially available antigen was used to develop an Amino Acid Analyses (AAA)–based extinction coefficient for an A280-independent method to assign a concentration value to PLGF internal standards. The ARCHITECT PLGF immunoassay was calibrated with calibrators matched to AAA-assigned internal standards.

### Statistical Analysis of Biomarker Data

In the phase 2 study, all patients who received ≥1 dose of treatment and who had blood samples at baseline and after 3 weeks of treatment (ie, study week 4) were included in the biomarker analysis set. In the phase 3 study, patients were additionally required to have received motesanib the day before the week 4 PLGF sample collection. Patients with samples that did not meet predefined assay acceptance criteria or that had values below the LLOQ were excluded. Patients who did not provide either a baseline or a postbaseline blood sample were included in the undetermined biomarker group. Before all statistical analyses, biomarker values were log-transformed to normalize data. In both studies, PFS time was defined as the number of days from randomization until disease progression (per RECIST) [28] or death. OS time was defined as the time from randomization to death.

In the analysis of the data from the phase 2 study, both the fold-changes from baseline in PLGF and a binary version of the fold-change at each cut point were explored as covariates in Cox proportional hazard models of OS and PFS [30]. The binary cut points for fold-change in PLGF were identified based on maximally selected rank statistics [31]; a fold-change value of 2.2-fold was the optimal threshold in the phase 2 study. HRs with 95% confidence intervals (CIs) were obtained from the Cox models. *P* values were not corrected for multiple comparisons. Receiver operating characteristic (ROC) curves were also calculated [32], [33].

In the phase 3 study, paired *t* tests were used to evaluate whether PLGF was significantly increased over time for patients in the motesanib group compared with patients in the placebo group, adjusting for baseline PLGF. *P* values were adjusted to control the false discovery rate at 5% [34]. For patients treated with motesanib, associations between OS and fold-change from baseline in PLGF were evaluated using a log-rank test, as comparisons were within the treated subjects rather than against placebo. Conditional on a statistically significant association of OS with log-transformed PLGF fold-change, OS was to be compared between patients with a ≥2.0-fold change in PLGF and patients with a <2.0-fold change in PLGF using a log-rank test at α = 0.03 for patients with nonsquamous and α = 0.02 for patients with adenocarcinoma histology. The 2.0-fold threshold was determined as outlined in the Results. Additionally, the HR (and corresponding CI) associated with each unit increase of log-transformed PLGF was calculated.

## Results

### Biomarker Results From the Phase 2 Study

**Figure 1:**
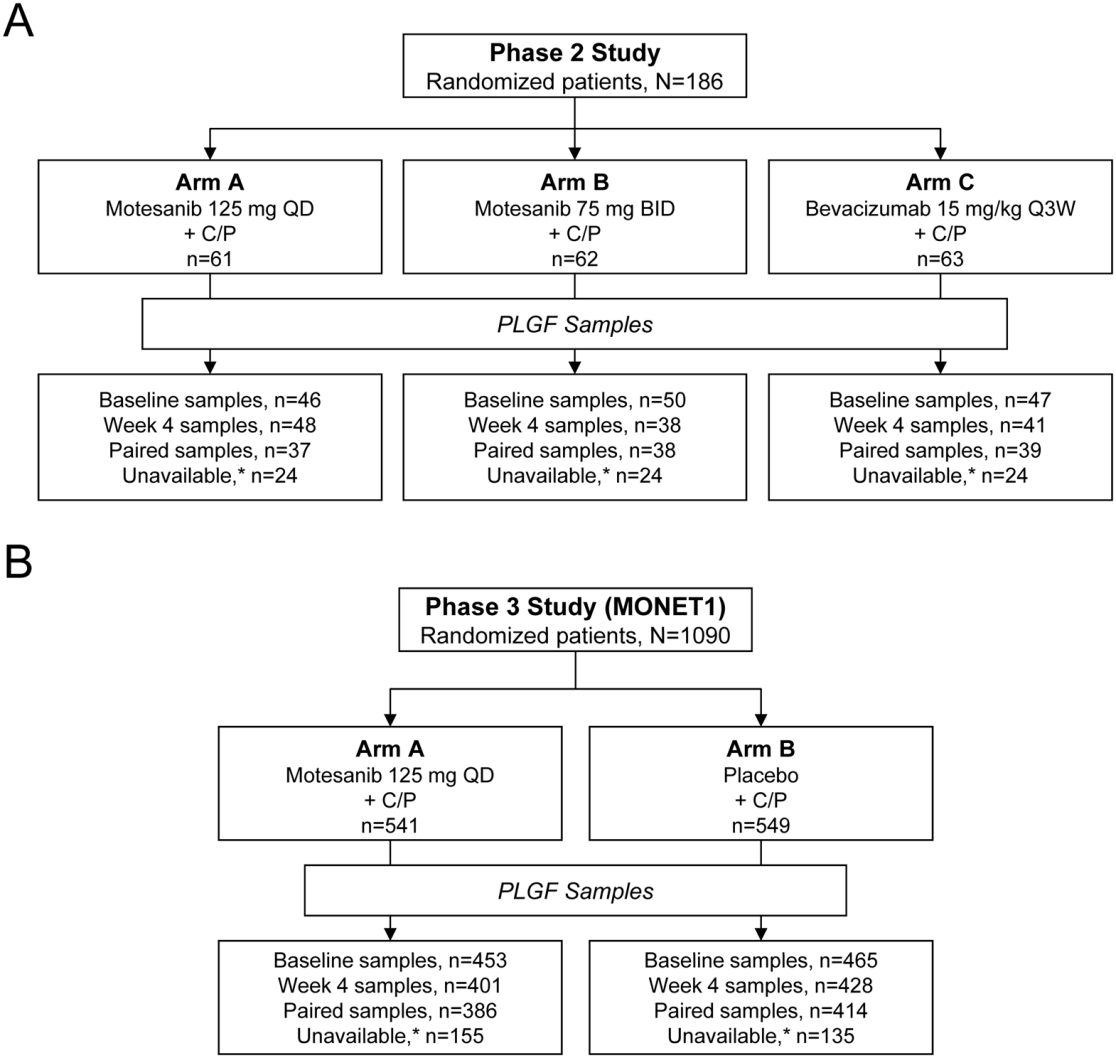
CONSORT diagram. Disposition of patients and availability of PLGF samples from patients enrolled in (**A**) the phase 2 study and (**B**) the phase 3 MOtesanib NSCLC Efficacy and Tolerability (MONET1) study of motesanib in non–small-cell lung cancer are shown. BID = twice daily; C/P = carboplatin and paclitaxel; QD = once daily; Q3W = every 3 weeks. *Patients without paired PLGF samples.

As reported previously, the phase 2 study enrolled 186 patients with advanced nonsquamous NSCLC; 181 received treatment with either motesanib or bevacizumab (Arm A, n = 59; Arm B, n = 62; Arm C, n = 60; **Figure 1A**) [11]. In the primary analysis the Kaplan-Meier estimates of median (95% CI) PFS in Arms A, B, and C were 7.7 (5.6–9.3) months, 5.8 (4.3–7.8) months, and 8.3 (6.8–10.2) months, respectively. Estimated median (95% CI) OS in Arms A, B, and C were 14.0 (7.7–18.6) months, 12.8 (7.9–17.0) months, and 14.0 (9.3–16.8) months, respectively [11].

**Figure 2:**
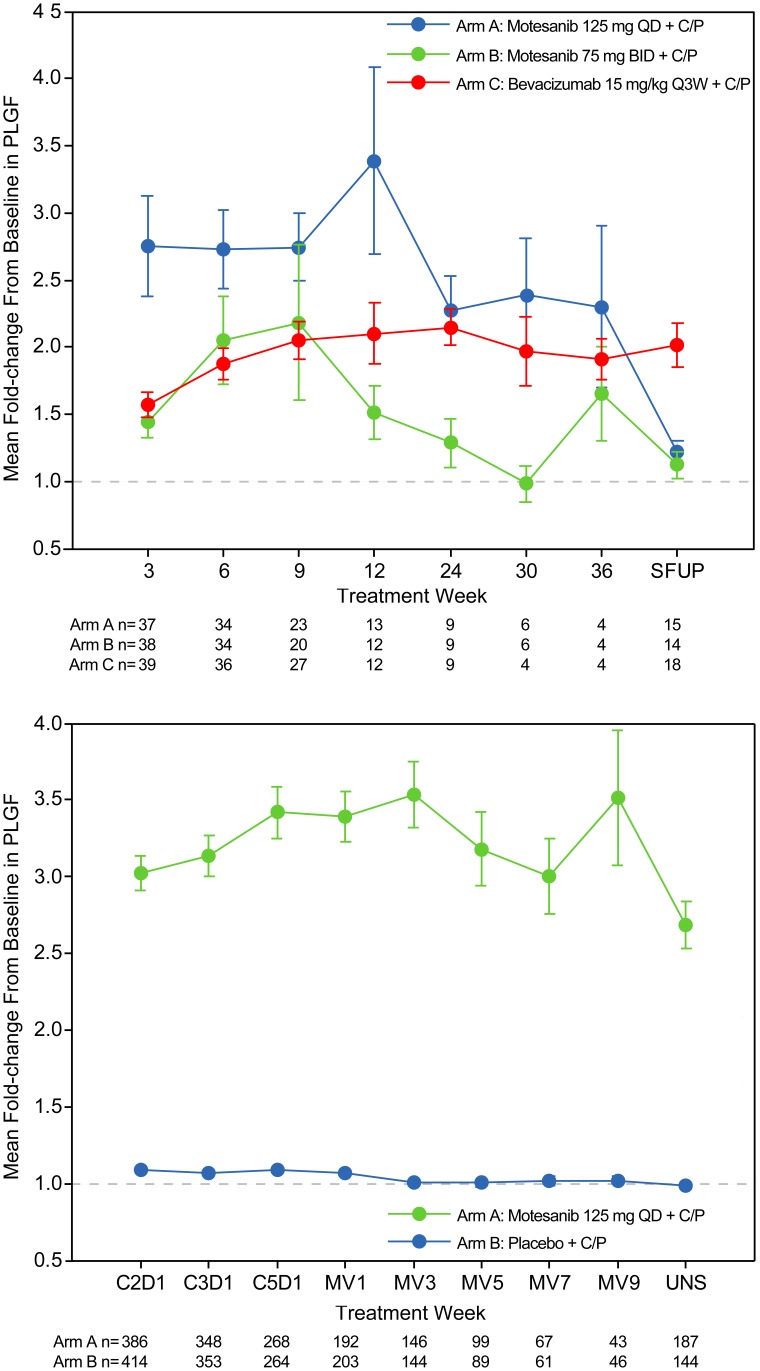
Pharmacodynamic changes in placental growth factor (PLGF). Mean fold-change (±SE) from baseline in serum PLGF over time in response to treatment with motesanib during the phase 2 study (**A**) and the phase 3 Motesanib NSCLC Efficacy and Tolerability study (**B**). BID = twice daily; C = cycle; D = day; MV = maintenance visit; Q3W = once every 3 weeks; QD = once daily; SFUP = safety follow-up; UNS = unscheduled.

**Figure 3:**
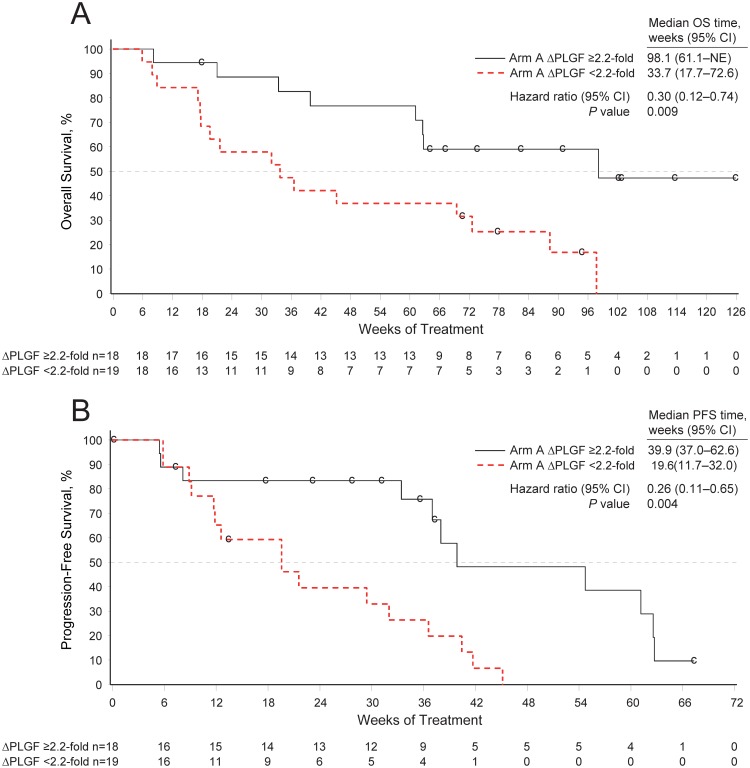
Association of fold-change in placental growth factor (PLGF) and outcomes in the phase 2 study. Overall survival (OS) (**A**) and progression-free survival (PFS) (**B**) in treatment Arm A among patients with a ≥2.2-fold (black line) or <2.2-fold (red line) fold-change from baseline in PLGF after 3 weeks of treatment with motesanib 125 mg QD. HR = hazard ratio; NE = not estimable; QD = once daily.

One hundred fourteen patients had evaluable PLGF samples at baseline and at week 4 (Arm A, n = 37; Arm B, n = 38; Arm C, n = 39; **Figure 1A**). In Arm A, mean serum PLGF levels increased 2.8-fold from baseline (*P*<0.001) at week 4 (ie, after one treatment cycle) and remained elevated >2-fold throughout the study (**Figure 2A**). The magnitude of change in PLGF at this time point was associated with OS and PFS. The PLGF threshold providing the best separation of survival times, based on the method of maximally selected rank statistics [31], was a 2.2-fold change. Among the 37 evaluable patients, 18 (49%) had a ≥2.2-fold increase in PLGF. These patients also had longer OS (22.9 vs 7.9 months; HR, 0.30; 95% CI, 0.12–0.74; *P* = 0.009) and PFS (9.2 vs 4.5 months; HR, 0.26; 95% CI, 0.11–0.65; *P* = 0.004) compared with those who had a <2.2-fold increase (n = 19) (**Figure 3**). It is important to note that patients with a <2.2-fold and a ≥2.2-fold increase in PLGF were comparable with respect to demographic and baseline disease characteristics (**Table 1**). Similarly, when fold-change in PLGF was modeled as a continuous variable in Cox proportional hazards models, it was associated with OS (*P* = 0.018) and PFS (*P* = 0.003).

**Table 1:** Patient Demographics and Baseline Disease Characteristics, Motesanib Exposure (Phase 2 Study).

	Arm A Motesanib 125 mg QD n = 61		
Characteristic, n (%)	Δ PLGF ≥2.2-Fold Change n = 18	Δ PLGF <2.2-Fold Change n = 19	Undetermined n = 24
**Sex**			
Women	10 (56)	6 (32)	12 (50)
Men	8 (44)	13 (68)	12 (50)
**Race**			
White	13 (72)	11 (58)	15 (63)
Black	3 (17)	1 (5)	2 (8)
Asian	2 (11)	5 (26)	3 (13)
Other*	0 (0)	2 (11)	4 (17)
**Age group, y**			
<65	14 (78)	9 (47)	15 (63)
≥65	4 (22)	19 (53)	9 (38)
**Histology**			
Adenocarcinoma	13 (72)	13 (68)	21 (88)
Large cell carcinoma	2 (11)	2 (11)	1 (4)
Bronchoalveolar carcinoma	1 (6)	0 (0)	0 (0)
Undifferentiated	0 (0)	2 (11)	1 (4)
Other	2 (11)	2 (11)	0 (0)
Missing	0 (0)	0 (0)	1 (4)
**ECOG performance status**			
0	9 (50)	8 (42)	9 (38)
1	9 (50)	10 (53)	15 (63)
2	0 (0)	1 (5)	0 (0)
**Disease stage**			
Stage IIIB	1 (6)	4 (21)	5 (21)
Stage IV	17 (94)	15 (79)	19 (79)
**Number of sites of disease** †			
1	1 (6)	1 (5)	5 (21)
2	6 (33)	7 (37)	7 (29)
3	7 (39)	8 (42)	5 (21)
≥4	4 (22)	3 (16)	7 (29)
Number of prior therapies‡			
0	16 (89)	16 (84)	22 (92)
1	1 (6)	3 (16)	2 (8)
≥2	1 (6)	0 (0)	0 (0)
**Tobacco use (cigarettes)**			
Never	4 (22)	5 (26)	5 (21)
Former	9 (50)	10 (53)	14 (58)
Current	5 (28)	4 (21)	4 (17)
Missing	0 (0)	0 (0)	1 (4)
**Motesanib Exposure** §			
*C_1h_, ng/mL*			
n	18	17	17
Median	231.5	246	269
(IQR)	(58.9–361)	(141.0–427.0)	(55.3–326.0)
*C_24h_, ng/mL*			
n	16	16	16
Median	18	14.4	34.7
(IQR)	(12.4–41.3)	(6.8–19.6)	(23.8–46.9)

C_1h_ = plasma motesanib concentration at 1 hour; C_24h_ = plasma motesanib concentration at 24 hours; ECOG = Eastern Cooperative Oncology Group; IQR = interquartile range; PLGF = placental growth factor; QD = once daily.

*Includes Hispanic, American Indian or Alaska Native, and other.

† As assessed by investigator.

‡ Includes all cancer therapies before study enrollment.

§ Only 22 of 24 patients in the “undetermined” group received motesanib and were therefore evaluated for motesanib exposure. Motesanib concentrations in plasma were determined using a validated liquid chromatography/tandem mass spectrometry method (Cedra Corp., Austin, TX).

We performed a series of additional evaluations to further test the robustness of PLGF as a biomarker. Twenty-four patients had undetermined biomarker status because of missing samples at baseline and/or after 3 weeks of treatment. Demographics and baseline characteristics of these patients were similar to those for patients with known biomarker status, although more patients in the undetermined group had adenocarcinoma histology (88%) than in the ≥2.2-fold change (72%) and <2.2-fold change (68%) groups (**Table 1**). OS and PFS for patients with undetermined biomarker status was within the range of data reported for the two PLGF fold-change groups (**Figure S1** in **Appendix S1**). Notably, the association between fold-change in PLGF and PFS and OS remained when patients with undetermined biomarker status were included in either the ≥2.2-fold PLGF change group or the <2.2-fold change group (*P* = 0.038 and 0.013, respectively). Moreover, the association between fold-change in PLGF as a continuous covariate and OS remained when Cox proportional hazard models were adjusted for the baseline covariates of age, ethnicity, sex, disease stage, ECOG status, histology (adenocarcinoma vs other), and weight loss before therapy. Finally, change in PLGF from baseline at week 4 in Arm A was associated with outcomes independently of exposure to motesanib. The area under the ROC curve for fold-change in PLGF was 0.726 (n = 37). When predicting 1-year survival based on a PLGF increase of 2.2-fold, the sensitivity was 74% and the specificity was 66% (**Figure S2** in **Appendix S1**). In contrast to the area under the ROC curve for fold-change in PLGF, the areas under the ROC curves for the 1-hour and 24-hour exposure to motesanib were 0.525 and 0.426, respectively (**Figure S3** in **Appendix S1**). The association between fold-change in PLGF and OS remained when motesanib 1-hour and 24-hour exposures were included in the Cox proportional hazards models. In any combination of PLGF, 1-hour, or 24-hour exposure terms, exposure never had an association with survival and PLGF was always had the strongest association (**Table S1** in **Appendix S1**). Finally, permutation testing was performed in which the outcome was reordered with respect to fold-change in PLGF. Using a total of 10,000 permutations of the data set, the original finding remained (empirical *P* value of 0.045).

### Biomarker Hypothesis (MONET1 Study)

As described in the Introduction, a robust body of evidence, including results from the phase 2 study of motesanib in NSCLC, suggested that change in PLGF from baseline occurring early in treatment was associated with response to motesanib. Consequently, a prospective hypothesis was formed that those patients who achieved a ≥2-fold increase in PLGF from baseline after the first 3 weeks of motesanib treatment would have a survival advantage over those patients whose response was below this cut-off. After gaining agreement from US regulatory authorities, the protocol of the MONET1 phase 3 study of motesanib plus carboplatin/paclitaxel was amended to prospectively evaluate PLGF as a biomarker in patients with nonsquamous histology. Specifically, the primary objective of the biomarker analysis was to assess whether improved OS was associated with increased log-transformed PLGF fold-change at week 4. Conditional on a significant association between OS and the PLGF fold change, PLGF was to be evaluated as a binary variable with a cut-off point of a 2.0-fold change in PLGF from baseline. The 2.0-fold threshold was determined based on the analysis of the phase 2 study biomarker data [11], which used a cutpoint of 2.2-fold. The threshold value of 2.0-fold was chosen because it is an even number that was within the range identified in the phase 2 study. It should be noted that there were no patient values between 2.0-fold and 2.2-fold (the midpoint between the two existing patient values).

### Companion Diagnostic Test

Consistent with most biomarker studies, our phase 1 and 2 studies used a laboratory-developed MSD assay to evaluate PLGF. The ARCHITECT platform was used for evaluation of PLGF in the phase 3 study because of its potential as a companion diagnostic. The ARCHITECT assay was highly concordant with the MSD assay (**Table S2** in **Appendix S1**). When serum samples from the phase 2 study were retested on the ARCHITECT system, the Lin concordance correlation [35] (rho) was 0.807 and the Pearson correlation (r) was 0.835. To ensure the broadest applicability of the assay, circulating PLGF was evaluated in plasma samples in the phase 3 study. Concordance between the MSD and ARCHITECT assays was confirmed in both serum and plasma (rho and r; **Figure S4** in **Appendix S1**). When phase 2 plasma samples were retested using the MSD assay, the association between fold-change in PLGF and outcomes was reaffirmed. Similarly, when samples from the phase 2 study were retested on the ARCHITECT platform, the correlation to PFS and OS was reaffirmed (**Figure S4** in **Appendix S1**).

### Biomarker Results From the Phase 3 Study

The primary analysis of the MONET1 study did not show a statistically significant improvement in OS (the primary endpoint) with motesanib plus carboplatin/paclitaxel treatment, compared with placebo, in either nonsquamous patients (median OS, 13.0 vs 11.0 months; HR, 0.90; 95% CI, 0.78–1.04; *P* = 0.14) or the adenocarcinoma subset (13.5 vs 11.0 months, respectively; HR, 0.88; 95% CI, 0.75–1.03; *P* = 0.11) [27]. Therefore, all evaluations of PLGF as a biomarker are descriptive.

Among the 918 patients with PLGF samples at baseline, median (interquartile range) baseline PLGF was 24.5 pg/mL (20.80–29.30 pg/mL; **Table S3** in **Appendix S1**). Of nonsquamous patients who received motesanib plus carboplatin/paclitaxel in Arm A (n = 541) and placebo plus carboplatin/paclitaxel in Arm B (n = 549), 356 (66%) and 400 (73%) patients in Arms A and B, respectively, had paired biomarker samples at baseline and after one cycle of treatment at week 4 (**Figure 1B** **;** **Table 2**). No notable differences in patient demographics and baseline characteristics were observed across the various analysis subgroups (patients with high and low change in PLGF, patients with undetermined PLGF status, and patients evaluable for PLGF) and between treatment arms. Consistent with the phase 2 study, there was a pharmacodynamic increase in PLGF among patients in Arm A that was maintained throughout the study (median 2.30-fold at week 4; **Figure 2B**). In Arm B, PLGF remained unchanged from baseline over the course of the study; 95% CIs did not extend above 1.25-fold at any timepoint.

**Table 2:** Patient Demographics and Baseline Disease Characteristics, Motesanib Exposure (Phase 3 Study).

	Arm A Motesanib 125 mg QD n = 541			Arm B Placebo n = 549	
Characteristic, n (%)	Δ PLGF ≥2-Fold Change n = 229	Δ PLGF <2-Fold Change n = 127	Undetermined n = 185	Evaluable for PLGF n = 400	Undetermined n = 149
**Sex** *					
Women	99 (43)	39 (31)	69 (37)	160 (40)	53 (36)
Men	130 (57)	88 (69)	116 (63)	240 (60)	96 (64)
**Race**					
White	142 (62)	82 (65)	138 (75)	253 (63)	100 (67)
Black	0	2 (2)	7 (4)	5 (1)	2 (1)
Asian†	67 (16)	40 (31)	32 (17)	116 (29)	32 (21)
Other	20 (9)	3 (2)	8 (4)	26 (7)	15 (10)
**Age group, y**					
<65	139 (61)	95 (75)	118 (64)	273 (68)	95 (64)
≥65	90 (39)	32 (25)	67 (36)	127 (32)	54 (36)
**Histology**					
Adenocarcinoma	198 (86)	104 (82)	146 (79)	320 (80)	122 (82)
Bronchoalveolar carcinoma	8 (3)	3 (2)	3 (2)	15 (4)	0
Large cell carcinoma	8 (3)	3 (2)	15 (8)	29 (7)	6 (4)
Undifferentiated	3 (1)	5 (4)	13 (7)	13 (3)	8 (5)
Other	12 (5)	12 (9)	8 (4)	23 (6)	13 (9)
**ECOG performance status**					
0	78 (34)	45 (35)	65 (35)	157 (39)	50 (34)
≥1	151 (66)	82 (65)	120 (65)	243 (61)	99 (66)
**Disease stage at study entry** *					
Stage IIIB with pericardial/pleural effusion	26 (11)	21 (17)	26 (14)	52 (13)	27 (18)
Stage IV/recurrent	203 (89)	106 (83)	159 (86)	348 (87)	122 (82)
**Number of sites of disease**					
0	0 (0)	0 (0)	2 (1)	0 (0)	1 (<1)
1	35 (15)	13 (10)	26 (14)	53 (13)	27 (18)
2	95 (41)	39 (31)	71 (38)	144 (36)	55 (37)
≥3	99 (43)	75 (59)	86 (46)	203 (51)	66 (44)
**Prior adjuvant chemotherapy** *	8 (3)	0 (0)	3 (2)	10 (3)	2 (1)
**Weight loss <5% in previous** **6 months** *	56 (24)	35 (28)	53 (29)	105 (26)	37 (25)
**Tobacco use (cigarettes)**					
Never	66 (29)	39 (31)	49 (26)	111 (28)	39 (26)
Former	113 (49)	46 (36)	86 (46)	184 (46)	67 (45)
Current	50 (22)	42 (33)	50 (27)	104 (26)	42 (28)
Missing	0 (0)	0 (0)	0 (0)	1 (<1)	1 (<1)
**Motesanib Exposure**					
Days on which motesanibwas administered					
Median	142	132	46	134.5	63
Range	(21–1094)	(25–974)	(1–686)	(22–954)	(18–155)

ECOG = Eastern Cooperative Oncology Group PLGF = placental growth factor; QD = once daily.

*Randomization stratification factors.

† Includes Japanese patients.

**Figure 4:**
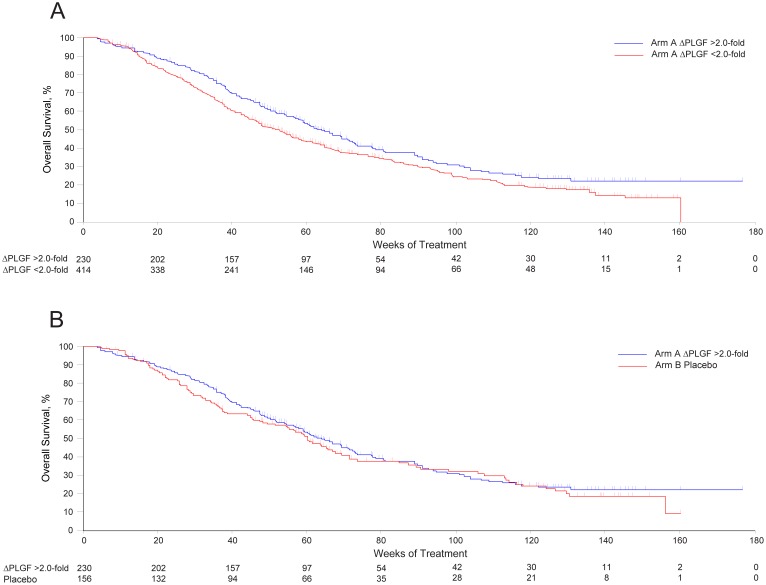
Association of fold-change in placental growth factor (PLGF) and outcomes in the MONET1 study. Overall survival in Arm A among patients with a ≥2.0-fold (blue line) or <2.0-fold (red line) change from baseline in PLGF is shown in (**A**). Overall survival in Arm A among patients with a ≥2.0-fold change from baseline in PLGF (blue line) compared with placebo (red line; patients with undetermined PLGF status were excluded) is shown in (**B**).

When evaluated as a continuous variable, there was no association between the log-transformed PLGF fold-change from baseline to week 4 and OS (unadjusted Cox model, HR, 0.98; 95% CI, 0.79–1.22; *P* = 0.868) (**Figure 4A**). Median (95% CI) OS was 14.8 (13.1–16.8) months among the 229 patients with a ≥2.0-fold change in PLGF from baseline and 13.8 (11.4–15.9) months among the 127 patients with a <2.0-fold change in PLGF. There was no association between a ≥2.0-fold change in PLGF from baseline at week 4 and OS (HR, 0.88; 95% CI, 0.67–1.15, *P* = 0.340). Similarly, there was no association between a ≥2.0-fold change in PLGF and PFS (HR, 0.89; 95% CI, 0.70–1.12, *P* = 0.32). In subsequent exploratory analyses, associations with PFS using a 2.2-fold threshold (as identified in the phase 2 study) or a 2.3-fold threshold (representing the median fold-change in the phase 3 study) were also not significant.

Interestingly, baseline PLGF was identified as a prognostic factor for OS in another exploratory analysis using the median split of these values. Median OS was 9.4 months among patients with PLGF >24.5 pg/mL at baseline compared with 14.9 months for patients below this cutoff (HR, 1.53; 95% CI 1.31–1.78; *P*<0.0001). A model evaluating baseline PLGF as a continuous variable also found an association with OS (*P*<0.0001). Associations between baseline PLGF and OS were also investigated in the phase 2 study but none were identified.

When patients in Arm A with a ≥2.0-fold change in PLGF were compared with the entire placebo group (Arm B) in exploratory analyses, there was a difference in OS (14.8 vs 12.1 months; HR, 0.79; 95% CI, 0.65–0.96, *P* = 0.017; **Figure 4B**) and PFS (6.8 vs 5.5 months; HR, 0.70; 95% CI, 0.58–0.84; *P* = 0.0001). Furthermore, OS was not different when the population was dichotomized by median PLGF change (HR, 0.90; 95% CI, 0.70–1.16; *P* = 0.42).

## Discussion

In early phase studies evaluating motesanib in patients with solid tumors, increases in circulating PLGF were observed shortly after initiation of motesanib treatment [8], [18]–[20]. Similar pharmacodynamic changes in circulating PLGF have been described in response to treatment with sunitinib, sorafenib, bevacizumab, pazopanib, and cediranib [21]–[26]. Because PLGF signalling plays a role in pathologic angiogenesis [14]–[17], it could be hypothesized that the pharmacodynamic changes might be a marker for the antitumor activity of these agents. Consistent with this hypothesis, results from several motesanib studies suggested that change from baseline in PLGF may be associated with tumor regression and PFS [8], [18]–[20]. As described in this report, the pharmacodynamic PLGF response to motesanib treatment was confirmed in a phase 2 study in patients with NSCLC. Taken together, these data indicated that the PLGF response was not tumor type-specific and that associations with outcomes, although not always significant, could be seen across tumor types.

Although these results provided promising evidence in support of PLGF as a potential pharmacodynamic biomarker for motesanib treatment (particularly in patients with NSCLC), they had certain limitations. The data were derived from small phase 1 and 2 studies that were not prospectively designed for biomarker discovery, the biomarker ascertainment rate was not always high, and analyses were not adjusted for multiple testing. Consequently, we employed several different approaches to assess the robustness of results from the phase 2 study in NSCLC. The association between fold-change in PLGF and OS remained when Cox proportional hazards models were adjusted for baseline covariates and when motesanib exposure was included in the model. Additionally, to account for the possibility that the approximately one third of patients who were unevaluable for PLGF influenced the outcome, we performed an analysis in which these patients were included in either the ≥2.2-fold or <2.2-fold–change groups; in both instances, the association with OS remained. Finally, in permutation simulations in which the outcome was shuffled with respect to PLGF, the association with OS remained (*P* = 0.045). Of interest, although PLGF was confirmed to be a pharmacodynamic biomarker, drug exposure did not predict response (**Figure S3** in **Appendix S1**), suggesting that PLGF response to motesanib was a better indicator of clinical response than exposure to the drug.

Although the data from the phase 2 NSCLC study were intriguing, it was clear that the hypothesis of PLGF being a pharmacodynamic biomarker that predicts outcome in patients with NSCLC who receive motesanib would require further testing. Ideally, this would occur in a larger prospective validation study specifically designed to validate a predictive pharmacodynamic response biomarker [36]. Such a study might have employed a run-in design, in which motesanib was administered to all patients then, after stratification by change in PLGF, patients would be randomized to either continue or discontinue treatment with motesanib. However, when the PLGF biomarker hypothesis for motesanib emerged, the large international, double-blind, randomized, placebo-controlled MONET1 study of motesanib plus carboplatin/paclitaxel in patients with NSCLC was already actively enrolling [27]. Although MONET1 was initially designed only to evaluate the efficacy of motesanib compared with placebo, it provided an important opportunity, given the PLGF data available at the time, to add biomarker testing as a secondary endpoint to a large phase 3 study, even though MONET1 was nearing the end of its enrollment period at that time. The approach required numerous challenges to be overcome in a short period of time. The protocol amendement had to consider where the biomarker endpoint(s) should be placed in the context of the other endpoints and how statistical analysis of these endpoints will be approached. Some studies split alpha between the secondary endpoints, giving equal weight to each [37]. Other studies (including MONET1) use a sequential testing of the secondary endpoints. In the case of MONET1, the study protocol amendment was discussed with both drug and diagnostics divisons of the US Food and Drug Administration (FDA). Its approval through the FDA’s Special Protocol Assessment; agreement with the FDA on assay performance and testing; and development of an in vitro diagnostic (IVD) assay that was reliable, robust, and easily implemented in a clinical setting [38], all had to take place before the event trigger for the primary analysis. Development of the IVD assay also necessitated validation of the phase 2 PLGF results using the follow-on ARCHITECT assay system to ensure that the association between fold-change in PLGF and OS remained when using the new companion diagnostic.

Ultimately, the MONET1 study did not confirm change in PLGF as a prognostic biomarker for motesanib. It is reasonable to speculate that, despite analysis of all known covariates, the dataset from the phase 2 study had unidentified confounders that unknowingly introduced bias towards a positive identification of change in PLGF as a potential predictive pharmacodynamic effect, a possibility which has been identified as a potential problem in small biomarker-derived subgroups [39]. Other challenges may have contributed to the outcome. While the sample ascertainment rate for paired biomarker samples that were used to compute PLGF change was high (69%), it may not be representative of the entire study. Initial studies had shown a pharmacodynamic effect as early as 24 hours after the first dose of motesanib [20], but the earliest evaluation of PLGF in the phase 3 NSCLC studies was after 3 weeks of treatment (a time point selected prior to formation of the biomarker hypothesis, at the time of study design). Although this time point was the same as that used in the phase 2 study, the possibility that earlier timepoints might be significantly associated with survival could not be evaluated. Furthermore, subgroups defined according to postrandomization characteristics are more prone to biases compared with those based on baseline characteristics. Identification of high PLGF responders before randomization was not possible and, as observed in both the phase 2 and 3 studies, placebo patients did not have PLGF increases beyond random temporal variation. Finally, although extensive efforts were made to ensure concordance between the MSD and ARCHITECT assays, it is possible that use of a different PLGF assay may have contributed to the outcome. Each of these obstacles highlights the difficulty in assessing the predictive utility of biomarkers.

Despite the outcome of the MONET1 biomarker analysis, we believe that adding biomarker testing as a secondary endpoint to an ongoing phase 3 study represented a timely and scientifically robust approach that also illustrates the challenges involved in biomarker development in an oncology setting. In particular, evidence for a biomarker typically does not appear early in the drug development process (except when the biomarker is very closely linked to the therapeutic target); instead, it usually emerges during phase 2 evaluation [36] and often after a phase 3 study has been initiated. In our case, the PLGF biomarker hypothesis was developed in early-phase testing, with analysis of the phase 2 data occurring while a phase 3 study was ongoing. Consequently, the PLGF hypothesis was added to the phase 3 study following interactions with the FDA. While the option of assessing PLGF as a predictive pharmacodynamic biomarker for motesanib in a larger, independent phase 2 study first represented a scientifically ideal approach, it would have resulted in significant delays in evaluating the hypothesis with no guarantee of a positive outcome. Potentially, a confirmatory prospective run-in design trial (as described above) may have been considered had the PLGF biomarker hypothesis been confirmed in MONET1.

It has been suggested that less than 1% of published cancer biomarkers are routinely used in the clinical setting [40]. Factors identified as contributing to failure to translate biomarkers into the clinic include lack of clinical practicality of the biomarker, hidden biases within the data, an inadequate assay, inappropriate statistical techniques, and lack of biologic plausibility for the biomarker [40]. Although we were not successful in developing a predictive biomarker for motesanib in NSCLC our approach adequately addressed these factors. Biomarker identification was included in early-phase studies, we developed adequate statistical techniques, a robust diagnostic test to evaluate PLGF, and engaged early with the US FDA to gain support for our protocol amendment. However, using a pharmacodynamic biomarker as a predictor of efficacy remains an unproven approach. Such biomarkers have typically only been used to identify toxicity issues [41] and there is no precedent that could have guided the development of the biomarker portion of our study.

Our experience illustrates several significant challenges to develop predictive pharmacodynamic biomarkers in oncology. Ideal approaches calling for specific study designs and/or sequences of events should be applied wherever possible in an effort to maximize the chances of success; however, they seldom reflect the unpredictable scenarios that may unfold during drug development. Furthermore, a methodical, no-risk approach must be balanced against economic factors and the desire to rapidly identify patient populations that may benefit the most from a potential new treatment. Despite these challenges, it remains important to develop biomarker hypotheses and to subject them to objective evaluation in clinical studies. Development of predictive pharmacodynamic biomarkers remains an opportunity to markedly improve outcomes for patients.

## Supporting Information

Appendix S1 **Supporting files.** Contains Figures S1, S2, S3, and S4, and Tables S1, S2, and S3.
(PDF)

Appendix S2 **Contains patient-level data from the phase 2 study.**
(PDF)

Appendix S3 **Contains patient-level data from the phase 3 study.**
(PDF)

Checklist S1 **CONSORT checklist.**
(PDF)

Protocol S1 **Phase 3 study protocol.**
(PDF)
