# Correction: Challenges in Developing a Validated Biomarker for Angiogenesis Inhibitors: The Motesanib Experience

**DOI:** 10.1371/journal.pone.0121162

**Published:** 2015-03-26

**Authors:** The PLOS ONE Staff

The Kaplan-Meier curves for Fig. 4A and Fig. 4B are incorrectly switched. The curve that appears as Fig. 4A should be Fig. 4B, and the curve that appears as Fig. 4B should be Fig. 4A. Please see the corrected Fig. 4 here.

**Fig 4:**
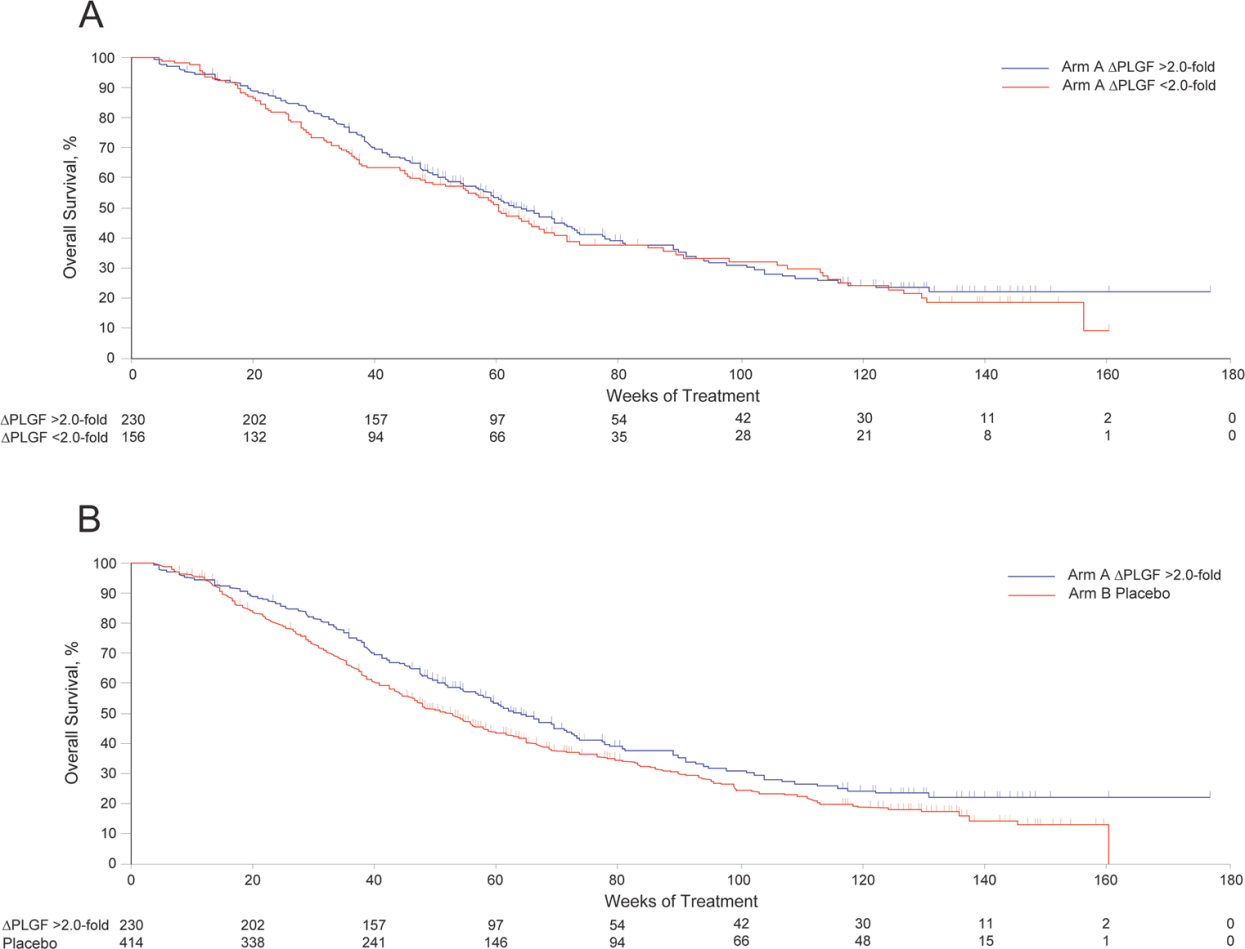
Association of fold-change in placental growth factor (PLGF) and outcomes in the MONET1 study. Overall survival in Arm A among patients with a ≥2.0-fold (blue line) or <2.0-fold (red line) change from baseline in PLGF is shown in (**A**). Overall survival in Arm A among patients with a ≥2.0-fold change from baseline in PLGF (blue line) compared with placebo (red line; patients with undetermined PLGF status were excluded) is shown in (**B**).
