# Supplementary material for: Challenges in Developing a Validated Biomarker for Angiogenesis Inhibitors: The Motesanib Experience
Source: PLoS One. 2014 Oct 14;9(10):e108048. doi: 10.1371/journal.pone.0108048 (PMC4196848; doi:10.1371/journal.pone.0108048)
Supplement: Appendix S1 — Supporting files. Contains Figures S1, S2, S3, and S4, and Tables S1, S2, and S3. (PDF) [file pone.0108048.s001.pdf]

APPENDIX S1

Challenges in Developing a Validated Biomarker for Angiogenesis Inhibitors: The Motesanib Experience

Michael B. Bass, Bin Yao, Yong-Jiang Hei, Yining Ye, Gerard J. Davis,
Michael T. Davis, Barbara A. Kaesdorf, Sabrina S. Chan,
Scott D. Patterson

Figure S1. Association of fold-change in placental growth factor (PLGF) and outcomes: patients with undetermined biomarker status..

(**A**) Overall survival (OS) and (**B**) progression-free survival (PFS) in treatment Arm A of the phase 2 non-small-cell lung cancer study among patients with a ≥2.2-fold or <2.2-fold increase from baseline in PLGF, and patients with undetermined PLGF status (fold-change in PLGF could not be calculated because of missing samples at baseline and/or after 3 weeks of treatment). NE=not estimable.

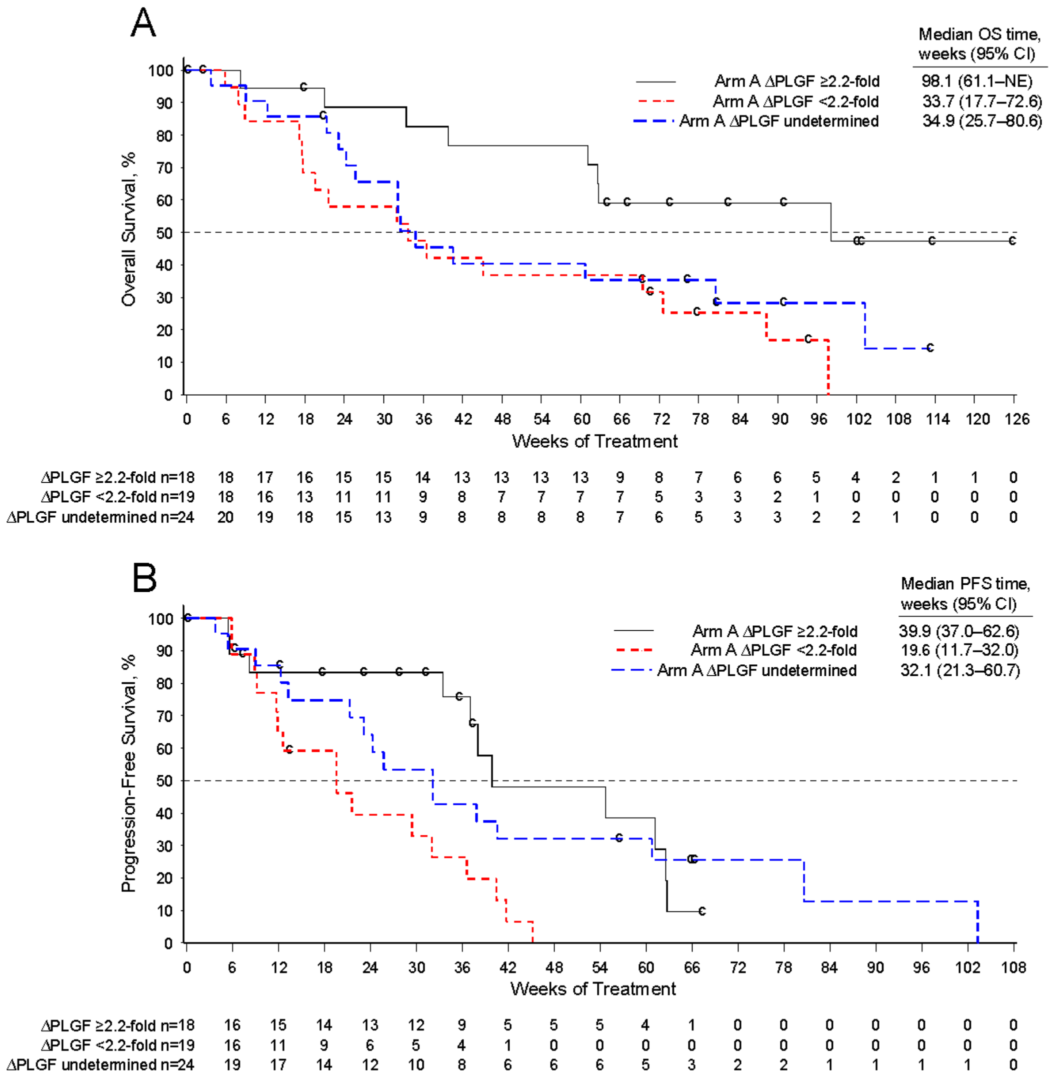

Figure S2. Sensitivity and specificity of fold-change in placental growth factor (PLGF) as a biomarker.

Receiver operating characteristic curves of fold-change in PLGF after 3 weeks treatment predicting survival beyond 1 year. AUC=area under the curve.

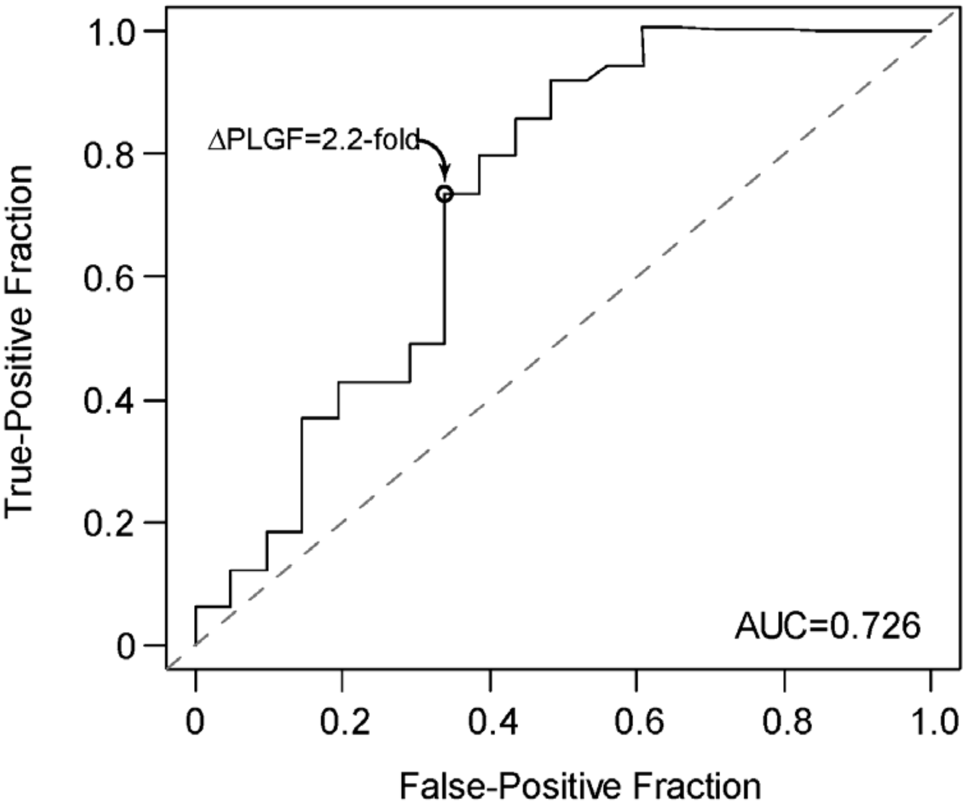

Figure S3. Motesanib exposure during the first 24 hours of treatment as a predictor of 1-year survival.

Receiver operating characteristic curves of 1-hour (**A**) and 24-hour (**B**) exposure to motesanib predicting survival beyond 1 year. AUC=area under the curve.

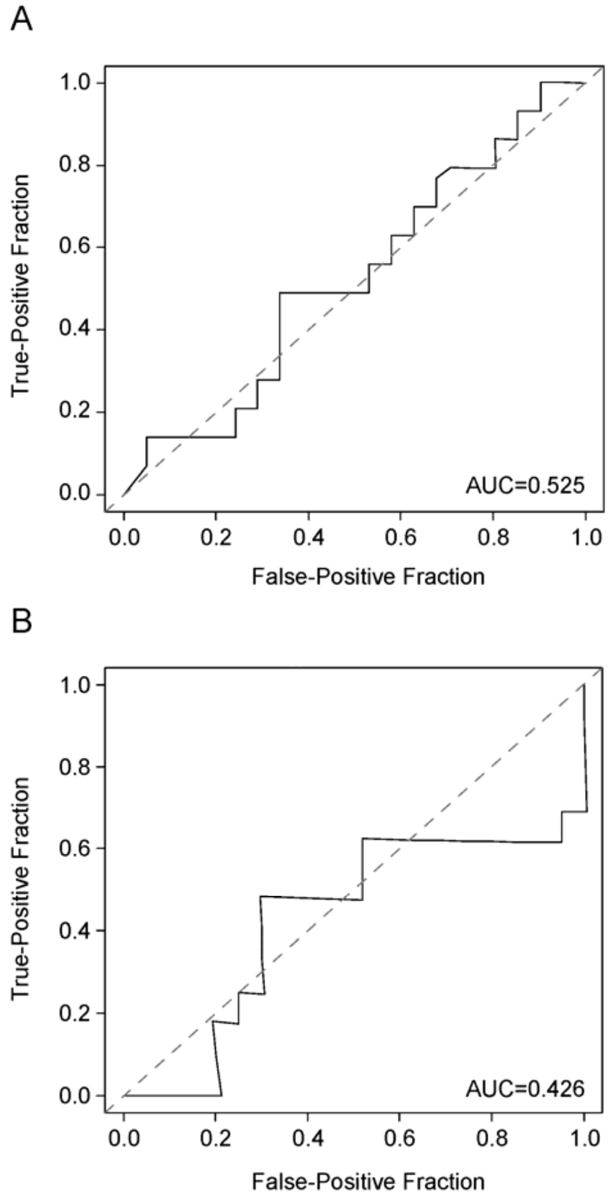

Figure S4. Influence of PLGF assay on evaluation of PLGF as a biomarker.

The MSD and ARCHITECT (plasma and serum) assays were highly concordant (A–C) as indicated by the Lin's concordance correlation (rho) and Pearson coefficient. The PLGF assay used did not influence associations between PLGF and OS or PFS in the phase 2 study (E).

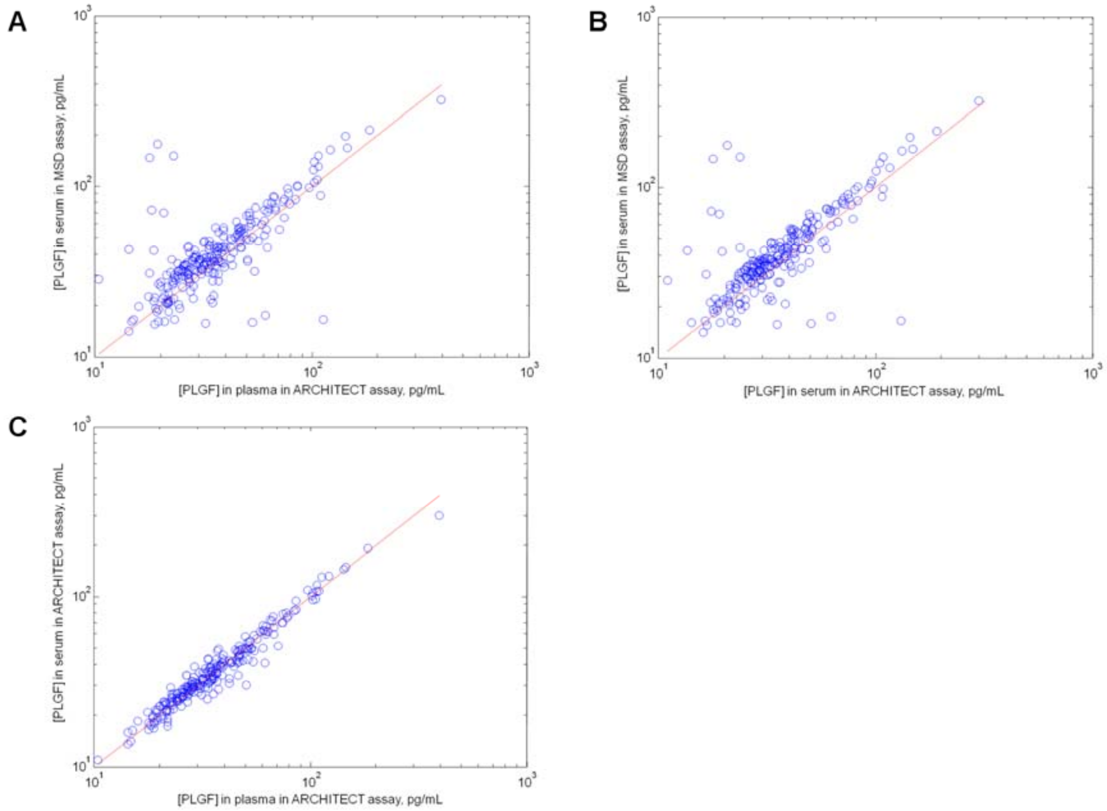

D

PLGF assay	Rho	Pearson
ARCHITECT assay plasma vs. MSD assay serum	0.814	0.828
ARCHITECT assay serum vs. MSD assay serum	0.807	0.835
ARCHITECT assay plasma vs. ARCHITECT assay serum	0.970	0.975

E

PLGF assay	Cutoff	OS *P*-value	PFS *P*-value
MSD assay serum	2.2	0.008	0.016
ARCHITECT assay plasma	2.2	0.016	0.024
ARCHITECT assay serum	2.4	0.016	0.009

Table S1. Continuous Cox Proportional Hazards Models for Progression-Free Survival and Overall Survival Incorporating PLGF, 1-Hour Motesanib Exposure and/or 24-Hour Motesanib Exposure

	***P*-values**		
**Model**	**PLGF**	**1-h Motesanib PK Exposure**	**24-h Motesanib PK Exposure**
Progression-free survival			
PLGF	0.002		
1-h PK		0.720	
24-h PK			0.975
PLGF + 1-h PK	0.013	0.962	
PLGF + 24-h PK	0.009		0.466
1-h PK + 24-h PK		0.788	0.730
PLGF + 1-h PK + 24-h PK	0.021	0.754	0.737
Overall survival			
PLGF	0.018		
1-h PK		0.603	
24-h PK			0.676
PLGF + 1-h PK	0.041	0.464	
PLGF + 24-h PK	0.088		0.777
1-h PK + 24-h PK		0.918	0.940
PLGF + 1-h PK + 24-h PK	0.117	0.654	0.838

*Data are for samples from the phase 2 study evaluated for PLGF using the MSD assay system.

Table S2. Concordance Between the MSD and ARCHITECT Assays*

		ARCHITECT Assay	
	Samples, n (%)	PLGF <2.0-fold	PLGF >2.0-fold
**MSD Assay**	PLGF >2.0-fold	3 (8)	17 (46)
	PLGF <2.0-fold	15 (41)	2 (5)

*Data are for samples from the phase 2 study evaluated for PLGF using both assay systems.

Table S3. Placental Growth Factor (PLGF) Concentrations at Baseline and at Study Week 4 in MONET1

	Baseline*			Baseline†				Week 4			
**PLGF**	**Arm A**	**Arm B**	**All Patients**	**Arm A**	**Arm A ΔPLGF <2.0-fold**	**Arm A ΔPLGF >2.0-fold**	**l.m,j vArm B**	**Arm A**	**Arm A ΔPLGF <2.0-fold**	**Arm A ΔPLGF >2.0-fold**	**Arm B**
N	453	465	918	386	156	230	414	386	156	230	414
Median	24.40	24.70	24.50	24.30	25.00	23.30	24.45	57.35	35.65	80.40	25.60
Range	8.80–154.3	13.80–137.0	8.80–154.3	8.80–143.7	13.70–143.7	8.80–54.20	13.80–137	18.30–326.8	18.30–113.9	29.30–326.8	15.20–182.5
Mean	26.79	26.33	26.55	26.04	28.27	24.52	26.22	74.02	38.95	97.81	27.63
SD	12.09	10.23	11.18	9.89	13.10	6.52	10.53	51.67	14.59	54.21	11.75

*PLGF concentration at baseline for all patients with available samples.

†PLGF concentration at baseline for all patients with a matching sample at study week 4.
