# Supplementary material for: Challenges in Developing a Validated Biomarker for Angiogenesis Inhibitors: The Motesanib Experience
Source: PLoS One. 2014 Oct 14;9(10):e108048. doi: 10.1371/journal.pone.0108048 (PMC4196848; doi:10.1371/journal.pone.0108048)
Supplement: Appendix S2 — Contains patient-level data from the phase 2 study. (PDF) [file pone.0108048.s002.pdf]

Patient ID	Treatment Cohort	Best response per RECIST	PFS, days	PFS censor flag	OS, days	OS censor flag	Baseline PLGF, pg/mL	Week 4 PLGF, pg/mL
pt1	Motesanib 75 mg BID	SD	117	1	117	1		
pt2	Motesanib 125 mg QD	ND	564	1	564	1	39.4921	
pt3	Motesanib 125 mg QD	SD	124	0	125	0	29.9896	96.3271
pt4	Motesanib 75 mg BID	ND	39	1	39	1	23.1554	
pt5	Bevacizumab 15 mg/kg Q3W	SD	107	0	452	0	38.0263	43.9191
pt6	Bevacizumab 15 mg/kg Q3W	SD	464	1	464	1	34.1334	57.5684
pt7	Motesanib 75 mg BID	PR	235	1	551	0	32.1938	43.9733
pt8	Motesanib 125 mg QD	PR	471	0	577	0	34.5121	213.2826
pt9	Bevacizumab 15 mg/kg Q3W	SD	129	1	504	1	32.4376	36.1963
pt10	Motesanib 75 mg BID	SD	128	1	174	1	28.8379	27.5466
pt11	Bevacizumab 15 mg/kg Q3W	PR	510	1	510	1	24.2057	31.0034
pt12	Bevacizumab 15 mg/kg Q3W	SD	209	0	441	0	20.5479	30.2058
pt13	Motesanib 75 mg BID	SD	71	1	71	1	21.8776	16.0367
pt14	Motesanib 75 mg BID	SD	115	1	115	1	42.1578	37.8223
pt15	Motesanib 75 mg BID	SD	88	0	214	0		
pt16	Bevacizumab 15 mg/kg Q3W	PR	342	1	637	0		
pt17	Motesanib 125 mg QD	ND	1	0	17	0	26.5406	
pt18	Bevacizumab 15 mg/kg Q3W	PD	43	1	105	1	15.6643	15.8292
pt19	Bevacizumab 15 mg/kg Q3W	SD	133	1	233	1	177.1179	17.5057
pt20	Motesanib 125 mg QD	SD	224	1	224	1	28.6253	16.6679
pt21	Bevacizumab 15 mg/kg Q3W	ND	46	1	46	1		
pt22	Motesanib 125 mg QD	PD	93	1	244	1		
pt23	Motesanib 75 mg BID	SD	132	1	225	1		
pt24	Motesanib 125 mg QD	PR	151	1	151	1	22.9955	34.4258
pt25	Motesanib 75 mg BID	SD	155	1	155	1	42.6057	69.8726
pt26	Bevacizumab 15 mg/kg Q3W	PR	149	0	505	0	73.2003	44.4852
pt27	Motesanib 125 mg QD	SD	94	0	494	0	16.6299	28.5503
pt28	Motesanib 75 mg BID	PR	308	1	308	1	15.5284	20.8145
pt29	Motesanib 125 mg QD	PD	64	1	124	1	39.3282	52.6336
pt30	Bevacizumab 15 mg/kg Q3W	SD	47	0	459	0	34.2416	98.5254
pt31	Motesanib 75 mg BID	ND	242	1	242	1	36.8837	
pt32	Bevacizumab 15 mg/kg Q3W	PR	155	0	443	0	18.4895	19.7978
pt33	Bevacizumab 15 mg/kg Q3W	ND	6	1	6	1	29.8875	
pt34	Bevacizumab 15 mg/kg Q3W	SD	92	1	151	1	21.2036	37.7252
pt35	Motesanib 75 mg BID	ND	10	1	10	1	40.6488	
pt36	Motesanib 75 mg BID	SD	96	1	158	1	43.045	35.9657
pt37	Bevacizumab 15 mg/kg Q3W	PD	42	1	113	0	14.1905	21.8361
pt38	Motesanib 75 mg BID	SD	175	1	322	1	35.4878	
pt39	Motesanib 75 mg BID	SD	130	1	130	1	146.7477	151.8022
pt40	Motesanib 75 mg BID	PR	86	0	451	0	21.7696	27.3941
pt41	Motesanib 125 mg QD	ND	256	1	256	1	20.8301	40.9678
pt42	Bevacizumab 15 mg/kg Q3W	ND	56	1	56	1	63.0674	48.939

Patient ID	Treatment Cohort	Best response per RECIST	PFS, days	PFS censor flag	OS, days	OS censor flag	Baseline PLGF, pg/mL	Week 4 PLGF, pg/mL
pt43	Motesanib 75 mg BID	SD	470	0	793	0	30.9624	45.0691
pt44	Motesanib 125 mg QD	PR	464	0	793	0	45.828	
pt45	Motesanib 75 mg BID	SD	169	1	240	1		
pt46	Motesanib 125 mg QD	PR	438	1	438	1	44.1358	167.1041
pt47	Bevacizumab 15 mg/kg Q3W	SD	267	1	267	1	25.8667	42.2129
pt48	Bevacizumab 15 mg/kg Q3W	SD	42	0	544	0	29.2142	31.7347
pt49	Motesanib 125 mg QD	SD	137	1	137	1	52.1218	83.7487
pt50	Bevacizumab 15 mg/kg Q3W	SD	373	1	373	1	23.0428	51.4169
pt51	Motesanib 75 mg BID	ND	253	1	253	1	35.8287	
pt52	Motesanib 125 mg QD	ND	170	1	170	1		74.7414
pt53	Motesanib 125 mg QD	PD	39	1	147	1	25.4515	56.8678
pt54	Motesanib 125 mg QD	SD	383	1	439	1	32.5444	151.7967
pt55	Motesanib 75 mg BID	PD	38	1	309	1		
pt56	Motesanib 125 mg QD	ND	225	1	225	1		
pt57	Bevacizumab 15 mg/kg Q3W	ND	278	1	278	1		
pt58	Motesanib 125 mg QD	PD	38	1	228	1		
pt59	Motesanib 125 mg QD	ND	284	1	284	1		
pt60	Bevacizumab 15 mg/kg Q3W	ND	262	1	262	1		
pt61	Motesanib 125 mg QD	PR	439	1	687	1	21.9	48.2269
pt62	Motesanib 125 mg QD	SD	292	1	684	1	20.7559	38.143
pt63	Motesanib 75 mg BID	SD	524	1	524	1	21.0764	36.7403
pt64	Motesanib 125 mg QD	PR	249	0	515	0	18.8819	80.3631
pt65	Bevacizumab 15 mg/kg Q3W	SD	85	0	829	0		
pt66	Motesanib 75 mg BID	SD	91	1	105	1	37.9724	31.7226
pt67	Bevacizumab 15 mg/kg Q3W	PR	249	1	249	1	21.2497	36.0954
pt68	Bevacizumab 15 mg/kg Q3W	SD	45	0	638	0	17.2886	
pt69	Motesanib 75 mg BID	PD	37	1	730	0	34.1845	32.6265
pt70	Motesanib 75 mg BID	ND	144	1	144	1	33.8462	43.2546
pt71	Motesanib 125 mg QD	SD	279	1	279	1	41.864	108.9799
pt72	Motesanib 125 mg QD	SD	83	1	123	1	30.7949	62.0081
pt73	Motesanib 75 mg BID	ND	143	1	143	1	31.76	47.0142
pt74	Bevacizumab 15 mg/kg Q3W	PR	387	0	490	0	36.9282	78.9805
pt75	Motesanib 125 mg QD	SD	137	1	486	1	42.4122	56.2574
pt76	Bevacizumab 15 mg/kg Q3W	PR	284	1	284	1	42.3714	
pt77	Motesanib 125 mg QD	ND	180	1	180	1	33.6571	
pt78	Motesanib 125 mg QD	ND	63	1	63	1	27.4952	
pt79	Motesanib 75 mg BID	SD	45	0	576	0	18.4497	34.515
pt80	Motesanib 75 mg BID	SD	390	1	390	1		
pt81	Motesanib 125 mg QD	ND	1	0	1	0		
pt82	Motesanib 75 mg BID	PR	352	1	469	1	26.0186	59.6276
pt83	Motesanib 75 mg BID	PR	171	1	171	1		
pt84	Motesanib 125 mg QD	SD	44	0	145	0	40.1623	

Patient ID	Treatment Cohort	Best response per RECIST	PFS, days	PFS censor flag	OS, days	OS censor flag	Baseline PLGF, pg/mL	Week 4 PLGF, pg/mL
pt85	Bevacizumab 15 mg/kg Q3W	PR	251	1	251	1	35.0489	63.7117
pt86	Motesanib 125 mg QD	PR	85	0	486	0		
pt87	Motesanib 75 mg BID	ND	1	0	18	0		
pt88	Bevacizumab 15 mg/kg Q3W	SD	91	1	92	0		
pt89	Bevacizumab 15 mg/kg Q3W	SD	132	1	132	1	28.3125	30.7258
pt90	Bevacizumab 15 mg/kg Q3W	PR	199	1	199	1	53.12	43.4105
pt91	Bevacizumab 15 mg/kg Q3W	SD	90	0	705	0	19.1928	43.533
pt92	Motesanib 75 mg BID	ND	388	1	388	1		
pt93	Motesanib 125 mg QD	ND	1	0	663	0	35.8394	47.9907
pt94	Bevacizumab 15 mg/kg Q3W	SD	279	1	279	1	33.9737	
pt95	Bevacizumab 15 mg/kg Q3W	ND	430	1	430	1		
pt96	Motesanib 125 mg QD	SD	723	1	723	1	39.3373	
pt97	Motesanib 125 mg QD	SD	194	0	881	0	16.4606	47.433
pt98	Bevacizumab 15 mg/kg Q3W	PR	516	1	516	1	28.0655	36.0781
pt99	Bevacizumab 15 mg/kg Q3W	PR	521	1	521	1	32.8363	
pt100	Motesanib 75 mg BID	SD	137	0	478	0	14.3926	
pt101	Motesanib 75 mg BID	SD	43	0	561	0	23.5181	
pt102	Motesanib 125 mg QD	SD	57	1	57	1	32.1757	73.2136
pt103	Motesanib 125 mg QD	SD	266	1	715	0	37.342	86.954
pt104	Motesanib 75 mg BID	SD	241	1	241	1	37.2779	31.8129
pt105	Bevacizumab 15 mg/kg Q3W	PR	211	1	477	1		
pt106	Motesanib 125 mg QD	ND	41	1	41	1	26.9513	50.1964
pt107	Bevacizumab 15 mg/kg Q3W	PR	426	1	615	1	20.951	50.8519
pt108	Bevacizumab 15 mg/kg Q3W	ND	1	0	1	0		
pt109	Motesanib 125 mg QD	PR	259	1	636	0	29.8248	66.8334
pt110	Motesanib 75 mg BID	SD	92	1	131	0	34.0312	36.3299
pt111	Motesanib 75 mg BID	SD	684	1	684	1	34.7442	34.1288
pt112	Motesanib 75 mg BID	PR	464	1	464	1	54.9439	44.6001
pt113	Motesanib 125 mg QD	PR	206	1	618	1	49.6683	78.7312
pt114	Motesanib 75 mg BID	PR	304	1	707	0	57.9166	50.775
pt115	Motesanib 75 mg BID	PR	300	0	517	0	30.554	35.6132
pt116	Motesanib 75 mg BID	SD	536	1	536	1	25.2312	
pt117	Bevacizumab 15 mg/kg Q3W	PR	219	0	570	0	23.5954	56.0144
pt118	Motesanib 125 mg QD	SD	428	1	428	1	30.7504	99.2637
pt119	Motesanib 125 mg QD	PD	41	1	55	1	34.733	72.9478
pt120	Bevacizumab 15 mg/kg Q3W	ND	80	1	80	1	29.5458	56.0324
pt121	Motesanib 125 mg QD	ND	1	0	1	0		
pt122	Motesanib 125 mg QD	PR	218	0	448	0	36.5254	130.3957
pt123	Motesanib 75 mg BID	SD	171	0	463	0	43.4356	57.0724
pt124	Bevacizumab 15 mg/kg Q3W	SD	81	1	142	1	35.4137	55.8332
pt125	Bevacizumab 15 mg/kg Q3W	PR	128	1	324	1	19.196	33.4755
pt126	Bevacizumab 15 mg/kg Q3W	ND	121	1	121	1		

Patient ID	Treatment Cohort	Best response per RECIST	PFS, days	PFS censor flag	OS, days	OS censor flag	Baseline PLGF, pg/mL	Week 4 PLGF, pg/mL
pt127	Motesanib 75 mg BID	SD	88	1	511	0	21.9719	75.2781
pt128	Motesanib 75 mg BID	PD	35	1	714	1	28.8905	39.522
pt129	Motesanib 75 mg BID	ND	236	1	236	1		
pt130	Bevacizumab 15 mg/kg Q3W	SD	39	0	722	0		
pt131	Motesanib 75 mg BID	ND	461	1	461	1	33.9246	
pt132	Bevacizumab 15 mg/kg Q3W	SD	207	1	207	1		
pt133	Motesanib 75 mg BID	ND	87	1	87	1		
pt134	Motesanib 125 mg QD	PR	395	0	636	0		
pt135	Motesanib 125 mg QD	ND	26	1	26	1		
pt136	Motesanib 75 mg BID	PR	206	1	518	1		
pt137	Motesanib 125 mg QD	PR	265	1	565	0		
pt138	Bevacizumab 15 mg/kg Q3W	PR	227	1	302	1		
pt139	Motesanib 125 mg QD	SD	425	1	425	1		
pt140	Motesanib 75 mg BID	PD	43	1	50	1	47.4155	163.6647
pt141	Bevacizumab 15 mg/kg Q3W	PR	309	1	427	0	31.8999	33.1186
pt142	Bevacizumab 15 mg/kg Q3W	PR	129	0	784	0	25.7548	31.8325
pt143	Motesanib 125 mg QD	ND	162	1	162	1	42.5732	
pt144	Bevacizumab 15 mg/kg Q3W	PR	219	1	679	1	43.7872	65.1049
pt145	Motesanib 75 mg BID	SD	102	1	409	1	62.2267	83.7343
pt146	Motesanib 125 mg QD	SD	86	1	86	1	29.184	
pt147	Bevacizumab 15 mg/kg Q3W	PR	417	1	417	1	35.8481	43.6233
pt148	Bevacizumab 15 mg/kg Q3W	ND	709	1	709	1	80.7409	88.4968
pt149	Bevacizumab 15 mg/kg Q3W	SD	425	1	425	1	43.7982	70.2134
pt150	Motesanib 125 mg QD	PD	82	1	236	1	49.5383	85.4534
pt151	Motesanib 75 mg BID	SD	609	1	609	1	20.2481	27.4572
pt152	Motesanib 125 mg QD	SD	88	1	120	1	25.5658	31.5602
pt153	Bevacizumab 15 mg/kg Q3W	SD	183	1	479	1	37.6581	60.2594
pt154	Motesanib 125 mg QD	SD	51	0	544	0	41.6518	36.7018
pt155	Motesanib 125 mg QD	PR	234	1	234	1	26.4864	89.9388
pt156	Motesanib 125 mg QD	PR	316	1	316	1	39.7809	55.1509
pt157	Motesanib 75 mg BID	PD	86	1	497	0	33.0239	73.9522
pt158	Motesanib 75 mg BID	PR	183	1	448	1	35.3793	125.4666
pt159	Motesanib 125 mg QD	PD	38	1	795	0	31.1466	140.0067
pt160	Bevacizumab 15 mg/kg Q3W	SD	616	0	695	0	31.9818	54.1933
pt161	Motesanib 75 mg BID	SD	188	1	188	1	29.5425	53.4397
pt162	Motesanib 75 mg BID	SD	83	1	83	1	37.8258	44.4132
pt163	Motesanib 125 mg QD	PR	162	0	470	0	22.5405	320.1481
pt164	Motesanib 125 mg QD	SD	149	1	149	1		
pt165	Motesanib 125 mg QD	ND	62	1	62	1	30.0793	44.6731
pt166	Bevacizumab 15 mg/kg Q3W	PR	311	1	311	1	37.9622	71.2491
pt167	Motesanib 75 mg BID	ND	1	0	23	0	53.2673	
pt168	Motesanib 125 mg QD	PR	461	0	534	0		

Patient ID	Treatment Cohort	Best response per RECIST	PFS, days	PFS censor flag	OS, days	OS censor flag	Baseline PLGF, pg/mL	Week 4 PLGF, pg/mL
pt169	Motesanib 125 mg QD	SD	261	0	719	0	40.7927	198.2995
pt170	Motesanib 75 mg BID	ND	68	1	68	1	46.863	
pt171	Bevacizumab 15 mg/kg Q3W	SD	52	0	521	0	37.8282	105.0052
pt172	Motesanib 75 mg BID	SD	51	0	83	0	22.4179	
pt173	Bevacizumab 15 mg/kg Q3W	ND	1	0	18	0	16.7315	
pt174	Motesanib 125 mg QD	ND	225	1	225	1		
pt175	Motesanib 75 mg BID	PR	158	1	580	1	38.9673	47.3412
pt176	Bevacizumab 15 mg/kg Q3W	PR	214	0	480	0	67.087	100.8412
pt177	Motesanib 75 mg BID	PR	304	0	506	0	27.3066	37.2396
pt178	Motesanib 75 mg BID	PR	381	0	548	0	46.8097	57.3143
pt179	Motesanib 75 mg BID	SD	88	0	544	0	36.6365	48.1666
pt180	Bevacizumab 15 mg/kg Q3W	SD	99	1	99	1		
pt181	Motesanib 125 mg QD	PR	283	1	508	1	16.2345	27.2612
pt182	Bevacizumab 15 mg/kg Q3W	SD	210	1	210	1	16.0903	41.0994
pt183	Motesanib 75 mg BID	PR	207	1	361	1	27.7664	22.1693
pt184	Bevacizumab 15 mg/kg Q3W	SD	83	1	115	1	30.3516	35.2354
pt185	Bevacizumab 15 mg/kg Q3W	SD	186	1	186	1		
pt186	Bevacizumab 15 mg/kg Q3W	PR	317	1	317	1		
