# Supplementary material for: Challenges in Developing a Validated Biomarker for Angiogenesis Inhibitors: The Motesanib Experience
Source: PLoS One. 2014 Oct 14;9(10):e108048. doi: 10.1371/journal.pone.0108048 (PMC4196848; doi:10.1371/journal.pone.0108048)
Supplement: Appendix S3 — Contains patient-level data from the phase 3 study. (PDF) [file pone.0108048.s003.pdf]

Patient ID	Treatment Arm	Best response per RECIST	PFS, days	PFS actual flag	OS, days	OS actual flag	Baseline PLGF, pg/mL	Week 4 PLGF, pg/mL
pt1	Placebo	PR	236	1	318	1	17.7	21.7
pt2	Motesanib 125 mg QD	SD	141	1	172	1	24.7	72.9
pt3	Placebo	ND	34	1	34	1	23.6	39.7
pt4	Placebo	SD	166	1	166	1	18	20.9
pt5	Placebo	SD	42	0	71	0	137	112.3
pt6	Placebo	SD	264	0	317	0	23.3	26.4
pt7	Placebo	PD	41	1	93	0	17.2	21.7
pt8	Motesanib 125 mg QD	ND	35	1	35	1	22	38.8
pt9	Placebo	PR	148	1	157	1	117.9	55.2
pt10	Placebo	SD	90	1	202	1	25.5	25.2
pt11	Placebo	SD	424	1	752	1		17.6
pt12	Motesanib 125 mg QD	ND	1	0	69	0		
pt13	Placebo	ND	43	1	43	1	33.1	
pt14	Motesanib 125 mg QD	PR	537	1	537	1	19.8	
pt15	Placebo	SD	72	1	72	1	24.1	31.6
pt16	Motesanib 125 mg QD	PR	421	0	430	0	48	49.8
pt17	Motesanib 125 mg QD	ND	1	0	44	0		
pt18	Motesanib 125 mg QD	PR	98	1	98	1	30.3	74.1
pt19	Motesanib 125 mg QD	PR	168	1	231	1		23.1
pt20	Motesanib 125 mg QD	SD	43	0	75	0		
pt21	Placebo	PR	140	0	218	0		
pt22	Placebo	PR	310	1	1155	0		
pt23	Placebo	ND	1	0	56	0	18.8	17.5
pt24	Motesanib 125 mg QD	SD	126	1	1007	0	16.2	23.7
pt25	Motesanib 125 mg QD	PR	299	1	435	1	17.9	59.4
pt26	Placebo	PR	194	1	331	1		
pt27	Motesanib 125 mg QD	SD	67	1	67	1		123.2
pt28	Motesanib 125 mg QD	PR	220	1	401	1	23.6	
pt29	Placebo	PR	127	1	337	0	20.9	
pt30	Placebo	PR	207	1	502	1	32.3	28.2
pt31	Placebo	SD	141	1	951	1	25	25.3
pt32	Placebo	PD	41	1	149	1		
pt33	Motesanib 125 mg QD	SD	84	1	179	1	20.1	
pt34	Placebo	SD	305	1	407	1	21.2	31.7
pt35	Placebo	SD	101	0	665	1	20.8	19.5
pt36	Motesanib 125 mg QD	PR	302	1	372	1		59.9
pt37	Motesanib 125 mg QD	SD	89	1	89	1		519.8
pt38	Motesanib 125 mg QD	ND	84	1	84	1		45
pt39	Placebo	SD	73	1	73	1	18.9	28.2
pt40	Motesanib 125 mg QD	PR	268	1	685	1	23.4	62.2
pt41	Placebo	SD	91	1	135	0	21.9	23.8
pt42	Motesanib 125 mg QD	SD	100	1	122	1	24.4	98.9

Patient ID	Treatment Arm	Best response per RECIST	PFS, days	PFS actual flag	OS, days	OS actual flag	Baseline PLGF, pg/mL	Week 4 PLGF, pg/mL
pt43	Motesanib 125 mg QD	ND	7	1	7	1	17.2	
pt44	Placebo	SD	167	1	370	1	24	34.4
pt45	Motesanib 125 mg QD	SD	213	1	484	0	20.3	38.2
pt46	Motesanib 125 mg QD	SD	89	1	331	1	16.7	67.9
pt47	Placebo	SD	167	1	339	1	20.9	25.8
pt48	Placebo	PR	210	1	332	0	29.6	17.3
pt49	Motesanib 125 mg QD	SD	152	1	152	1	36.5	89.7
pt50	Placebo	PR	95	1	95	1		
pt51	Motesanib 125 mg QD	SD	98	1	154	0		
pt52	Placebo	PR	158	1	291	1		
pt53	Placebo	PR	332	1	472	1		
pt54	Placebo	SD	57	1	57	1	24.8	29.8
pt55	Motesanib 125 mg QD	PR	271	1	485	1	26	29
pt56	Motesanib 125 mg QD	ND	83	1	83	1	44.9	89.4
pt57	Motesanib 125 mg QD	SD	126	0	271	1	25	166.7
pt58	Motesanib 125 mg QD	PR	161	0	400	1	23.3	85.2
pt59	Placebo	ND	47	1	47	1	27.6	
pt60	Placebo	SD	135	0	439	1	20.2	
pt61	Placebo	SD	85	1	121	1	19.8	32
pt62	Placebo	SD	120	0	662	1	23.4	17.5
pt63	Placebo	PD	36	1	102	1	29.2	28.6
pt64	Motesanib 125 mg QD	SD	78	1	276	1	28.7	67.4
pt65	Placebo	SD	79	1	264	1	27	31.9
pt66	Motesanib 125 mg QD	PR	224	0	1024	0	31.1	47.8
pt67	Placebo	ND	14	1	14	1	18.6	
pt68	Placebo	PR	164	1	657	1	31.5	35
pt69	Placebo	PD	44	1	913	0	32.6	27
pt70	Placebo	ND	1	0	193	1		
pt71	Motesanib 125 mg QD	SD	69	0	794	1	28.8	33.2
pt72	Placebo	SD	133	1	391	1	23.8	31.7
pt73	Placebo	PR	219	1	1074	0	21.4	
pt74	Motesanib 125 mg QD	ND	19	1	19	1	24.2	
pt75	Placebo	PD	43	1	751	1	20.9	22.7
pt76	Placebo	PR	177	1	556	1	19.5	21.8
pt77	Motesanib 125 mg QD	SD	93	1	414	1	38.8	189.7
pt78	Placebo	SD	147	1	196	1	37	37.3
pt79	Placebo	PR	210	1	713	1	19.6	17.2
pt80	Motesanib 125 mg QD	PR	265	1	514	1	32	67.7
pt81	Motesanib 125 mg QD	PD	86	1	204	1	39.7	80.2
pt82	Motesanib 125 mg QD	SD	132	0	1239	0	29.2	67.2
pt83	Placebo	SD	184	0	1121	1	34	29
pt84	Placebo	ND	81	1	81	1	16.9	

Patient ID	Treatment Arm	Best response per RECIST	PFS, days	PFS actual flag	OS, days	OS actual flag	Baseline PLGF, pg/mL	Week 4 PLGF, pg/mL
pt85	Motesanib 125 mg QD	ND	34	1	34	1	23.8	29.2
pt86	Motesanib 125 mg QD	SD	100	1	606	1		
pt87	Placebo	PR	167	1	238	1	37.2	27.2
pt88	Placebo	PD	47	1	102	1	24.3	26.5
pt89	Motesanib 125 mg QD	ND	9	1	9	1	23.8	
pt90	Placebo	PD	36	1	39	1	29.9	31.2
pt91	Motesanib 125 mg QD	SD	134	0	501	1	24.1	44.9
pt92	Motesanib 125 mg QD	SD	88	1	417	1	32.2	119.4
pt93	Motesanib 125 mg QD	PR	126	0	474	1	26.5	33.6
pt94	Placebo	SD	96	1	351	1	35.6	37.2
pt95	Placebo	SD	208	1	378	1	29.7	22
pt96	Motesanib 125 mg QD	PR	171	1	333	1	29.7	81
pt97	Placebo	PD	41	1	144	1	24	23.8
pt98	Motesanib 125 mg QD	SD	214	1	895	1	29	
pt99	Motesanib 125 mg QD	PR	104	0	373	1		
pt100	Motesanib 125 mg QD	SD	44	0	456	1	29.3	46.9
pt101	Placebo	SD	155	1	237	1	30.8	
pt102	Motesanib 125 mg QD	SD	42	0	197	1	33.3	26.5
pt103	Motesanib 125 mg QD	SD	120	1	821	0		
pt104	Placebo	PR	168	1	192	1	35.6	34.3
pt105	Placebo	ND	31	1	31	1	41.2	34.9
pt106	Placebo	PD	40	1	225	1	32.7	34.5
pt107	Placebo	SD	169	1	361	1	19.8	26.9
pt108	Motesanib 125 mg QD	ND	13	1	13	1		
pt109	Motesanib 125 mg QD	SD	85	0	185	1		
pt110	Motesanib 125 mg QD	ND	1	0	46	0	18.3	29.4
pt111	Motesanib 125 mg QD	ND	71	1	71	1	30.8	
pt112	Placebo	PD	74	1	97	1	16.8	
pt113	Motesanib 125 mg QD	SD	85	0	732	1		
pt114	Placebo	ND	31	1	31	1		
pt115	Placebo	SD	392	1	722	1	19.2	28.4
pt116	Placebo	ND	16	1	16	1	22	
pt117	Motesanib 125 mg QD	PD	43	1	292	1		
pt118	Motesanib 125 mg QD	ND	1	0	240	1	40.7	
pt119	Placebo	ND	52	1	52	1		
pt120	Motesanib 125 mg QD	SD	268	1	268	1	54.2	125.4
pt121	Placebo	PR	227	1	334	1	16.4	19.3
pt122	Motesanib 125 mg QD	PR	442	1	1085	0	14.1	
pt123	Placebo	PD	20	1	44	1	17.8	
pt124	Placebo	ND	1	0	373	1		
pt125	Placebo	ND	1	0	247	1		
pt126	Motesanib 125 mg QD	SD	66	1	66	1		

Patient ID	Treatment Arm	Best response per RECIST	PFS, days	PFS actual flag	OS, days	OS actual flag	Baseline PLGF, pg/mL	Week 4 PLGF, pg/mL
pt127	Motesanib 125 mg QD	SD	90	1	379	0	37	
pt128	Placebo	SD	134	1	303	1	26.4	25.6
pt129	Placebo	SD	142	1	194	1	32.8	36.3
pt130	Placebo	PD	45	1	1080	0	25.6	31.3
pt131	Motesanib 125 mg QD	PR	141	1	309	1	24.8	242.3
pt132	Placebo	PR	222	1	766	1	23.5	24.9
pt133	Placebo	PR	289	1	1045	0	24.1	19.6
pt134	Placebo	SD	164	0	1039	0	23.1	21.5
pt135	Placebo	SD	171	1	171	1	30.5	34.9
pt136	Placebo	PR	169	0	654	1	23.5	25.5
pt137	Placebo	SD	275	1	324	1	33.8	35.6
pt138	Placebo	SD	127	1	383	1	27.1	31.5
pt139	Motesanib 125 mg QD	SD	122	1	122	1	35.9	51.6
pt140	Placebo	PR	164	1	215	1	19.5	23
pt141	Placebo	PR	321	1	952	1	22.9	27.1
pt142	Motesanib 125 mg QD	ND	33	1	33	1	56.1	
pt143	Motesanib 125 mg QD	ND	1	0	331	1		
pt144	Motesanib 125 mg QD	SD	193	1	193	1	11.6	136.1
pt145	Motesanib 125 mg QD	SD	89	1	150	1	48.4	48.7
pt146	Placebo	SD	171	1	764	1	22.2	17.8
pt147	Placebo	SD	154	1	276	1	79.6	57
pt148	Motesanib 125 mg QD	ND	1	0	140	1	8.8	95.5
pt149	Motesanib 125 mg QD	SD	38	0	141	1	23.9	27.3
pt150	Placebo	SD	132	1	544	1	18.2	18.1
pt151	Motesanib 125 mg QD	SD	97	1	421	1	28.3	42.2
pt152	Placebo	SD	85	0	187	1	35.1	36.9
pt153	Motesanib 125 mg QD	PR	101	1	101	1	21.3	30.6
pt154	Motesanib 125 mg QD	SD	157	0	816	0	19.3	48.4
pt155	Motesanib 125 mg QD	PR	162	1	427	1	21.5	86.4
pt156	Placebo	SD	86	1	231	1	47.7	39.1
pt157	Motesanib 125 mg QD	SD	177	1	313	1	27.8	
pt158	Placebo	SD	92	1	432	1		44.5
pt159	Motesanib 125 mg QD	PR	389	1	551	1	16.5	41.3
pt160	Placebo	SD	250	1	337	0	27.7	26.3
pt161	Motesanib 125 mg QD	ND	13	1	13	1		
pt162	Placebo	SD	298	0	949	0	34.3	32
pt163	Motesanib 125 mg QD	SD	127	1	127	1	22.7	50.5
pt164	Placebo	PR	209	1	785	1	26.7	29.1
pt165	Placebo	PR	160	1	926	0	27.6	24.7
pt166	Motesanib 125 mg QD	SD	63	0	501	1	22	28.6
pt167	Motesanib 125 mg QD	SD	80	1	510	1	30.8	250.8
pt168	Placebo	ND	5	1	5	1	26.1	

Patient ID	Treatment Arm	Best response per RECIST	PFS, days	PFS actual flag	OS, days	OS actual flag	Baseline PLGF, pg/mL	Week 4 PLGF, pg/mL
pt169	Motesanib 125 mg QD	SD	41	0	386	1	22.6	40.2
pt170	Motesanib 125 mg QD	PD	43	1	144	1		
pt171	Motesanib 125 mg QD	PD	35	1	856	1	19.4	55.1
pt172	Placebo	PD	34	1	173	0		23.9
pt173	Placebo	SD	566	1	1114	0		32.1
pt174	Placebo	SD	63	1	96	1	26.5	38.4
pt175	Motesanib 125 mg QD	ND	66	1	66	1	32.1	
pt176	Motesanib 125 mg QD	ND	1	0	142	0		
pt177	Placebo	PD	44	1	94	1	23	26.1
pt178	Motesanib 125 mg QD	PR	222	1	241	1	22.3	105.1
pt179	Placebo	PD	37	1	71	1	30.1	38.9
pt180	Motesanib 125 mg QD	SD	192	0	837	0	14.6	29.3
pt181	Placebo	SD	37	0	501	0	22.9	24.9
pt182	Motesanib 125 mg QD	SD	257	1	297	1	40.7	132.1
pt183	Placebo	SD	168	1	376	1	22.4	22.5
pt184	Motesanib 125 mg QD	CR	424	0	455	0	22.8	151.8
pt185	Placebo	SD	37	0	82	0		
pt186	Motesanib 125 mg QD	SD	89	0	204	1	23.3	110.8
pt187	Motesanib 125 mg QD	PR	300	0	323	0	14.3	109.5
pt188	Placebo	SD	53	1	53	1	23.6	23.8
pt189	Placebo	SD	125	1	248	1	22.2	22.8
pt190	Motesanib 125 mg QD	PR	236	1	236	1	20.7	75.1
pt191	Motesanib 125 mg QD	PR	327	1	762	1	25	93.4
pt192	Motesanib 125 mg QD	PD	48	1	51	1	44	50.1
pt193	Placebo	SD	133	1	297	1		19
pt194	Placebo	PR	176	1	778	1	30.9	29.2
pt195	Placebo	PR	925	0	960	0	23.4	25
pt196	Placebo	PR	164	0	285	1	18.5	19.2
pt197	Motesanib 125 mg QD	SD	222	1	222	1	32.4	169.4
pt198	Motesanib 125 mg QD	SD	85	0	418	1	16.7	26.9
pt199	Motesanib 125 mg QD	ND	47	1	47	1	68.8	
pt200	Motesanib 125 mg QD	SD	70	1	98	1	26.9	190.3
pt201	Placebo	SD	93	1	619	1	29.7	26.3
pt202	Placebo	ND	3	1	3	1	30.3	
pt203	Motesanib 125 mg QD	ND	1	0	2	0		
pt204	Placebo	SD	124	1	191	1	17.4	17
pt205	Placebo	ND	14	1	14	1	37.9	
pt206	Placebo	SD	168	1	307	1	37.6	22.2
pt207	Placebo	ND	1	0	58	0	27.3	35
pt208	Motesanib 125 mg QD	SD	55	1	55	1		
pt209	Placebo	SD	291	1	413	0	15.1	15.4
pt210	Motesanib 125 mg QD	PR	384	0	408	0	22.8	82

Patient ID	Treatment Arm	Best response per RECIST	PFS, days	PFS actual flag	OS, days	OS actual flag	Baseline PLGF, pg/mL	Week 4 PLGF, pg/mL
pt211	Placebo	SD	126	1	126	1	14.1	
pt212	Placebo	SD	134	1	134	1	18.5	17.6
pt213	Motesanib 125 mg QD	SD	99	1	996	0	21.8	116.7
pt214	Motesanib 125 mg QD	SD	927	0	949	0	21.3	58.6
pt215	Placebo	SD	185	1	185	1	24.6	23.6
pt216	Placebo	SD	121	1	121	1	17.8	21.2
pt217	Motesanib 125 mg QD	SD	69	0	278	1	35.1	188.5
pt218	Motesanib 125 mg QD	PR	127	1	173	1	22.6	326.8
pt219	Motesanib 125 mg QD	SD	561	0	1010	0	24.5	27.9
pt220	Placebo	SD	141	1	266	1	19.8	32
pt221	Motesanib 125 mg QD	SD	84	1	122	1	22.6	41.6
pt222	Motesanib 125 mg QD	SD	168	0	450	0	21.3	33.5
pt223	Motesanib 125 mg QD	PR	212	1	553	1		
pt224	Motesanib 125 mg QD	ND	32	1	32	1	37.5	117.9
pt225	Motesanib 125 mg QD	PR	170	1	215	1	21.8	273.6
pt226	Placebo	SD	129	1	327	1	24.5	25.3
pt227	Placebo	SD	84	1	249	1	19.4	20.6
pt228	Placebo	SD	79	1	160	1	26.1	27.7
pt229	Motesanib 125 mg QD	PR	168	0	501	1	17.9	54.8
pt230	Motesanib 125 mg QD	SD	105	1	315	1	23.8	44.3
pt231	Motesanib 125 mg QD	PD	46	1	98	1	30.1	88.5
pt232	Motesanib 125 mg QD	PR	256	1	462	0	25.4	185.5
pt233	Placebo	SD	63	1	268	1	16.7	20.9
pt234	Placebo	SD	287	1	410	1	26.1	25.9
pt235	Motesanib 125 mg QD	PD	70	1	110	1	22.3	41.8
pt236	Placebo	PR	282	1	465	1	39.3	29.7
pt237	Placebo	SD	79	1	963	1	22.5	33.4
pt238	Motesanib 125 mg QD	PR	95	0	228	1	19	87.5
pt239	Placebo	SD	84	1	280	1	30.1	31.1
pt240	Placebo	SD	105	1	218	1	39.5	
pt241	Placebo	SD	47	1	47	1	27.7	29.4
pt242	Motesanib 125 mg QD	SD	82	1	223	1	26.5	48.8
pt243	Placebo	SD	84	1	102	1		
pt244	Placebo	PR	301	1	427	0	16.4	17
pt245	Motesanib 125 mg QD	PR	142	0	417	0	18.5	54
pt246	Placebo	PD	37	1	395	0	20.9	25.3
pt247	Motesanib 125 mg QD	PD	79	1	160	1	28.3	
pt248	Motesanib 125 mg QD	SD	96	1	160	1	40.9	58.3
pt249	Motesanib 125 mg QD	SD	101	1	159	1		43.7
pt250	Placebo	PD	44	1	508	0	17.8	22.8
pt251	Placebo	ND	69	1	69	1	24.7	24.5
pt252	Placebo	SD	150	1	453	1	27.7	20.2

Patient ID	Treatment Arm	Best response per RECIST	PFS, days	PFS actual flag	OS, days	OS actual flag	Baseline PLGF, pg/mL	Week 4 PLGF, pg/mL
pt253	Motesanib 125 mg QD	SD	80	1	80	1	20	33
pt254	Motesanib 125 mg QD	SD	127	1	154	1	19.8	39.5
pt255	Motesanib 125 mg QD	CR	379	0	494	0	21.3	95.3
pt256	Motesanib 125 mg QD	ND	28	1	28	1	24.2	236
pt257	Placebo	SD	156	1	191	1	16.8	20.7
pt258	Placebo	SD	157	1	389	0	26.9	25.9
pt259	Motesanib 125 mg QD	SD	242	1	451	0	30.9	77.4
pt260	Placebo	SD	183	1	396	0	30.4	32.5
pt261	Placebo	SD	47	1	47	1	28.2	37.4
pt262	Motesanib 125 mg QD	PR	695	1	717	1	21.9	168.7
pt263	Motesanib 125 mg QD	SD	232	1	360	1	26.6	65.4
pt264	Motesanib 125 mg QD	SD	123	1	178	1	29.3	47
pt265	Placebo	PR	206	1	277	1	21.6	20.9
pt266	Motesanib 125 mg QD	PR	251	1	251	1	28.5	59.4
pt267	Placebo	SD	95	1	216	1	27.9	31.3
pt268	Motesanib 125 mg QD	SD	168	1	234	1	28.6	74.2
pt269	Motesanib 125 mg QD	SD	91	1	264	1	29	41.3
pt270	Placebo	SD	76	1	87	1	24.2	24.1
pt271	Placebo	SD	94	1	392	1	18.8	24.4
pt272	Placebo	SD	313	0	337	0	23.9	20
pt273	Motesanib 125 mg QD	PR	303	0	1081	0	27.9	
pt274	Placebo	SD	106	1	158	1	31.7	32.4
pt275	Placebo	PD	39	1	628	1	26.6	
pt276	Placebo	SD	209	1	719	1	18	17.3
pt277	Placebo	SD	79	1	101	1	28.8	31
pt278	Placebo	PR	153	1	221	1		
pt279	Placebo	PD	82	1	264	1		
pt280	Motesanib 125 mg QD	SD	331	1	659	1	26.7	109.2
pt281	Placebo	PR	631	1	869	1	24	23.6
pt282	Placebo	ND	9	1	9	1		
pt283	Placebo	ND	77	1	77	1		
pt284	Motesanib 125 mg QD	PR	301	1	406	1	17.1	35.9
pt285	Motesanib 125 mg QD	PR	321	1	321	1	22.7	24.6
pt286	Placebo	SD	200	1	200	1		27
pt287	Motesanib 125 mg QD	PR	338	1	401	0	18.4	41.3
pt288	Placebo	SD	208	1	343	0	24.9	
pt289	Placebo	SD	135	1	135	1	25.2	26
pt290	Motesanib 125 mg QD	ND	9	1	9	1	16.1	
pt291	Motesanib 125 mg QD	PR	211	1	886	0	24	103.3
pt292	Motesanib 125 mg QD	PR	278	1	514	1	19	
pt293	Motesanib 125 mg QD	SD	58	1	58	1	22.8	69
pt294	Placebo	SD	235	1	692	1	28.7	27.5

Patient ID	Treatment Arm	Best response per RECIST	PFS, days	PFS actual flag	OS, days	OS actual flag	Baseline PLGF, pg/mL	Week 4 PLGF, pg/mL
pt295	Motesanib 125 mg QD	PR	195	1	275	1	24.8	
pt296	Motesanib 125 mg QD	SD	116	1	180	1	18.7	65.4
pt297	Placebo	SD	189	1	263	1	24.8	25.9
pt298	Motesanib 125 mg QD	SD	1093	1	1123	0	21.2	54.8
pt299	Motesanib 125 mg QD	SD	37	0	273	1	31.5	179
pt300	Motesanib 125 mg QD	ND	5	1	5	1	44.6	
pt301	Placebo	ND	35	1	35	1	31.4	36.8
pt302	Motesanib 125 mg QD	SD	127	1	275	1	35.7	74.5
pt303	Motesanib 125 mg QD	ND	1	0	214	1	22	
pt304	Motesanib 125 mg QD	SD	56	1	56	1	49.5	151.2
pt305	Motesanib 125 mg QD	SD	294	1	1121	0	16.1	24.1
pt306	Placebo	SD	208	1	334	1	36.9	46.9
pt307	Motesanib 125 mg QD	ND	1	0	870	0		
pt308	Placebo	ND	10	1	10	1	21.8	
pt309	Placebo	PR	203	1	470	1	31.7	
pt310	Placebo	ND	47	1	47	1	22.3	18.6
pt311	Motesanib 125 mg QD	SD	39	0	42	0		
pt312	Placebo	SD	177	1	823	0	22.3	22.3
pt313	Motesanib 125 mg QD	SD	140	1	266	1	41.5	44.1
pt314	Motesanib 125 mg QD	PR	169	1	236	1		
pt315	Motesanib 125 mg QD	PR	178	1	183	0		
pt316	Placebo	ND	1	0	28	0		
pt317	Placebo	PD	67	1	858	0	30.9	
pt318	Placebo	SD	165	1	391	1	29.1	28.6
pt319	Placebo	SD	127	0	669	1	32.7	32.4
pt320	Motesanib 125 mg QD	PR	215	1	787	1	38.9	113.4
pt321	Motesanib 125 mg QD	SD	99	1	101	0	48.2	54.4
pt322	Motesanib 125 mg QD	PD	43	1	181	1	50.8	64.5
pt323	Motesanib 125 mg QD	PD	35	1	55	1	39	
pt324	Placebo	SD	44	0	189	1		
pt325	Placebo	SD	85	1	132	1	37	25.6
pt326	Placebo	PR	175	1	465	0	35.5	36.5
pt327	Motesanib 125 mg QD	PR	137	0	353	0	35.5	125.8
pt328	Motesanib 125 mg QD	SD	164	0	266	1	19.4	62.8
pt329	Placebo	PD	45	1	88	1	30	28
pt330	Motesanib 125 mg QD	SD	41	0	166	1	24.6	191.2
pt331	Placebo	SD	85	1	144	1	28	24.1
pt332	Motesanib 125 mg QD	PD	59	1	317	1	28.4	28.7
pt333	Placebo	PR	177	1	1030	0	17.1	21.5
pt334	Motesanib 125 mg QD	PD	43	1	81	1	26.5	51.1
pt335	Motesanib 125 mg QD	PR	129	1	155	1	22.6	27
pt336	Placebo	PD	43	1	130	1	34.8	37.7

Patient ID	Treatment Arm	Best response per RECIST	PFS, days	PFS actual flag	OS, days	OS actual flag	Baseline PLGF, pg/mL	Week 4 PLGF, pg/mL
pt337	Motesanib 125 mg QD	SD	186	0	662	1	22.1	151.8
pt338	Motesanib 125 mg QD	PR	149	1	149	1	27.4	46.1
pt339	Motesanib 125 mg QD	SD	96	1	260	1	41.7	50.1
pt340	Placebo	SD	188	1	203	1	17.2	15.5
pt341	Placebo	PR	492	1	1018	1	31.4	25.2
pt342	Motesanib 125 mg QD	SD	45	0	64	0		
pt343	Placebo	SD	174	0	453	1	26.2	21.1
pt344	Placebo	SD	128	0	129	0		
pt345	Motesanib 125 mg QD	SD	204	0	1093	1	28.8	40.8
pt346	Motesanib 125 mg QD	PR	192	1	332	1	37.8	60.2
pt347	Motesanib 125 mg QD	PR	308	1	968	0	24.7	102.7
pt348	Motesanib 125 mg QD	SD	203	1	905	0	42.3	42.8
pt349	Motesanib 125 mg QD	SD	125	1	421	1	19.8	36.2
pt350	Motesanib 125 mg QD	SD	147	1	200	1	31.3	91.8
pt351	Motesanib 125 mg QD	SD	107	0	250	1	37	68.4
pt352	Placebo	SD	80	0	550	0	21.3	34.1
pt353	Placebo	SD	78	0	535	0		
pt354	Placebo	SD	108	1	108	1	29.8	30.7
pt355	Placebo	PR	420	1	791	1	49.5	45.8
pt356	Motesanib 125 mg QD	PD	37	1	255	1	19.2	27.2
pt357	Motesanib 125 mg QD	ND	13	1	13	1	23.8	
pt358	Motesanib 125 mg QD	SD	258	1	314	1	25.7	25.6
pt359	Motesanib 125 mg QD	SD	132	1	378	0	21	88.1
pt360	Motesanib 125 mg QD	PR	300	0	323	0	49.8	78.6
pt361	Placebo	SD	807	1	960	0	25.2	30.5
pt362	Placebo	PR	208	1	470	1		
pt363	Motesanib 125 mg QD	SD	195	1	639	1		
pt364	Placebo	PR	159	1	381	1		
pt365	Placebo	ND	6	1	6	1		
pt366	Placebo	PR	225	1	458	1		
pt367	Placebo	SD	95	1	469	0		
pt368	Placebo	PR	268	1	352	0		
pt369	Placebo	SD	182	1	292	1		
pt370	Motesanib 125 mg QD	PR	198	1	393	0		
pt371	Motesanib 125 mg QD	ND	1	0	44	0		
pt372	Motesanib 125 mg QD	SD	158	1	158	1	18.8	24.6
pt373	Motesanib 125 mg QD	SD	225	1	428	0	25.5	25.3
pt374	Motesanib 125 mg QD	PR	169	1	270	1	22.5	43.5
pt375	Placebo	SD	170	1	329	1	18.3	34.9
pt376	Placebo	SD	128	1	209	1	36	27.9
pt377	Motesanib 125 mg QD	SD	129	1	218	1		26.1
pt378	Placebo	SD	128	0	459	1		

Patient ID	Treatment Arm	Best response per RECIST	PFS, days	PFS actual flag	OS, days	OS actual flag	Baseline PLGF, pg/mL	Week 4 PLGF, pg/mL
pt379	Placebo	SD	85	1	142	1		30.6
pt380	Placebo	SD	97	0	255	1		
pt381	Motesanib 125 mg QD	PR	127	0	727	1	28.7	91.4
pt382	Placebo	PD	44	1	82	0	19	26
pt383	Motesanib 125 mg QD	SD	92	1	422	1	28.4	66.2
pt384	Motesanib 125 mg QD	SD	83	1	120	1	29.3	34.6
pt385	Placebo	PR	175	1	416	1	57.7	87.5
pt386	Placebo	SD	134	1	134	1	24.1	23.1
pt387	Motesanib 125 mg QD	PR	232	0	407	0	25	58.5
pt388	Motesanib 125 mg QD	PR	340	1	382	0	16.8	30.6
pt389	Placebo	SD	217	1	217	1	21.7	21.3
pt390	Motesanib 125 mg QD	PR	169	1	339	1	24.4	22.8
pt391	Placebo	SD	164	1	252	1	22.7	32.6
pt392	Placebo	ND	39	1	39	1	27.4	
pt393	Placebo	ND	52	1	52	1	24.8	27.9
pt394	Motesanib 125 mg QD	SD	168	1	363	1	27.2	
pt395	Motesanib 125 mg QD	SD	125	1	449	0	21.8	67.4
pt396	Placebo	SD	125	1	392	1	25.1	25
pt397	Motesanib 125 mg QD	PR	169	1	449	0	15.9	69.3
pt398	Placebo	SD	176	1	407	0	20.9	29.7
pt399	Placebo	SD	83	1	137	1	26.3	28.4
pt400	Motesanib 125 mg QD	PR	217	1	379	0	27	64.5
pt401	Motesanib 125 mg QD	SD	177	1	261	1	29.1	71
pt402	Placebo	PR	176	1	185	1	37.1	24
pt403	Placebo	PR	282	1	344	0	20.9	24.6
pt404	Motesanib 125 mg QD	SD	96	0	353	0	17.7	203.7
pt405	Placebo	PD	42	1	116	1	47.5	36.3
pt406	Motesanib 125 mg QD	SD	168	0	563	0	25	25.3
pt407	Placebo	SD	129	0	245	1	25.5	22.4
pt408	Placebo	SD	85	1	269	1	26.5	31.3
pt409	Motesanib 125 mg QD	PR	236	1	236	1		92.9
pt410	Placebo	SD	101	1	110	1	39.6	83.6
pt411	Motesanib 125 mg QD	SD	83	1	114	0		
pt412	Placebo	PR	212	1	345	1		
pt413	Motesanib 125 mg QD	PR	464	1	582	1	27	
pt414	Placebo	PR	166	0	334	1		
pt415	Motesanib 125 mg QD	PR	349	1	906	1	23	19.8
pt416	Motesanib 125 mg QD	PR	225	1	225	1	37.5	63.9
pt417	Placebo	SD	889	1	940	0	25.4	26
pt418	Motesanib 125 mg QD	PR	470	1	470	1	23.8	54.7
pt419	Motesanib 125 mg QD	PR	252	1	545	1	23.6	49.4
pt420	Motesanib 125 mg QD	SD	86	1	540	1	21.9	46.2

Patient ID	Treatment Arm	Best response per RECIST	PFS, days	PFS actual flag	OS, days	OS actual flag	Baseline PLGF, pg/mL	Week 4 PLGF, pg/mL
pt421	Placebo	PR	433	1	820	0	23.9	24.8
pt422	Motesanib 125 mg QD	SD	83	1	743	1	17.5	24
pt423	Placebo	SD	212	1	312	1	20.2	24.5
pt424	Motesanib 125 mg QD	PR	391	1	463	0	19.8	90.2
pt425	Placebo	PR	216	1	346	1	39.1	39.5
pt426	Placebo	PR	209	1	386	1	22	22.1
pt427	Placebo	ND	86	1	86	1		
pt428	Placebo	PR	436	0	438	0	18.9	24.8
pt429	Motesanib 125 mg QD	PD	44	1	90	1	27.9	
pt430	Placebo	SD	170	1	232	1	51.3	
pt431	Motesanib 125 mg QD	PR	419	0	421	0	17.6	
pt432	Placebo	PR	218	1	414	0	21.3	
pt433	Placebo	ND	10	1	10	1	34.6	
pt434	Placebo	SD	142	1	255	1	25.4	21.7
pt435	Motesanib 125 mg QD	ND	32	1	32	1	64.5	
pt436	Placebo	PR	302	0	332	0	28.2	31.1
pt437	Placebo	SD	151	1	151	1	24.9	24.2
pt438	Placebo	PD	83	1	155	1	26	25.8
pt439	Placebo	PD	49	1	91	1	61.6	44.4
pt440	Motesanib 125 mg QD	SD	83	1	361	1	32.8	27.5
pt441	Placebo	PR	126	0	564	1	25.2	25.7
pt442	Motesanib 125 mg QD	PD	43	1	256	1	14.7	28.1
pt443	Placebo	CR	725	1	1089	0	21.9	
pt444	Placebo	SD	340	1	652	1	37.5	36.9
pt445	Motesanib 125 mg QD	PR	143	0	161	0		
pt446	Placebo	CR	210	1	357	1	21.1	22.5
pt447	Placebo	SD	152	1	152	1	29.3	39.8
pt448	Motesanib 125 mg QD	PR	427	0	457	0	21.5	35
pt449	Placebo	PR	140	1	260	1	36.7	39
pt450	Placebo	SD	296	1	611	1	25.8	30.5
pt451	Placebo	PR	376	1	449	0	20.2	55.9
pt452	Placebo	PR	251	1	373	0	17	16.4
pt453	Placebo	PR	169	1	223	1	20.4	25.2
pt454	Motesanib 125 mg QD	PD	43	1	109	1	24.7	
pt455	Placebo	ND	1	0	53	0		
pt456	Motesanib 125 mg QD	PR	174	1	366	0	23.4	29.1
pt457	Placebo	PD	38	1	45	1	27.7	18
pt458	Placebo	PD	41	1	102	1	30.2	22
pt459	Placebo	SD	210	1	607	1	14.5	19.6
pt460	Placebo	PD	39	1	122	1	18.6	21.7
pt461	Motesanib 125 mg QD	PR	457	1	505	0	23.5	30.5
pt462	Placebo	PR	177	1	415	0	42.4	

Patient ID	Treatment Arm	Best response per RECIST	PFS, days	PFS actual flag	OS, days	OS actual flag	Baseline PLGF, pg/mL	Week 4 PLGF, pg/mL
pt463	Placebo	PD	44	1	305	1	20.6	23.3
pt464	Placebo	PD	40	1	87	0	26.8	28.4
pt465	Placebo	PR	471	0	479	0	18.8	19.6
pt466	Placebo	SD	93	1	130	1	26.6	25.8
pt467	Motesanib 125 mg QD	SD	217	1	430	0	24.1	40.1
pt468	Placebo	PR	174	1	356	1	22.8	25.3
pt469	Motesanib 125 mg QD	PR	294	1	380	0	21.6	33.3
pt470	Placebo	ND	1	0	43	0		
pt471	Motesanib 125 mg QD	SD	106	1	134	1	26	83.8
pt472	Motesanib 125 mg QD	PR	85	0	185	1	38.2	49.1
pt473	Placebo	SD	85	1	374	0	18.9	22
pt474	Motesanib 125 mg QD	PR	687	1	709	0		
pt475	Placebo	SD	168	1	638	1	29.8	32.7
pt476	Motesanib 125 mg QD	PR	263	1	317	1	19.7	51.3
pt477	Motesanib 125 mg QD	PR	247	1	252	0	25.8	170.8
pt478	Motesanib 125 mg QD	PR	126	0	209	0		
pt479	Placebo	PR	169	1	283	1	23.9	19.6
pt480	Motesanib 125 mg QD	PR	147	1	220	1	22.2	73.9
pt481	Placebo	SD	248	1	403	0	18.2	21.5
pt482	Motesanib 125 mg QD	PR	124	0	403	0	19	63.2
pt483	Placebo	SD	231	1	387	0	19.9	21.3
pt484	Placebo	SD	129	0	183	0	22.9	25.1
pt485	Placebo	SD	121	1	241	1	29.7	24.1
pt486	Motesanib 125 mg QD	ND	1	0	41	0		
pt487	Motesanib 125 mg QD	SD	40	0	82	0		
pt488	Placebo	SD	120	0	137	0		
pt489	Placebo	ND	1	0	274	1		
pt490	Motesanib 125 mg QD	SD	44	0	70	0		
pt491	Placebo	SD	86	1	445	1	26.5	35.8
pt492	Motesanib 125 mg QD	SD	141	1	342	1	21.9	109.8
pt493	Placebo	PR	262	1	394	0		
pt494	Placebo	SD	169	0	230	0		
pt495	Motesanib 125 mg QD	SD	149	0	459	1	28	43
pt496	Placebo	SD	513	1	872	0	19.4	19.3
pt497	Placebo	SD	127	1	267	1	27.6	26.4
pt498	Motesanib 125 mg QD	PR	127	1	145	0		78.2
pt499	Motesanib 125 mg QD	SD	158	1	190	1		
pt500	Placebo	SD	92	1	277	1	20	22.4
pt501	Motesanib 125 mg QD	PR	454	1	869	0		85.6
pt502	Placebo	SD	44	0	126	0	34.2	25
pt503	Motesanib 125 mg QD	SD	43	0	63	0	26.6	31.1
pt504	Motesanib 125 mg QD	SD	47	0	103	0		

Patient ID	Treatment Arm	Best response per RECIST	PFS, days	PFS actual flag	OS, days	OS actual flag	Baseline PLGF, pg/mL	Week 4 PLGF, pg/mL
pt505	Placebo	PR	131	0	175	0		
pt506	Placebo	SD	126	0	156	0		
pt507	Motesanib 125 mg QD	SD	43	0	81	0	19	215.6
pt508	Motesanib 125 mg QD	SD	85	1	263	1	21.8	72.7
pt509	Placebo	SD	184	1	285	1	36.3	32.2
pt510	Placebo	SD	122	0	135	0	25.8	24.4
pt511	Motesanib 125 mg QD	SD	77	0	114	0	33.5	27.5
pt512	Motesanib 125 mg QD	ND	20	1	20	1	81	
pt513	Placebo	PR	177	1	483	1	36	29.5
pt514	Motesanib 125 mg QD	PR	189	1	658	1	31.6	
pt515	Placebo	PR	330	0	939	0	18.7	33.2
pt516	Motesanib 125 mg QD	SD	135	1	164	1	20.2	150.8
pt517	Placebo	PD	44	1	85	1	39	42.9
pt518	Motesanib 125 mg QD	PR	208	0	337	0	30.2	94.5
pt519	Motesanib 125 mg QD	PD	259	1	568	1	23.3	188.8
pt520	Motesanib 125 mg QD	ND	4	1	4	1	24.3	
pt521	Motesanib 125 mg QD	ND	1	0	20	0		
pt522	Motesanib 125 mg QD	SD	73	0	915	1	29.5	75.6
pt523	Placebo	SD	162	1	302	1	19	20.2
pt524	Motesanib 125 mg QD	PR	127	0	163	0	20.6	48.5
pt525	Placebo	PD	39	1	93	1	30	30.8
pt526	Placebo	SD	40	0	40	0	23.7	24.4
pt527	Placebo	SD	331	1	336	0	25.3	21.6
pt528	Placebo	SD	155	1	247	1	33.8	28.8
pt529	Motesanib 125 mg QD	PR	247	1	254	0	17.3	31.9
pt530	Motesanib 125 mg QD	ND	1	0	28	0		
pt531	Placebo	SD	88	0	99	0	31.9	27.7
pt532	Placebo	PD	43	1	141	1	35.9	37.5
pt533	Placebo	PR	167	1	371	1	21.4	29
pt534	Motesanib 125 mg QD	PR	252	1	388	1	27.9	33
pt535	Placebo	PR	251	1	297	1	22.2	26.5
pt536	Motesanib 125 mg QD	PR	132	1	268	1	23.8	47.6
pt537	Placebo	SD	63	0	528	1	34.8	
pt538	Motesanib 125 mg QD	SD	62	1	62	1	27.3	93.8
pt539	Motesanib 125 mg QD	ND	13	1	13	1	23.6	
pt540	Motesanib 125 mg QD	ND	28	1	28	1	27.7	154.9
pt541	Motesanib 125 mg QD	PR	171	1	248	1	16.3	19.3
pt542	Motesanib 125 mg QD	SD	69	1	69	1	34.3	31.9
pt543	Motesanib 125 mg QD	SD	253	0	352	0	18.5	41.3
pt544	Placebo	PR	172	1	270	1	37.1	26.7
pt545	Motesanib 125 mg QD	PR	166	1	337	0	32	32
pt546	Placebo	ND	25	1	25	1		

Patient ID	Treatment Arm	Best response per RECIST	PFS, days	PFS actual flag	OS, days	OS actual flag	Baseline PLGF, pg/mL	Week 4 PLGF, pg/mL
pt547	Placebo	SD	115	1	186	1	39.9	30.8
pt548	Placebo	SD	178	1	178	1	20.1	21.4
pt549	Motesanib 125 mg QD	ND	20	1	20	1	20.9	
pt550	Motesanib 125 mg QD	PD	52	1	75	1	28.3	61.5
pt551	Motesanib 125 mg QD	ND	1	0	354	1	22.9	77.2
pt552	Placebo	SD	88	0	299	1	23.5	22.3
pt553	Placebo	PR	229	1	779	1	18.6	23
pt554	Placebo	SD	226	0	248	0	26.5	182.5
pt555	Motesanib 125 mg QD	PR	157	0	264	0	24.7	28.2
pt556	Motesanib 125 mg QD	PR	266	0	267	0		
pt557	Motesanib 125 mg QD	SD	133	0	176	0		
pt558	Placebo	SD	252	1	285	1	34	48.8
pt559	Motesanib 125 mg QD	PR	336	0	357	0	23.5	38.3
pt560	Placebo	SD	42	0	64	0		
pt561	Motesanib 125 mg QD	SD	130	0	176	0		
pt562	Motesanib 125 mg QD	PR	248	1	623	1	26.3	101.1
pt563	Placebo	SD	171	1	596	1	24.6	17.9
pt564	Motesanib 125 mg QD	PR	209	1	540	0	15.4	80.6
pt565	Motesanib 125 mg QD	SD	184	1	245	1	37.8	79.3
pt566	Placebo	PD	58	1	229	0	29.4	
pt567	Placebo	SD	127	1	249	1	25.6	30.7
pt568	Placebo	PD	79	1	210	1	28.5	29.9
pt569	Motesanib 125 mg QD	PR	182	1	382	1	24.3	34.6
pt570	Placebo	PD	43	1	84	0		
pt571	Motesanib 125 mg QD	SD	159	1	477	0	27.5	36.3
pt572	Motesanib 125 mg QD	SD	168	1	625	1	25.7	51
pt573	Placebo	PR	211	1	367	1	44.2	40.3
pt574	Placebo	SD	217	1	366	0	27.8	30.1
pt575	Motesanib 125 mg QD	SD	96	1	102	1	21	168.4
pt576	Motesanib 125 mg QD	SD	84	1	820	0	72.9	
pt577	Motesanib 125 mg QD	SD	91	0	604	1		
pt578	Motesanib 125 mg QD	ND	14	1	14	1	21.7	
pt579	Motesanib 125 mg QD	SD	155	0	442	0	24.4	91.2
pt580	Motesanib 125 mg QD	PR	179	0	437	0	24.5	57
pt581	Motesanib 125 mg QD	PR	128	1	132	1	60.5	113.9
pt582	Motesanib 125 mg QD	PR	207	1	624	1	24.9	89.7
pt583	Placebo	PD	37	1	342	0	25.4	22.9
pt584	Placebo	SD	169	1	169	1	24.8	27.1
pt585	Motesanib 125 mg QD	SD	44	0	77	0		
pt586	Placebo	PD	52	1	121	1	34.7	
pt587	Placebo	SD	78	1	93	1	21.3	21
pt588	Placebo	PR	252	1	455	1	20.6	19.6

Patient ID	Treatment Arm	Best response per RECIST	PFS, days	PFS actual flag	OS, days	OS actual flag	Baseline PLGF, pg/mL	Week 4 PLGF, pg/mL
pt589	Placebo	SD	153	0	449	0	29.1	33.8
pt590	Placebo	PD	38	1	329	1	36.8	25.1
pt591	Motesanib 125 mg QD	SD	321	1	856	0	22.6	45.4
pt592	Motesanib 125 mg QD	SD	190	1	190	1		
pt593	Motesanib 125 mg QD	SD	42	0	65	0		
pt594	Placebo	SD	85	1	96	0		
pt595	Placebo	PR	224	1	669	1	21.2	30.5
pt596	Motesanib 125 mg QD	SD	43	0	95	0	24.5	126
pt597	Motesanib 125 mg QD	SD	224	0	396	1	17.3	36.5
pt598	Placebo	SD	119	0	504	0	29.3	24.2
pt599	Motesanib 125 mg QD	PR	171	1	413	1	21.8	19.7
pt600	Motesanib 125 mg QD	SD	138	1	138	1	23.1	98.4
pt601	Placebo	SD	135	1	234	1	19.3	19.9
pt602	Motesanib 125 mg QD	SD	96	1	435	0	20.2	33.7
pt603	Motesanib 125 mg QD	SD	176	1	176	1		
pt604	Placebo	SD	139	0	413	0	17	25.7
pt605	Placebo	SD	184	0	393	0	21	27.4
pt606	Placebo	PR	141	1	375	0	22.9	23.1
pt607	Motesanib 125 mg QD	SD	111	1	371	0	24.7	152.1
pt608	Placebo	PR	297	1	366	0	16.4	27
pt609	Motesanib 125 mg QD	PD	42	1	116	1	18	231.5
pt610	Motesanib 125 mg QD	SD	85	1	458	0	28	44.3
pt611	Motesanib 125 mg QD	SD	37	0	58	0		
pt612	Placebo	SD	135	1	178	1		40.2
pt613	Motesanib 125 mg QD	ND	25	1	25	1		
pt614	Placebo	SD	144	1	248	1		32.2
pt615	Motesanib 125 mg QD	UE	95	1	95	1	26.7	39.6
pt616	Motesanib 125 mg QD	PD	43	1	59	1	29.3	224.4
pt617	Placebo	PD	47	1	115	1	26.5	23.1
pt618	Motesanib 125 mg QD	PD	20	1	46	1	154.3	
pt619	Placebo	PD	16	1	141	1	23.4	
pt620	Motesanib 125 mg QD	ND	42	1	42	1	39.3	
pt621	Placebo	PR	211	1	603	1	51.1	39.9
pt622	Placebo	SD	282	1	327	1	39.9	45.7
pt623	Placebo	SD	564	1	816	0	27.1	28.5
pt624	Motesanib 125 mg QD	ND	45	1	45	1	41.5	
pt625	Placebo	PR	379	1	543	0	32.7	39.1
pt626	Placebo	PR	225	1	451	0	35.2	26.7
pt627	Placebo	SD	127	1	381	0	30.5	26.3
pt628	Motesanib 125 mg QD	PR	169	1	346	1	30.4	39.2
pt629	Motesanib 125 mg QD	SD	127	1	333	0	33.3	196.5
pt630	Placebo	PR	127	0	325	0	24.8	28.4

Patient ID	Treatment Arm	Best response per RECIST	PFS, days	PFS actual flag	OS, days	OS actual flag	Baseline PLGF, pg/mL	Week 4 PLGF, pg/mL
pt631	Placebo	SD	212	1	584	1	22	22.2
pt632	Motesanib 125 mg QD	SD	124	1	239	1	24.7	42.3
pt633	Motesanib 125 mg QD	SD	131	1	512	1	28.3	91.6
pt634	Motesanib 125 mg QD	PR	195	1	637	1	18.6	58.5
pt635	Placebo	ND	29	1	29	1	36.5	31.4
pt636	Motesanib 125 mg QD	ND	35	1	35	1	31.3	82.9
pt637	Placebo	SD	267	1	497	1	19.9	20.5
pt638	Placebo	SD	364	1	1058	0	19.1	19.2
pt639	Placebo	UE	29	0	924	1	29	
pt640	Motesanib 125 mg QD	SD	169	0	954	0	32	231.2
pt641	Motesanib 125 mg QD	SD	88	1	88	1		
pt642	Placebo	PR	348	0	358	0	21.5	25.7
pt643	Placebo	PR	177	1	312	1		
pt644	Motesanib 125 mg QD	PR	412	1	415	1		
pt645	Motesanib 125 mg QD	PR	218	1	836	1		
pt646	Motesanib 125 mg QD	CR	207	1	825	1		
pt647	Motesanib 125 mg QD	SD	210	0	521	1		
pt648	Motesanib 125 mg QD	PR	330	1	572	1		
pt649	Placebo	PR	212	1	495	1		
pt650	Placebo	SD	207	1	931	0		
pt651	Placebo	SD	118	1	152	1		
pt652	Motesanib 125 mg QD	PR	232	1	472	0		
pt653	Motesanib 125 mg QD	SD	262	1	761	1		
pt654	Placebo	SD	478	0	478	0		
pt655	Motesanib 125 mg QD	SD	43	0	187	1		
pt656	Motesanib 125 mg QD	CR	947	0	1017	0	32.1	108.5
pt657	Motesanib 125 mg QD	PR	149	1	749	1	22.2	56.2
pt658	Placebo	PR	204	1	579	1	25.7	26.7
pt659	Motesanib 125 mg QD	PR	196	1	426	0	19.1	42.3
pt660	Motesanib 125 mg QD	PR	138	1	141	0		
pt661	Placebo	PD	50	1	51	0		
pt662	Motesanib 125 mg QD	ND	1	0	1010	0		
pt663	Motesanib 125 mg QD	PR	141	0	610	1	21.9	29.8
pt664	Motesanib 125 mg QD	SD	205	1	436	0	19.8	55.4
pt665	Placebo	PR	167	1	378	1	15.9	15.8
pt666	Motesanib 125 mg QD	SD	94	1	94	1	16.8	
pt667	Motesanib 125 mg QD	PR	177	1	792	1	24	40.1
pt668	Motesanib 125 mg QD	SD	42	0	232	1	21.6	34.3
pt669	Motesanib 125 mg QD	PD	25	1	95	1	29.7	
pt670	Motesanib 125 mg QD	PR	81	0	367	0		418.9
pt671	Placebo	PD	43	1	127	1		30.9
pt672	Motesanib 125 mg QD	PD	43	1	145	1		22.4

Patient ID	Treatment Arm	Best response per RECIST	PFS, days	PFS actual flag	OS, days	OS actual flag	Baseline PLGF, pg/mL	Week 4 PLGF, pg/mL
pt673	Motesanib 125 mg QD	SD	267	1	424	1	24	67.3
pt674	Motesanib 125 mg QD	ND	15	1	15	1	20.3	
pt675	Motesanib 125 mg QD	PR	270	1	315	1	25.5	101.1
pt676	Motesanib 125 mg QD	PR	259	1	453	1	16.5	39.8
pt677	Placebo	ND	62	1	62	1	25.1	
pt678	Placebo	SD	120	1	120	1	26.7	23.7
pt679	Placebo	PR	423	1	1116	0	36.7	38.2
pt680	Motesanib 125 mg QD	SD	246	1	332	1	25.8	53
pt681	Placebo	SD	393	1	997	0	18.1	19.7
pt682	Motesanib 125 mg QD	PD	41	1	135	1	28	
pt683	Motesanib 125 mg QD	SD	209	1	209	1	31.7	51.5
pt684	Motesanib 125 mg QD	ND	20	1	20	1	25.9	
pt685	Placebo	SD	134	0	857	0	22.1	21.2
pt686	Motesanib 125 mg QD	PR	168	1	373	0	21.3	48.1
pt687	Motesanib 125 mg QD	PR	138	1	199	1	32.2	35.1
pt688	Motesanib 125 mg QD	SD	130	1	336	0	25.7	33.8
pt689	Motesanib 125 mg QD	PR	126	1	180	1	21.8	35.8
pt690	Placebo	SD	78	1	141	1	24.4	21.2
pt691	Motesanib 125 mg QD	PR	126	1	823	1	16.5	118.9
pt692	Motesanib 125 mg QD	SD	219	0	1033	0	18.1	36.8
pt693	Placebo	SD	38	0	223	1	20.8	29.1
pt694	Motesanib 125 mg QD	PR	126	0	869	1	23	18.3
pt695	Placebo	SD	168	1	406	1	19.2	15.2
pt696	Placebo	PD	24	1	77	1	18.2	
pt697	Placebo	ND	1	0	21	0		
pt698	Motesanib 125 mg QD	PR	254	1	566	1	20.9	61
pt699	Placebo	SD	217	1	450	0	13.8	19
pt700	Placebo	PR	181	1	443	0	22.4	26.8
pt701	Placebo	PD	73	1	694	1	17.9	18.2
pt702	Motesanib 125 mg QD	ND	71	1	71	1	24.1	
pt703	Placebo	SD	115	1	115	1	26.7	
pt704	Placebo	PR	170	1	310	1	30.2	26
pt705	Motesanib 125 mg QD	SD	125	1	125	1	41.6	37.5
pt706	Placebo	ND	1	0	1	0		
pt707	Motesanib 125 mg QD	SD	405	1	574	1		92.9
pt708	Motesanib 125 mg QD	SD	145	1	811	1	37.4	114.6
pt709	Placebo	SD	204	1	204	1	34.9	48.8
pt710	Placebo	SD	180	0	346	0	30.6	26.1
pt711	Placebo	SD	128	0	263	0	37.1	28.9
pt712	Motesanib 125 mg QD	SD	146	1	146	1	27.5	63.6
pt713	Placebo	PR	339	0	366	0		
pt714	Motesanib 125 mg QD	ND	1	0	252	1	24.3	109

Patient ID	Treatment Arm	Best response per RECIST	PFS, days	PFS actual flag	OS, days	OS actual flag	Baseline PLGF, pg/mL	Week 4 PLGF, pg/mL
pt715	Placebo	SD	77	0	325	0		
pt716	Motesanib 125 mg QD	PD	128	1	330	0		
pt717	Motesanib 125 mg QD	SD	121	1	179	1	22.7	89.5
pt718	Placebo	PD	36	1	383	1	34.6	40.7
pt719	Placebo	PD	85	1	153	1		24.2
pt720	Motesanib 125 mg QD	UE	57	1	57	1		231.1
pt721	Placebo	SD	225	1	791	1	20.2	23.9
pt722	Placebo	SD	88	1	88	1	25	21.7
pt723	Placebo	PD	36	1	154	1	26.2	27.5
pt724	Placebo	PD	36	1	105	1	18.3	20.4
pt725	Motesanib 125 mg QD	PR	234	1	635	1	24.1	35.7
pt726	Motesanib 125 mg QD	ND	72	1	72	1	30.7	79.4
pt727	Placebo	SD	103	1	103	1	16.6	22.6
pt728	Placebo	PD	36	1	907	1	24.6	26.8
pt729	Placebo	SD	142	1	337	1	23.9	23.4
pt730	Motesanib 125 mg QD	PR	271	1	365	1	21.7	106.6
pt731	Motesanib 125 mg QD	ND	30	1	30	1	24.6	
pt732	Motesanib 125 mg QD	SD	120	0	269	1	22.8	63.2
pt733	Placebo	PR	182	1	470	1	24.3	25.3
pt734	Motesanib 125 mg QD	SD	121	0	967	0	20.8	92.4
pt735	Motesanib 125 mg QD	ND	1	0	118	1	28.4	
pt736	Motesanib 125 mg QD	PR	133	0	628	1	29.1	107.6
pt737	Placebo	PD	36	1	312	1	31.5	33
pt738	Placebo	PR	641	1	941	0	21.9	23.6
pt739	Placebo	UE	51	0	535	1	32.3	33.5
pt740	Motesanib 125 mg QD	ND	95	1	95	1	28.9	
pt741	Placebo	SD	120	1	225	1	25.5	25.4
pt742	Placebo	SD	113	1	196	1	29.1	27.9
pt743	Motesanib 125 mg QD	ND	35	1	35	1	21.4	
pt744	Placebo	SD	106	1	106	1	28.2	27.1
pt745	Motesanib 125 mg QD	ND	20	1	20	1	19.7	
pt746	Motesanib 125 mg QD	PR	274	0	726	1	21.1	121.1
pt747	Placebo	SD	161	1	177	1	25.5	39.8
pt748	Placebo	PD	36	1	83	1	19.9	27
pt749	Placebo	PR	212	1	827	0	28.4	
pt750	Placebo	SD	52	0	830	0	25.8	
pt751	Motesanib 125 mg QD	PR	680	0	1032	0	43.3	86.3
pt752	Motesanib 125 mg QD	PR	142	1	209	1	29.9	41
pt753	Placebo	PR	202	1	321	1	29.6	31.8
pt754	Motesanib 125 mg QD	ND	1	0	131	1	26.7	
pt755	Motesanib 125 mg QD	SD	39	0	448	1		
pt756	Motesanib 125 mg QD	SD	43	0	119	0		

Patient ID	Treatment Arm	Best response per RECIST	PFS, days	PFS actual flag	OS, days	OS actual flag	Baseline PLGF, pg/mL	Week 4 PLGF, pg/mL
pt757	Motesanib 125 mg QD	ND	1	0	2	0		
pt758	Placebo	SD	78	0	349	1		34.7
pt759	Placebo	PD	43	1	399	0	21.5	20.1
pt760	Placebo	ND	8	1	8	1		
pt761	Placebo	SD	131	0	695	1	20.9	24.3
pt762	Placebo	PR	256	0	789	1	30.6	42.7
pt763	Motesanib 125 mg QD	ND	22	1	22	1	20.4	
pt764	Placebo	ND	1	0	33	0		
pt765	Motesanib 125 mg QD	PR	756	1	886	1	19.4	19.3
pt766	Placebo	PR	170	1	266	1	64.7	29.2
pt767	Placebo	PR	209	1	485	0	16.1	15.9
pt768	Placebo	SD	184	1	241	1	24.9	24.7
pt769	Motesanib 125 mg QD	PR	209	1	231	1	43	71.3
pt770	Motesanib 125 mg QD	PR	289	0	408	0	19.1	59.8
pt771	Motesanib 125 mg QD	SD	127	1	159	1	26.5	64.7
pt772	Placebo	PD	41	1	57	1	23.7	21.1
pt773	Motesanib 125 mg QD	ND	13	1	13	1	19.5	
pt774	Placebo	SD	119	0	250	1	18.6	28.1
pt775	Placebo	PD	81	1	213	1	22.5	24.8
pt776	Motesanib 125 mg QD	PR	217	1	217	1	23	
pt777	Placebo	SD	331	1	583	1	21.1	23.9
pt778	Motesanib 125 mg QD	SD	173	0	823	0	27.4	57.7
pt779	Motesanib 125 mg QD	UE	33	0	345	1	24.4	59
pt780	Placebo	SD	180	1	505	0	37.3	33.2
pt781	Motesanib 125 mg QD	PR	384	1	456	0	20.6	
pt782	Motesanib 125 mg QD	SD	67	0	441	0	26.7	54.1
pt783	Motesanib 125 mg QD	SD	154	1	297	1	21.7	118.5
pt784	Motesanib 125 mg QD	SD	314	0	359	0	17.3	44.6
pt785	Motesanib 125 mg QD	SD	156	1	227	1	30.9	
pt786	Placebo	PD	36	1	409	1	25	
pt787	Placebo	SD	70	0	70	0		
pt788	Motesanib 125 mg QD	PR	516	1	939	0		
pt789	Motesanib 125 mg QD	PD	168	1	289	1	24.3	95.9
pt790	Motesanib 125 mg QD	PD	36	1	62	1	38.2	
pt791	Placebo	SD	52	1	52	1	23.3	28.6
pt792	Placebo	SD	36	0	159	1	23.7	24
pt793	Motesanib 125 mg QD	SD	141	1	237	1	28.6	199.3
pt794	Placebo	PR	254	1	395	1	20.2	23.7
pt795	Motesanib 125 mg QD	PR	219	1	360	0	22.3	145.6
pt796	Motesanib 125 mg QD	SD	86	1	86	1	20.8	37.3
pt797	Placebo	PD	39	1	120	1	34.2	26.6
pt798	Placebo	PR	195	1	373	1	18.5	23.8

Patient ID	Treatment Arm	Best response per RECIST	PFS, days	PFS actual flag	OS, days	OS actual flag	Baseline PLGF, pg/mL	Week 4 PLGF, pg/mL
pt799	Motesanib 125 mg QD	SD	151	1	462	1	40.6	134.3
pt800	Motesanib 125 mg QD	SD	288	1	431	1	24.6	42.9
pt801	Placebo	SD	77	1	179	1		20.9
pt802	Placebo	ND	1	0	327	1		
pt803	Motesanib 125 mg QD	PR	205	1	347	1	39.1	86.1
pt804	Motesanib 125 mg QD	PD	170	1	281	1	20.2	41.1
pt805	Motesanib 125 mg QD	PD	35	1	80	1	29.8	28.1
pt806	Placebo	PD	37	1	263	1	32.2	35.7
pt807	Motesanib 125 mg QD	SD	295	1	448	1	26.2	49.7
pt808	Placebo	SD	178	1	322	1	27.9	
pt809	Placebo	PR	225	1	462	1	24.5	29.7
pt810	Motesanib 125 mg QD	SD	92	1	290	1	26	139.1
pt811	Placebo	PR	179	1	237	1	18.5	21.9
pt812	Placebo	SD	298	1	536	1		
pt813	Placebo	SD	124	0	336	1		
pt814	Motesanib 125 mg QD	SD	393	1	450	1		
pt815	Motesanib 125 mg QD	SD	124	1	240	1		
pt816	Motesanib 125 mg QD	ND	42	1	42	1	41.9	218.8
pt817	Motesanib 125 mg QD	PD	59	1	205	1	33.6	49.8
pt818	Placebo	SD	192	1	266	1	33.9	29.4
pt819	Motesanib 125 mg QD	SD	85	1	825	1	31.1	58.5
pt820	Placebo	SD	196	1	1078	0	20.1	22.7
pt821	Motesanib 125 mg QD	PD	41	1	50	1	31.9	
pt822	Placebo	SD	81	1	178	1	24.3	34.6
pt823	Motesanib 125 mg QD	PD	82	1	135	1	32	73.3
pt824	Placebo	PD	44	1	121	1	34.7	32.8
pt825	Placebo	SD	272	1	447	1	39.1	31.8
pt826	Placebo	SD	129	1	438	1	21	30
pt827	Motesanib 125 mg QD	PR	174	1	385	1	22.9	50.2
pt828	Placebo	PR	263	1	678	1	15.5	16.8
pt829	Motesanib 125 mg QD	SD	86	1	86	1	30.6	49.5
pt830	Motesanib 125 mg QD	ND	34	1	34	1	38.4	79
pt831	Placebo	PR	363	1	932	0	28.1	26
pt832	Motesanib 125 mg QD	PR	149	0	898	0	32.2	105.6
pt833	Motesanib 125 mg QD	SD	127	0	514	0	25.8	53.5
pt834	Motesanib 125 mg QD	SD	135	1	135	1	28.4	54.8
pt835	Placebo	ND	20	1	20	1	20.5	
pt836	Motesanib 125 mg QD	ND	1	0	279	1		
pt837	Motesanib 125 mg QD	ND	48	1	48	1		
pt838	Placebo	SD	97	1	291	1	22.9	21.1
pt839	Motesanib 125 mg QD	PR	116	1	205	1	31.4	134.7
pt840	Placebo	SD	175	1	272	1	30.1	28.2

Patient ID	Treatment Arm	Best response per RECIST	PFS, days	PFS actual flag	OS, days	OS actual flag	Baseline PLGF, pg/mL	Week 4 PLGF, pg/mL
pt841	Motesanib 125 mg QD	PR	492	1	520	0	24	62
pt842	Placebo	SD	299	0	404	1	22.8	24.1
pt843	Motesanib 125 mg QD	SD	220	0	470	1	21.5	74.5
pt844	Motesanib 125 mg QD	PR	165	1	426	1	32.7	59.1
pt845	Motesanib 125 mg QD	SD	215	1	252	1	22.9	110.5
pt846	Motesanib 125 mg QD	SD	43	0	145	1	57.5	60.6
pt847	Placebo	SD	196	1	336	1	21.3	21.5
pt848	Motesanib 125 mg QD	PR	209	1	245	1	20.1	72.6
pt849	Placebo	SD	95	1	95	1	38.5	23.2
pt850	Motesanib 125 mg QD	SD	103	1	217	1		49.4
pt851	Placebo	SD	84	1	119	1	26.4	30.9
pt852	Placebo	PD	49	1	105	1	32.8	41.8
pt853	Placebo	SD	86	1	156	1	29.7	35.7
pt854	Placebo	SD	209	1	209	1	30.3	24.4
pt855	Placebo	PD	47	1	210	1	22.8	19.6
pt856	Placebo	SD	171	1	258	1	27.1	22.7
pt857	Motesanib 125 mg QD	PR	877	1	927	0	20.9	39.3
pt858	Motesanib 125 mg QD	PD	43	1	97	1	35.2	92.4
pt859	Placebo	PR	290	1	1109	0	23.2	28
pt860	Motesanib 125 mg QD	SD	39	0	65	0		
pt861	Placebo	SD	129	0	299	1	26.7	40.8
pt862	Motesanib 125 mg QD	ND	1	0	23	0		
pt863	Motesanib 125 mg QD	SD	141	1	395	1	31.2	20
pt864	Motesanib 125 mg QD	PR	127	1	423	1	42.5	30.2
pt865	Placebo	SD	123	0	473	1	25.5	33
pt866	Placebo	SD	164	1	266	1	27.9	19.6
pt867	Placebo	ND	38	1	38	1	29.1	
pt868	Placebo	SD	298	1	431	1	22.8	28.1
pt869	Motesanib 125 mg QD	PD	43	1	325	1	24.8	57.8
pt870	Placebo	PD	38	1	231	1	27.4	23.8
pt871	Motesanib 125 mg QD	PR	336	0	994	0	18.6	58.2
pt872	Motesanib 125 mg QD	SD	206	1	708	1	21.2	60.5
pt873	Motesanib 125 mg QD	SD	165	1	687	1	28.1	44.2
pt874	Placebo	SD	165	1	871	0	15.3	20.9
pt875	Motesanib 125 mg QD	SD	129	0	340	0	12.3	31.6
pt876	Motesanib 125 mg QD	PR	128	1	336	1	25.8	68.2
pt877	Placebo	SD	374	1	1068	0	26.8	25.3
pt878	Motesanib 125 mg QD	PR	239	1	1064	0	23.7	39.2
pt879	Motesanib 125 mg QD	SD	130	0	1039	0	25.7	148
pt880	Motesanib 125 mg QD	PR	130	1	469	1	15.6	20.2
pt881	Motesanib 125 mg QD	SD	109	1	252	1	20.4	49.2
pt882	Placebo	SD	120	1	872	1		15.7

Patient ID	Treatment Arm	Best response per RECIST	PFS, days	PFS actual flag	OS, days	OS actual flag	Baseline PLGF, pg/mL	Week 4 PLGF, pg/mL
pt883	Motesanib 125 mg QD	PR	164	1	486	1	16.7	67.6
pt884	Placebo	SD	126	1	850	0	15.9	19
pt885	Placebo	SD	160	1	516	1	16.7	20.1
pt886	Motesanib 125 mg QD	UE	43	1	43	1	28.2	
pt887	Placebo	SD	176	1	176	1		
pt888	Motesanib 125 mg QD	SD	213	1	464	0	21.2	23.2
pt889	Motesanib 125 mg QD	PR	248	1	403	0	17.5	53.8
pt890	Placebo	SD	51	1	270	1	20.3	24
pt891	Placebo	PD	33	1	173	1	15.7	16.4
pt892	Motesanib 125 mg QD	ND	1	0	231	1		
pt893	Motesanib 125 mg QD	PR	266	1	961	0	15.6	33.5
pt894	Motesanib 125 mg QD	PR	189	1	449	1	15.7	242.2
pt895	Placebo	SD	206	1	548	1	19.7	23.5
pt896	Motesanib 125 mg QD	SD	591	1	914	0	20.1	133.8
pt897	Motesanib 125 mg QD	PR	174	1	435	1	20.8	56.4
pt898	Placebo	ND	43	1	43	1	36.5	
pt899	Placebo	SD	80	1	828	1	21.5	15.6
pt900	Placebo	PD	39	1	240	1	18	16.7
pt901	Placebo	SD	84	1	203	1	18.2	20.5
pt902	Placebo	PR	129	1	515	1	22.6	25.2
pt903	Placebo	SD	53	1	53	1	23.3	27.6
pt904	Placebo	PD	21	1	63	1	35.7	
pt905	Motesanib 125 mg QD	CR	623	0	1010	0	14.9	104.1
pt906	Motesanib 125 mg QD	PR	119	1	364	1	20.4	49.7
pt907	Placebo	SD	84	1	150	1	27.3	25.3
pt908	Placebo	PD	34	1	112	1	41.5	48
pt909	Placebo	PD	36	1	119	1	20	26.2
pt910	Placebo	SD	169	1	694	1	25	28.3
pt911	Motesanib 125 mg QD	SD	128	1	799	1	24.8	47.8
pt912	Placebo	SD	120	1	420	1	17.6	21.5
pt913	Placebo	SD	331	1	850	0	16.8	21
pt914	Placebo	PD	57	1	165	1	26.1	
pt915	Placebo	SD	79	1	104	1	29.1	26.6
pt916	Placebo	PD	23	1	202	1	17.3	
pt917	Placebo	PD	26	1	108	1	24.7	
pt918	Motesanib 125 mg QD	PR	206	1	1056	0	16.3	34.9
pt919	Motesanib 125 mg QD	ND	82	1	82	1		
pt920	Placebo	PR	163	1	298	1	17.3	21.6
pt921	Motesanib 125 mg QD	PR	207	1	1022	0	20.3	155.4
pt922	Placebo	PR	447	1	1003	0	17.6	24.2
pt923	Placebo	PR	247	1	868	1	21.6	23.5
pt924	Placebo	PD	20	1	187	1	29	

Patient ID	Treatment Arm	Best response per RECIST	PFS, days	PFS actual flag	OS, days	OS actual flag	Baseline PLGF, pg/mL	Week 4 PLGF, pg/mL
pt925	Placebo	PR	414	1	918	0	18.6	20.9
pt926	Motesanib 125 mg QD	PR	250	1	446	1	16.9	29
pt927	Placebo	PD	36	1	904	0	17.5	20.7
pt928	Motesanib 125 mg QD	PR	211	1	893	0	22.5	170.1
pt929	Motesanib 125 mg QD	PR	428	1	885	0	32.1	153
pt930	Placebo	ND	1	0	651	1		
pt931	Motesanib 125 mg QD	PR	112	1	411	1	24.8	35.8
pt932	Placebo	ND	1	0	130	1		
pt933	Placebo	SD	169	1	480	0	15.4	18.9
pt934	Motesanib 125 mg QD	PR	298	1	452	0	15.1	20.8
pt935	Placebo	PR	253	1	445	0	20.2	25.4
pt936	Motesanib 125 mg QD	PR	167	1	401	0	25.7	62.8
pt937	Motesanib 125 mg QD	SD	290	1	401	0	17	32.1
pt938	Placebo	ND	1	0	387	0		
pt939	Placebo	PR	675	1	1040	0	20.2	21.1
pt940	Placebo	SD	135	1	961	1	24.7	22.6
pt941	Motesanib 125 mg QD	ND	7	1	7	1	19.8	
pt942	Placebo	SD	79	1	174	1	28	22.3
pt943	Placebo	SD	207	1	331	1	16.4	19.1
pt944	Motesanib 125 mg QD	PR	427	1	648	1	18	38.9
pt945	Motesanib 125 mg QD	ND	2	1	2	1	22.3	
pt946	Motesanib 125 mg QD	SD	296	1	346	0	17.4	35.3
pt947	Placebo	SD	43	0	439	0		
pt948	Motesanib 125 mg QD	SD	93	1	380	1	22	51.2
pt949	Placebo	PD	92	1	103	1	29.4	
pt950	Placebo	PD	58	1	105	1	30.2	35.2
pt951	Placebo	SD	102	1	102	1	37.3	
pt952	Placebo	ND	91	1	91	1		
pt953	Motesanib 125 mg QD	PR	163	0	831	0	22.7	
pt954	Motesanib 125 mg QD	SD	102	1	201	1	37.4	225.1
pt955	Placebo	SD	292	1	537	1	23.8	24
pt956	Placebo	SD	290	1	441	1	24.9	26.4
pt957	Motesanib 125 mg QD	SD	173	1	354	1	15.7	43.2
pt958	Placebo	PR	121	1	204	1	17.9	19.2
pt959	Motesanib 125 mg QD	SD	83	1	127	1	24.5	69.6
pt960	Placebo	PR	163	1	454	1	20.6	25.4
pt961	Motesanib 125 mg QD	PR	378	1	533	0	22.7	25.9
pt962	Placebo	SD	168	1	201	1	16.7	23.6
pt963	Motesanib 125 mg QD	UE	133	0	300	1	20.5	
pt964	Motesanib 125 mg QD	PR	128	1	412	1	25.9	83.4
pt965	Placebo	SD	118	1	118	1	32.9	28.7
pt966	Motesanib 125 mg QD	ND	34	1	34	1	32.6	79.8

Patient ID	Treatment Arm	Best response per RECIST	PFS, days	PFS actual flag	OS, days	OS actual flag	Baseline PLGF, pg/mL	Week 4 PLGF, pg/mL
pt967	Motesanib 125 mg QD	PR	125	1	264	1	27.9	38.6
pt968	Placebo	PR	213	1	303	1	18.9	23.4
pt969	Placebo	SD	99	1	99	1	58.4	40
pt970	Placebo	SD	429	1	457	0	26.3	22.4
pt971	Motesanib 125 mg QD	SD	92	1	203	1	16	22.6
pt972	Placebo	PD	43	1	164	1	26.1	25.9
pt973	Placebo	PR	188	1	220	1	24.4	27.5
pt974	Placebo	PD	15	1	253	1		
pt975	Placebo	SD	90	1	189	1	19	20.8
pt976	Placebo	PR	186	1	345	0	27.4	23.8
pt977	Motesanib 125 mg QD	PR	86	0	195	1	21.5	35.5
pt978	Motesanib 125 mg QD	PR	94	0	338	0	22	196.3
pt979	Motesanib 125 mg QD	PR	242	1	242	1	38.2	67.7
pt980	Motesanib 125 mg QD	PR	247	1	514	1	143.7	28.8
pt981	Placebo	PR	177	1	893	0	30	17.5
pt982	Motesanib 125 mg QD	SD	254	1	399	1	32.9	58.9
pt983	Motesanib 125 mg QD	PR	344	0	851	0	24.6	77.1
pt984	Motesanib 125 mg QD	PR	297	1	970	0	26.2	19.3
pt985	Placebo	PR	255	1	969	0	30	27.6
pt986	Motesanib 125 mg QD	SD	169	1	484	1	21.7	61.5
pt987	Motesanib 125 mg QD	SD	87	1	125	1	22.9	31.3
pt988	Placebo	PD	51	1	94	1	20.1	25.1
pt989	Motesanib 125 mg QD	SD	216	1	904	0	17.1	29.7
pt990	Motesanib 125 mg QD	PR	89	0	196	1	25	46.4
pt991	Placebo	SD	345	1	575	1	18.2	22.2
pt992	Placebo	PR	212	1	863	0	27.6	29.2
pt993	Placebo	SD	46	0	338	1	20.2	23.3
pt994	Motesanib 125 mg QD	PR	338	1	561	0	20.4	85.8
pt995	Placebo	SD	246	1	563	0	15.7	16.7
pt996	Motesanib 125 mg QD	SD	62	1	335	1	22.4	51
pt997	Placebo	PR	380	1	498	0	21.5	23.5
pt998	Placebo	SD	88	1	480	1	19.4	23.9
pt999	Motesanib 125 mg QD	PR	215	1	483	0	19.5	50.8
pt1000	Motesanib 125 mg QD	SD	89	1	414	0	18.3	35.6
pt1001	Motesanib 125 mg QD	SD	293	1	407	0	19.2	53.2
pt1002	Motesanib 125 mg QD	PR	170	0	320	1	20.2	106.5
pt1003	Placebo	PR	127	1	229	1	20.3	23.2
pt1004	Placebo	PR	294	1	897	0	23.3	28.1
pt1005	Motesanib 125 mg QD	SD	88	1	220	1		
pt1006	Motesanib 125 mg QD	PR	127	0	309	1	29.7	48.4
pt1007	Placebo	SD	120	1	379	0	17.6	23.5
pt1008	Motesanib 125 mg QD	SD	82	1	182	1	18.9	35.3

Patient ID	Treatment Arm	Best response per RECIST	PFS, days	PFS actual flag	OS, days	OS actual flag	Baseline PLGF, pg/mL	Week 4 PLGF, pg/mL
pt1009	Placebo	PR	193	1	783	1	22.7	25.4
pt1010	Placebo	SD	176	1	393	1	23.4	24.8
pt1011	Motesanib 125 mg QD	SD	41	0	931	0	18.9	
pt1012	Placebo	PD	39	1	474	1	23.5	23.6
pt1013	Placebo	ND	1	0	562	0		
pt1014	Placebo	PR	218	1	556	0	18.8	27
pt1015	Motesanib 125 mg QD	PR	210	1	439	1	25.3	56.6
pt1016	Motesanib 125 mg QD	SD	255	1	487	0	13.7	20
pt1017	Motesanib 125 mg QD	PR	213	1	814	1	26.5	43.7
pt1018	Motesanib 125 mg QD	PR	204	1	506	1	22.5	66.6
pt1019	Motesanib 125 mg QD	SD	295	1	998	0	30.4	42.9
pt1020	Motesanib 125 mg QD	PR	893	0	921	0	24.7	50.9
pt1021	Motesanib 125 mg QD	PR	176	1	593	1	26.1	40.7
pt1022	Placebo	PD	42	1	85	1	22.8	27.5
pt1023	Placebo	PR	378	1	906	0	21.8	26.7
pt1024	Placebo	SD	213	1	262	1	26.7	26.4
pt1025	Placebo	SD	211	1	692	1	32.1	34.3
pt1026	Placebo	SD	122	1	323	1	25.1	25.3
pt1027	Motesanib 125 mg QD	PR	504	1	871	0	30	241
pt1028	Motesanib 125 mg QD	PR	170	0	522	0	26.6	123.5
pt1029	Placebo	PR	335	1	386	0	14.5	17.3
pt1030	Motesanib 125 mg QD	SD	41	0	65	0	24.6	60.2
pt1031	Motesanib 125 mg QD	CR	76	0	486	1	21.2	74.1
pt1032	Placebo	SD	162	0	586	1	31.9	30.5
pt1033	Motesanib 125 mg QD	SD	171	0	865	0	27.5	22.8
pt1034	Placebo	PR	127	1	431	0	21.9	26.4
pt1035	Placebo	SD	186	0	329	1	38.9	38
pt1036	Placebo	SD	158	0	354	0	20.6	30.8
pt1037	Motesanib 125 mg QD	SD	210	1	210	1	27	166.8
pt1038	Placebo	SD	128	1	336	1	26.3	20.2
pt1039	Motesanib 125 mg QD	PR	948	0	976	0	22.1	29.5
pt1040	Motesanib 125 mg QD	ND	1	0	263	1	24.8	
pt1041	Placebo	PR	211	1	953	0	18.8	22.6
pt1042	Motesanib 125 mg QD	PR	168	1	415	1	28.4	68.8
pt1043	Placebo	PR	256	0	886	0	26	27.2
pt1044	Placebo	SD	88	1	203	1	30.8	31.3
pt1045	Placebo	SD	167	1	548	0	26	23.1
pt1046	Motesanib 125 mg QD	PR	212	1	290	1	17.2	56.1
pt1047	Motesanib 125 mg QD	PR	213	1	381	0	17.7	42.6
pt1048	Motesanib 125 mg QD	PR	123	1	227	1	22.8	129.1
pt1049	Placebo	SD	85	1	628	1	25.2	30.8
pt1050	Motesanib 125 mg QD	PR	211	0	981	0	21.5	162.3

Patient ID	Treatment Arm	Best response per RECIST	PFS, days	PFS actual flag	OS, days	OS actual flag	Baseline PLGF, pg/mL	Week 4 PLGF, pg/mL
pt1051	Placebo	SD	97	0	835	1	20.5	19.1
pt1052	Placebo	SD	40	0	828	1	22.3	
pt1053	Motesanib 125 mg QD	PR	217	1	911	0	19.5	91.7
pt1054	Placebo	SD	83	1	232	1	21.3	22.5
pt1055	Motesanib 125 mg QD	PR	256	0	462	0	23.7	34.2
pt1056	Motesanib 125 mg QD	SD	113	0	450	0	17.3	76.7
pt1057	Placebo	PR	246	1	375	1	22.5	25.1
pt1058	Motesanib 125 mg QD	PR	268	1	752	1	24.6	47.7
pt1059	Placebo	SD	136	1	311	1	23.7	26.3
pt1060	Motesanib 125 mg QD	PR	222	0	914	1	21.6	30.1
pt1061	Motesanib 125 mg QD	SD	86	1	893	0	22.2	51.7
pt1062	Placebo	PD	43	1	410	1	28.4	28.9
pt1063	Motesanib 125 mg QD	PR	93	0	636	1	28.6	97.9
pt1064	Placebo	SD	87	1	247	1	24.6	27.9
pt1065	Motesanib 125 mg QD	PR	172	1	445	0	39.9	74
pt1066	Motesanib 125 mg QD	PR	385	0	406	0	24.9	37.8
pt1067	Motesanib 125 mg QD	PR	243	1	368	0	20.4	35.4
pt1068	Placebo	PR	343	1	1008	0	16.4	17.7
pt1069	Motesanib 125 mg QD	PR	336	1	973	0	66.4	32.7
pt1070	Motesanib 125 mg QD	PR	296	1	382	1	29.4	93.1
pt1071	Placebo	PR	256	1	484	1	17.3	20.8
pt1072	Placebo	PR	173	1	952	0	18.1	18.9
pt1073	Placebo	PR	172	1	246	1	16.5	21
pt1074	Motesanib 125 mg QD	PR	410	1	941	0	19.6	34.4
pt1075	Placebo	SD	131	0	925	0	20.2	23.4
pt1076	Motesanib 125 mg QD	PR	126	1	294	1	31.7	160.5
pt1077	Placebo	SD	214	1	882	0	29	30.8
pt1078	Motesanib 125 mg QD	PR	158	1	871	0	16.2	55.9
pt1079	Motesanib 125 mg QD	PR	470	1	854	0	19.6	32.9
pt1080	Placebo	PR	172	1	631	1	26.2	23.5
pt1081	Placebo	SD	80	1	432	1	24.7	19.9
pt1082	Motesanib 125 mg QD	PR	162	1	581	0	25.1	35.6
pt1083	Motesanib 125 mg QD	SD	173	1	497	1	28.6	139.9
pt1084	Placebo	UE	27	0	569	0	22.4	
pt1085	Motesanib 125 mg QD	PR	287	1	555	0	19	38.3
pt1086	Motesanib 125 mg QD	PR	215	1	543	0	23.6	109
pt1087	Motesanib 125 mg QD	PD	60	1	198	1	37.6	
pt1088	Placebo	SD	41	0	518	0	23.9	25.8
pt1089	Placebo	SD	130	1	220	1	22.6	28.4
pt1090	Placebo	SD	64	1	117	1	26.7	26.4
