# Supplementary material for: Challenges in Developing a Validated Biomarker for Angiogenesis Inhibitors: The Motesanib Experience
Source: PLoS One. 2014 Oct 14;9(10):e108048. doi: 10.1371/journal.pone.0108048 (PMC4196848; doi:10.1371/journal.pone.0108048)
Supplement: Protocol S1 — Phase 3 study protocol. (PDF) [file pone.0108048.s005.pdf]

Title: A Phase 3, Multicenter, Randomized, Placebo-Controlled, Double-Blind Trial of AMG 706 in Combination With Paclitaxel and Carboplatin for Advanced Non-small Cell Lung Cancer

REDACTED

Study Design and Treatment Schema

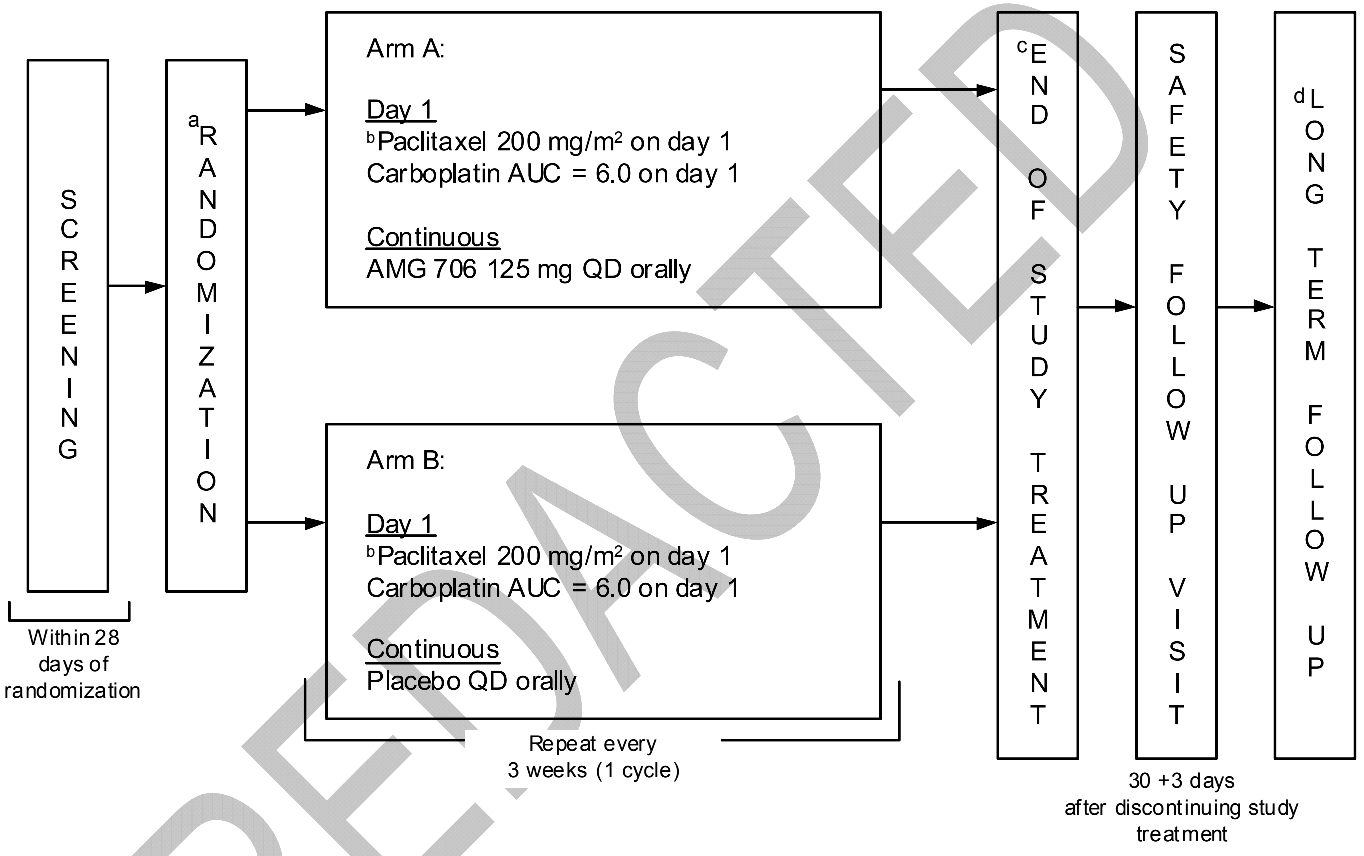

REDACTED

[a] Subjects will be randomized in a 1:1 ratio to Arm A or Arm B.
[b] A maximum of 6 cycles of paclitaxel-carboplatin regimen will be administered. AMG 706 or placebo may continue until disease progression (per modified RECIST) or intolerability.
[c] End of study treatment due to disease progression, drug intolerability or withdrawal of consent, or death.
[d] Every 3 months ± 2 weeks following each subjects' last follow up visit up to 36 months after the last subject on the study was randomized

6. OBJECTIVES

6.1 Primary Objective

To determine if treatment with AMG 706 in combination with paclitaxel and carboplatin improves overall survival (OS) compared to treatment with placebo in combination with paclitaxel and carboplatin in subjects with advanced non-squamous NSCLC and in subjects with adenocarcinoma histology (adenocarcinoma subpopulation).

6.2 Secondary Objectives

- To evaluate progression-free-survival time, objective tumor response rate (only in subjects with measurable disease) and duration of response in subjects with non-squamous NSCLC histology and in subjects with adenocarcinoma histology
- To evaluate the association of AMG 706 treatment-induced PlGF increase with OS in subjects with non-squamous NSCLC and in subjects with adenocarcinoma histology
- To evaluate the safety and tolerability of AMG 706 in combination with paclitaxel and carboplatin compared to placebo in combination with paclitaxel and carboplatin in subjects with non-squamous NSCLC histology and adenocarcinoma histology
- To evaluate OS, PFS, AMG 706 treatment-induced PlGF increase association with OS, ORR (only in subjects with measurable disease) and duration of response in subjects with non-squamous, non-adenocarcinoma histology
- To evaluate the pharmacokinetics of AMG 706 and metabolites when administered with paclitaxel and carboplatin (in approximately 250 subjects at selected centers)
- To evaluate the pharmacokinetics (C_{max}) of carboplatin (total and unbound platinum concentrations) when administered in combination with paclitaxel and AMG 706 or placebo (in approximately 20 to 30 subjects at selected centers outside of the European Union)

6.3 Exploratory Objectives

- To investigate **other** potential biomarker development based on assessment of blood cells, tumor cells and the proposed mechanism of action of AMG 706 and response
- To investigate the effects of genetic variation in drug metabolism genes, cancer associated genes and drug target genes on subject response to AMG 706 in combination with paclitaxel and carboplatin (optional with separate informed consent)
- To investigate patient reported outcomes (PRO) using EQ-5D and the FACT-L questionnaires.
- To investigate OS, PFS, ORR (only in subjects with measurable disease) and duration of response in subjects with squamous cell histology who were randomized prior to the Protocol Amendment 2

4. SUBJECT ELIGIBILITY

Investigators will be expected to maintain a screening log of all potential study candidates that include limited information about the potential candidate (age, sex and

REDACTED

ethnicity), date and outcome of the screening process (eg, enrolled into study, reason for ineligibility, or refused to participate). All subjects screened for participation will need to sign an approved informed consent form before any study specific procedures are performed.

4.4 Inclusion Criteria

4.1.1 Disease related

- Histologically confirmed (cytological specimens obtained by bronchial washing or brushing, or fine-needle aspiration are acceptable), unresectable stage IIIB with pericardial or pleural effusion or stage IV or recurrent non-squamous NSCLC. Evaluation of effusions (ie, with cytology) is not required if diagnosis of non-squamous NSCLC has been otherwise histologically confirmed.
- Measurable or non-measurable disease per modified RECIST criteria.
- ECOG performance status of 0 or 1.
- Life expectancy of ≥ 3 months as documented by the investigator.

4.1.2 Demographic

- Men or women aged ≥ 18 years old.

4.1.3 Laboratory

- Hematological function, as follows:
  - Absolute neutrophil count (ANC) ≥ 1.5 x 10^9/L
  - Platelet count ≥ 100 x 10^9/L and ≤ 850 x 10^9/L
  - Hemoglobin ≥ 9 g/dL
- Renal function, as follows:
  - Creatinine clearance (GFR) > 40 mL/min (calculated by Cockcroft-Gault formula, see Section 6.2.2.2).
  - Urinary protein quantitative value of ≤ 30 mg in urinalysis or ≤ 1+ on dipstick unless total quantitative protein is < 500 mg in a 24-hour urine sample.
- Hepatic function, as follows:
  - Aspartate aminotransferase (AST) ≤ 2.5 x upper limit of normal ULN OR AST < 5 x ULN if liver metastases are present.
  - Alanine aminotransferase (ALT) ≤ 2.5 x ULN OR ALT < 5 x ULN if liver metastases are present.
  - Alkaline phosphatase ≤ 2.0 x ULN OR alkaline phosphatase < 5 x ULN if liver or bone metastases are present.
  - Total bilirubin < 1.5 x ULN OR if total bilirubin < 3 X ULN if subject has UGT1A1 promoter polymorphism (ie, Gilbert syndrome) confirmed by genotyping or Invader® UGT1A1 Molecular Assay prior to randomization.
  - Partial thromboplastin (PTT) or activated partial thromboplastin (aPTT) ≤ 1 x ULN and international normalized ratio (INR) ≤ 1.5 x ULN.

4.1.4 Ethical

- Competency to give written informed consent.

4.1.5 General

- Ability to take oral medications.
- Able to start protocol directed therapy within 7 days from date of randomization.

4.5 Exclusion Criteria

4.2.1 Disease Related

- Subjects with adenosquamous histology or an unclear histology subtype (eg, not otherwise specified) containing greater than 10% squamous cells
- Untreated or symptomatic central nervous system metastases. Subjects with a history of brain metastases are eligible if definitive therapy has been administered (surgery and/or radiation therapy), there is no planned treatment for brain metastasis, and the subject is clinically stable and is off corticosteroids for at least 2 weeks prior to randomization.
- Prior chemotherapy as follows:
  - Any prior chemotherapy for advanced non-squamous NSCLC
  - Any prior adjuvant chemotherapy for non-squamous NSCLC within 52 weeks prior to randomization. Adjuvant chemotherapy completed > 52 weeks prior to randomization is permitted
  - Any prior chemoradiation for locally advanced stage III disease
- Central (chest) radiation therapy within 28 days prior to randomization, radiation therapy within 14 days prior to randomization for peripheral lesions.
- History of pulmonary hemorrhage or gross hemoptysis (approximately 3 mL of bright red blood or more) within 6 months prior to randomization.

4.2.2 Medications

- Prior targeted therapies, including but not limited to:
  - AMG 706, inhibitors of VEGF (eg, SU5416, SU6668, ZD6474, SU11248, PTK787, AZD2171, AEE-788, sorafenib, bevacizumab), or EGFr (eg, panitumumab**,** cetuximab, gefitinib, erlotinib)
- Any anticoagulation therapy within 7 days prior to randomization. The use of low-dose warfarin [≤ 2 mg daily] or low molecular weight heparin or heparin flushes for prophylaxis against central venous catheter thrombosis is allowed.
- Known history of allergy or hypersensitivity reaction to paclitaxel or carboplatin.

4.2.3 General

- Prior (within 30 days of randomization) yellow fever vaccination
- History of arterial or venous thrombosis within 12 months prior to randomization.
- History of bleeding diathesis or bleeding within 14 days prior to randomization.
- Peripheral neuropathy > grade 1 per Common Terminology Criteria for Adverse Events (CTCAE) Version 3.0.
- Clinically significant cardiac disease within 12 months of randomization, including myocardial infarction, unstable angina, grade 2 or greater peripheral vascular disease, cerebrovascular accident, transient ischemic attack, percutaneous

REDACTED

transluminal coronary angioplasty/stent, congestive heart failure or ongoing arrhythmias requiring medication.

- Any kind of disorder that compromises the ability to comply with the study procedures.
- Open wound, ulcer or fracture.
- Active infection requiring systemic treatment or any uncontrolled systemic infection ≤ 14 days prior to randomization.
- Uncontrolled hypertension as defined by resting blood pressure > 150/90 mm Hg. Anti-hypertensive medications are allowed if the subject is stable on their current dose at the time of randomization.
- History of other primary cancer unless:
  - Curatively resected non-melanomatous skin cancer
  - Curatively treated cervical carcinoma in situ
  - Other primary solid tumor curatively treated with no known active disease present and no curative treatment administered for the last 3 years
- Surgery:
  - Major surgical procedures within 28 days prior to randomization.
  - Minor surgical procedures within 14 days prior to randomization.
  - Failure to recover from prior surgery.
  - Placement of a central venous access device (including ports and tunneled or non- tunneled catheters) within 7 days prior to randomization.
  - Planned elective surgery while on study treatment.
  - Core needle biopsy within 7 days prior to randomization.
- Not recovered from all previous therapies (ie, radiation, surgery and medications). Adverse events related to previous therapies must be CTCAE grade ≤ 1 at screening or returned to the subject's baseline prior to their most recent previous therapy.
- Participation in therapeutic clinical trials or currently receiving other investigational treatment(s) within 30 days prior to randomization.
- Pregnant (eg, positive HCG test-urine or serum) or breast feeding woman.
- Any subject not consenting to use adequate contraceptive precautions (eg, hormonal, barrier or abstinence) during the course of the study and for 6 months after the last treatment.
- Known to be human immunodeficiency virus (HIV), hepatitis B surface antigen or hepatitis C positive.
- Known chronic hepatitis.
- History of any medical or psychiatric condition or laboratory abnormality that in the opinion of the investigator may increase the risks associated with the study participation or investigational product(s) administration or may interfere with the interpretation of the results.
- Previously randomized to this study.
- Not available for follow-up assessments or unable to comply with study requirements.

REDACTED

5. SUBJECT ENROLLMENT

5.1 Randomization

The randomization will be stratified by stage (stage IIIB vs. stage IV or recurrent), weight loss (< 5% vs. ≥ 5% in the previous 6 months prior to randomization), sex (male vs. female) and prior adjuvant chemotherapy (yes vs. no).

A subject will be assigned by IVRS to one of the 2 treatment arms (AMG 706 in combination with paclitaxel and carboplatin [Arm A], or placebo in combination with paclitaxel and carboplatin [Arm B]), in a 1:1 ratio, with the assignment balanced within strata at the time of study entry.

The randomization list will be generated using random permuted blocks. The list will be produced and maintained by an Amgen representative not involved in the conduct of the study.

6. TREATMENT PROCEDURES

AMG 706 and placebo will be considered as the only investigational products and will be provided by Amgen. The IVRS must be called to confirm the bottle numbers that must be dispensed to the subject.

Throughout the study, a cycle is defined as a 3-week period between 2 chemotherapy infusions plus time to recover from any toxicity. If a subject continues in the monotherapy phase, visits will occur every 3 weeks.

6.1 Investigational Products

6.1.1 AMG 706 and Placebo Dosage, Administration, and Schedule

AMG 706 and placebo will be available as 25 mg tablets. The dose of AMG 706 or placebo for this study is 125 mg orally self-administered once daily starting on day 1 of cycle 1. Subjects must take 125 mg AMG 706 or placebo (5 x 25 mg tablets). AMG 706 or placebo should be taken first thing in the morning, on an empty stomach. Subjects should refrain from food or liquids, except water, for 2 hours before and 1 hour after, unless otherwise indicated. Subjects will be asked to refrain from taking AMG 706 or placebo until after clinical assessments (such as PRO, physical examination, laboratory tests, BP) occur on days the subject returns to clinic and before chemotherapy administration.

On day 1 of cycle 3, 5 and day 1 of every third visit, selected subjects will be requested to delay administration of AMG 706 or placebo until after pharmacokinetic samples are

REDACTED

taken. Once samples have been collected, AMG 706 or placebo will be taken at the study center, prior to administration of chemotherapy.

In the event chemotherapy is delayed for reasons other than AMG 706-related toxicity, AMG 706 or placebo will continue to be taken.

Subjects will be asked to record the date, time, and number of tablets consumed in a subject drug diary.

Missed AMG 706 or placebo doses will not be made up. The next dose must be taken as scheduled.

Subjects must be instructed to immediately report any unscheduled interruption of AMG 706 or placebo due to toxicity to the investigator.

AMG 706 or placebo will be administered until subjects either develop disease progression, are unable to tolerate AMG 706/placebo, withdrawal, or death.

The effects of overdose of AMG 706 are not known.

6.1.2 AMG 706 or Placebo Toxicity Management

For the purposes of AMG 706 toxicity management, any reference to AMG 706 will also assume AMG 706 or placebo. All the toxicity grades are based on CTCAE Version 3.0.

AMG 706 may be administered as monotherapy after completion of a maximum of 6 cycles of chemotherapy at the discretion of the investigator until disease progression or the development of unacceptable toxicity.

Any toxicity necessitating the withholding of AMG 706 for > 3 weeks will result in permanent discontinuation of AMG 706. If further treatment with on-study chemotherapy is deemed to be clinically appropriate by the investigator, subjects may continue to receive the on-study chemotherapy up to a maximum of 6 cycles unless disease progression or chemotherapy intolerability is observed.

Subjects who discontinue all treatment will be required to complete a safety follow-up visit and will enter the long-term follow-up.

The management of specific AMG 706 related toxicities will occur as follows.

6.1.2.1 Hypertension

Treatment with anti-VEGF agents has been associated with increases in systolic and diastolic blood pressure. The risk of hypertension may be greater for subjects with a pre-existing history of this condition. Subjects with a history of hypertension and/or

REDACTED

receiving antihypertensive medication with a blood pressure reading of ≤ 150/90 mm Hg will have CTCAE grade 0 hypertension at study baseline.

Blood pressure will be monitored weekly for the first 6 weeks and then at each clinic visit thereafter. If deemed necessary by the investigator, a more frequent blood pressure monitoring may be performed, particularly for subjects requiring initiation or adjustment of anti-hypertensive medication(s).

AMG 706 dosing and dose modifications must be carried out according to the guidelines found in Table 1:

REDACTED

Table 1. Dose Modifications for AMG 706 Hypertensive Events

Hypertension (HTN)	Anti-hypertensive Therapy	AMG 706 Treatment	AMG 706 Treatment Resumed When	AMG 706 Resumed Dose
Asymptomatic HTN (eg, >150/90 or diastolic increase of > 20 mm Hg from baseline) not requiring immediate or urgent management, as determined by the investigator	Initiate or optimize therapy as determined by the investigator	Continue at current dose without interruption as determined by the investigator	N/A	No Change
Hypertension (eg, symptomatic or diastolic > 110 mm Hg) that requires immediate or urgent management as determined by the investigator	Initiate or optimize therapy as determined by the investigator	Withhold	Diastolic blood pressure returns to within 20 mm Hg of baseline measurement taken on study day 1 before the first dose of AMG 706 AND to a clinically acceptable range, BP ≤ 150/90 mm Hg for the subject while on an anti-hypertensive therapy	Reduce 25 mg per dose
Reoccurrence of HTN that requires immediate or urgent management as determined by the investigator	As determined by the investigator	Withhold	Diastolic blood pressure returns to within 20 mm Hg of baseline measurement taken on study day 1 before the first dose of AMG 706 AND to a clinically acceptable range, BP ≤ 150/90 mm Hg for the subject while on an anti-hypertensive therapy	Reduce 25 mg per current dose
Reoccurrence of HTN that requires immediate or urgent management as determined by the investigator despite 2 dose reductions (total reduction of 50 mg per dose).	As determined by the investigator	Discontinue	N/A	N/A
If treatment with AMG 706 is withheld for > 3 continuous weeks due to persistent severe HTN	As determined by the investigator	Discontinue (Subject removed from treatment phase)	N/A	N/A

N/A: not applicable

REDACTED

6.1.2.2 Thrombosis

AMG 706 must be withheld for at least 1 week for subjects who develop grade 3 or asymptomatic grade 4 venous thrombosis (eg, incidental pulmonary embolism diagnosed radiographically). Subjects must be treated with anti-coagulation therapy.

If warfarin is used, an INR > 2 on a stable dose must be observed prior to restarting treatment with AMG 706. After withholding AMG 706 for at least 1 week and receiving anti-coagulation therapy, subjects may resume AMG 706 treatment at the same dose and must continue to receive anti-coagulation therapy according to the investigator's discretion.

Due to potential interaction between AMG 706 or placebo and warfarin, investigators are advised to monitor coagulation parameters (INR) as follows:

- twice weekly for the first week of warfarin therapy
- once weekly for the subsequent 4 weeks of warfarin therapy or until a stable INR is achieved
- twice weekly upon discontinuation of AMG 706 or placebo until INR is stable.

More frequent measurements of coagulation parameters may be required.

The development of symptomatic grade 4 venous thrombosis or grade 3 or 4 arterial thrombosis will result in the permanent discontinuation of AMG 706.

In the event of discontinuation of AMG 706, if further treatment with chemotherapy is deemed to be clinically appropriate by the investigator, subjects may continue to receive the chemotherapy up to a maximum of 6 cycles unless disease progression or chemotherapy intolerability is observed.

Subjects who discontinue all treatment will be required to complete a safety follow-up visit and will enter long term follow up.

REDACTED

6.1.2.3 Proteinuria

A dipstick urinalysis is required prior to each 3-week cycle of treatment. Trace proteinuria must not be considered a positive result unless confirmed in a repeat dipstick. After confirmed documentation of significant proteinuria (> 30 mg or > 1 + by urine dipstick), subjects must undergo additional evaluation including:

- 24-hour urine collection for total protein and calculated creatinine clearance (GFR) by Cockcroft-Gault formula (see Section 6.2.2.2)
- microscopic examination of fresh urine

If the 24-hour urine collection confirms proteinuria ≤ 2000 mg within 24 hours, the subject can continue AMG 706 treatment as scheduled. A 24-hour urine collection for total protein must be performed prior to each subsequent cycle of treatment to monitor the degree of proteinuria until it has decreased to < 500 mg/24 hours.

Subjects who develop > 2000 mg proteinuria within 24 hours must not receive any additional doses of AMG 706 until the proteinuria improves to ≤ 2000 mg within 24 hours. Subjects can then resume treatment at the same dose and schedule of AMG 706.

For those subjects that develop > 2000 mg proteinuria within 24 hours, a 24-hour urine collection for total protein must be performed prior to each subsequent 3-week cycle of treatment to monitor the degree of proteinuria.

6.1.2.4 Bleeding

Bleeding will be graded according to the hemorrhage category of CTCAE Version 3.0.

- Grade 1: hold AMG 706 until bleeding resolves to grade 0. Once resolved, resume treatment with no dose modification.
- Grade ≥ 2: discontinue AMG 706 permanently

6.1.2.5 Hemoptysis

Hemoptysis will also be graded according to the hemorrhage category of CTCAE Version 3.0

- Grade 1: subjects must be evaluated to determine the source of hemoptysis. If no source is found and this resolves within 1 week, treatment with AMG 706 must continue as scheduled. There will be no AMG 706 dose reductions
- Grade ≥ 2: discontinue AMG 706

6.1.2.6 Cholecystitis / Cholelithiasis

AMG 706 must be held in subjects who develop the clinical diagnosis of cholecystitis. Subjects should be managed per routine institutional practice.

If a subject has an ultrasound scan of the gallbladder at any time during the study, the following must be provided in the case report form, if available: gallbladder measurements (length and width, cm), presence of pericholecystic fluid (yes or no), gallbladder wall thickening (size in mm), common bile duct size (in mm), and presence of stones (yes or no).

If a subject undergoes cholecystectomy while on study, AMG 706 may be restarted without dose reduction (ie, at the dose prior to surgery) after adequate wound healing

REDACTED

and approval from the Amgen Medical Monitor. For subjects who have undergone cholecystectomy and have signed the optional section of the informed consent for tissue collection, sites are strongly encouraged to submit pathology slides, tissue samples, and bile samples for safety-related analysis (see Study Laboratory Manual).

Subjects managed without cholecystectomy must permanently discontinue AMG 706.

Subjects found to have incidental cholelithiasis, eg on imaging studies, without cholecystitis or symptoms attributed to gallbladder disease should continue the current dose of AMG 706.

6.1.2.7 Gallbladder Enlargement

Subjects with asymptomatic enlargement of the gallbladder detected radiographically should continue the current dose of AMG 706.

If a subject has an ultrasound of the gallbladder at any time during the study, the following must be provided in the case report form, if available: gallbladder measurements (length, and width, cm), presence of pericholecystic fluid (yes or no), gallbladder wall thickening (size in mm), common bile duct size (in mm), and presence of stones (yes or no).

Subjects who develop symptoms (eg, abdominal pain, nausea, vomiting) attributed by the investigator to gallbladder enlargement in the absence of cholecystitis must have their AMG 706 held. Once the symptoms resolve to ≤ Grade 1 (as defined by CTCAE version 3.0) or return to the subject's baseline, AMG 706 may be resumed with one dose level reduction. If symptomatic gallbladder enlargement recurs after a single dose reduction for gallbladder toxicity, AMG 706 must be permanently discontinued.

If a subject undergoes cholecystectomy while on study, AMG 706 may be restarted without dose reduction (ie, at the dose prior to surgery) after adequate wound healing and approval from the Amgen Medical Monitor. For subjects who have undergone cholecystectomy and have signed the optional section of the informed consent for tissue collection, sites are strongly encouraged to submit pathology slides, tissue samples, and bile samples for safety-related analysis (see Study Laboratory Manual).

6.1.2.8 Hypothyroidism

Tyrosine kinase inhibitors (eg, sunitinib, imatinib) have been reported to induce hypothyroidism both in subjects with baseline normal thyroid function and in subjects receiving thyroid hormone replacement therapy. The adverse events of hypothyroidism and/or elevated thyroid stimulating hormone (TSH) have been observed with an

REDACTED

incidence of approximately 12% across AMG 706 monotherapy studies. Currently, TSH and thyroxine (free T4) levels are being monitored as a routine laboratory value for all subjects receiving AMG 706. If subjects develop biochemical hypothyroidism (ie, elevated TSH levels above upper limits of normal and/or a low T4 level) and/or signs/symptoms of hypothyroidism, consider thyroid hormone replacement therapy (ie, levothyroxine) according to institutional guidelines.

6.1.2.9 Hepatotoxicity

Increased liver function tests (LFTs) may occur in subjects on the study. AMG 706 should be withheld in those subjects who experience grade 3 level elevation in their LFTs. AMG 706 can be resumed after LFTs decrease to grade 1 or 0 without a dose reduction. If a subject experiences a grade 4 level LFT elevation or a second grade 3 LFT level elevation, AMG 706 should be permanently discontinued.

6.1.2.10 Management of Other Toxicities

When a subject experiences a grade 3 or 4 toxicity considered to be related to AMG 706, or the combination of AMG 706 with paclitaxel and carboplatin, AMG 706 will be withheld until the toxicity resolves to grade 1 (as defined by CTCAE version 3.0) or returns to the subject's baseline. AMG 706 will then be reduced in dose by 25 mg or discontinued if the dose has already been reduced twice. Any dose reduction will be permanent.

For any subject experiencing grade 3 or 4 toxicity that persists for > 3 weeks or recurs after a dose delay and/or reduction, AMG 706 will be discontinued permanently.

6.2 Chemotherapy (Paclitaxel and Carboplatin)

All subjects should be treated with 200 mg/m^2 paclitaxel, given over 3 hours ± 30 minutes on day 1 of each treatment cycle (± 3 days), immediately followed on the same day by carboplatin at a dose calculated to produce a target AUC of 6.0 mg/mL x min given over 30 ± 10 minutes IV infusion.

The dosing schedules are described below:

Before administration of chemotherapy, the subject must meet the following parameters based on laboratory and clinical assessments performed within 72 hours prior to infusions:

REDACTED

- ANC ≥ 1.5 x 10^9/L
- Platelet count ≥ 100 x 10^9/L
- Bilirubin < 1.5 x ULN (< 3 x ULN for subjects with UGT1A1 promoter polymorphism)
- AST and ALT ≤ 2.5 x ULN (if liver metastases ≤ 5 x ULN)
- Calculated creatinine clearance (GFR) > 40 mL/min by Cockcroft-Gault formula (see Section 6.2.2.2)
- No peripheral neuropathy > grade 1
- No grade 3 myalgias/arthralgias refractory to non steroidal anti-inflammatory drugs (NSAIDs)
- No symptoms or signs of active infection

If parameters are not met by the scheduled chemotherapy dosing date, blood tests and/or clinical evaluations must occur at least weekly to monitor these parameters. The guideline in Section 6.2.3 will be followed for chemotherapy delay and dosing resumption.

Carboplatin and paclitaxel should be obtained by each site from commercial sources. Both carboplatin and paclitaxel are cytotoxic compounds and study personnel will follow the institutional standard operating procedures (SOPs) for handling cytotoxic compounds.

For additional details and full prescribing information, please see the most recent version of the respective package inserts.

6.2.1 Paclitaxel

6.2.1.1 Paclitaxel Premedication

All subjects should be premedicated before paclitaxel administration using the guidelines below or according to the investigator's discretion:

- Dexamethasone 20 mg orally approximately 12 ± 1 hour and 6 ± 1 hour before paclitaxel infusion. The investigator or designee should verify and document that the subject took this appropriately.
- Diphenhydramine (or its equivalent) 50 mg IV or orally 30 to 60 minutes before paclitaxel infusion
- Ranitidine 50 mg IV or orally 30 to 60 minutes before paclitaxel infusion. AMG 706 should be taken at least 60 minutes before ranitidine. Diphenhydramine and ranitidine may be given concurrently and as either oral or IV medications, with IV being the preferred route of administration.

Additional premedications may be used when clinically indicated at the discretion of the investigator.

All premedications must be recorded in the eCRF.

REDACTED

6.2.1.2 Paclitaxel Dosage, Administration and Schedule

Paclitaxel 200 mg/m^2 should be administered by IV infusion over 3 hours ± 30 minutes through polyethylene-line administered sets once every 3 weeks ± 3 days. The investigator will determine the frequency of vital sign monitoring during the infusion.

If a subject's actual body weight is more than twice the ideal body weight, the paclitaxel dose may be modified at the discretion of the investigator per local guidelines.

If body weight changes during therapy by > 5 kg, the paclitaxel dose should be re-calculated for the next cycle.

Paclitaxel will be prepared according to the most current package insert.

6.2.1.3 Paclitaxel Safety Profile

The most common adverse events associated with paclitaxel (occurring in > 50% of subjects receiving single agent paclitaxel at different doses and using various dosing schedules) are myelosuppresion, reversible alopecia, peripheral neuropathy, myalgia/arthralgia, and nausea/vomiting. Other less commonly reported adverse events include hypersensitivity reactions, infections, bleeding, diarrhea, mucositis, liver function test elevations, injection site reactions, and cardiovascular effects that have included hypotension, bradycardia, hypertension, arrhythmias and other ECG abnormalities, syncope, and venous thrombosis. Anaphylaxis and severe hypersensitivity reactions have occurred in 2% to 4% of subjects receiving paclitaxel in clinical trials, and fatal reactions have occurred in subjects despite premedication.

If a subject has an anaphylactic or severe hypersensitivity reaction to the first dose of paclitaxel (day 1 of cycle 1), he or she will discontinue chemotherapy permanently but may continue to receive AMG 706/placebo monotherapy. Subjects who discontinue all study treatment (AMG 706/placebo, carboplatin, paclitaxel) will be required to complete a safety follow-up visit and will enter the long-term follow-up.

There is no known treatment for paclitaxel overdose. Primary anticipated complications would consist of bone marrow suppression, peripheral neurotoxicity, and mucositis.

6.2.2 Carboplatin

6.2.2.1 Carboplatin Pre-medication

Any pre-medication for carboplatin should be administered at the investigator's discretion.

All premedications must be recorded in the eCRF.

REDACTED

6.2.2.2 Carboplatin Dosage, Administration, and Schedule

Carboplatin to a target AUC of 6 mg/mL x min should be administered IV over approximately 30 ± 10 minutes as soon as possible after the paclitaxel infusion is complete. The investigator will determine frequency of vital sign monitoring during carboplatin infusion.

Carboplatin will be prepared according to the most current package insert.

Carboplatin will be dosed using the creatinine clearance (GFR) and Calvert formula to AUC/time curve of 6 mg/mL x min (see package insert):

Total Dose (mg) = (target AUC) x (GFR + 25)

Cockroft-Gault formula below must be used to estimate creatinine clearance (GFR):

$$\text{Creatinine clearance (GFR)* (mL/min)} = \frac{(140 - \text{age}) \times \text{actual body weight (kg)}}{72 \times \text{serum creatinine (mg/dL)}} \; (\times 0.85 \text{ for females})$$

* The maximum GFR to be used in the Calvert formula is 140 for males and 120 for females.

If body weight changes during therapy by > 5 kg, or there is a significant change in serum creatinine, the carboplatin dose should be re-calculated for the next cycle.

Aluminum reacts with carboplatin causing precipitation and loss of potency. Therefore, needles or IV sets containing aluminum parts that may come in contact with the drug must not be used for the preparation of carboplatin.

6.2.2.3 Carboplatin Safety Profile

The most common adverse events associated with carboplatin are myelosuppresion, nausea, vomiting, and hypomagnesemia. Other less commonly reported adverse events include other gastrointestinal side effects, alopecia, asthenia, peripheral neuropathy, ototoxicity, hypersensitivity reactions, infections, bleeding, and liver function test elevations. Anaphylaxis and severe hypersensitivity reactions have been reported.

There is no known antidote for carboplatin overdose. The anticipated complications of overdose would be secondary to bone marrow suppression and/or hepatic toxicity.

6.2.3 Chemotherapy Toxicity Management

6.2.3.1 Hematological Toxicity

For any delay in chemotherapy due to hematological toxicity, complete blood count and platelet counts must be obtained weekly until they reach the lower limits for treatment. The treatment schedule will then proceed in the usual sequence.

REDACTED

Absolute Neutrophil Count

ANC must be ≥ 1.5 x 10^9/L within 72 hours prior to chemotherapy administration. Treatment must be delayed for up to 3 weeks until ANC is ≥ 1.5 x 10^9/L. If ANC count has not recovered within 3 weeks from planned dose, the subject will discontinue chemotherapy permanently.

If subject experiences febrile neutropenia or ≥ grade 2 infection at any time, granulocyte colony stimulating factor (G-CSF) should be added initially prior to any dose reduction on the next cycle of chemotherapy. For a second episode of febrile neutropenia or ≥ grade 2 infection, paclitaxel and carboplatin doses are reduced to 150 mg/m^2 and AUC of 4.5 mg/mL x min, respectively. A second dose reduction (paclitaxel 100 mg/m^2 and carboplatin AUC of 3.0 mg/mL x min) is permitted if necessary for a third episode of febrile neutropenia or ≥ grade 2 infection. Any dose reductions for neutropenic fever are permanent. If the subject experiences any further episodes of neutropenic fever they will discontinue chemotherapy permanently.

Platelets

Platelet count must be ≥ 100 x 10^9/L within 72 hours prior to chemotherapy administration. Treatment must be delayed for up to 3 weeks until the platelet count is ≥ 100 x 10^9/L. If the platelet count has not recovered within 3 weeks from planned dose, the subject will discontinue chemotherapy permanently.

If platelet count < 25 x 10^9/L at any point during the previous cycle of treatment, paclitaxel and carboplatin will be dose reduced to 150 mg/m^2 and AUC of 4.5 mg/mL x min, respectively. If a second episode is observed, a further dose reduction can be initiated (paclitaxel 100 mg/m^2 and carboplatin AUC of 3.0 mg/mL x min). Any dose reductions are permanent. If the subject experiences any further episodes of thrombocytopenia, the subject will discontinue chemotherapy permanently.

REDACTED

6.2.3.2 Gastrointestinal Toxicity

Nausea and/or Vomiting

If grade 3 or 4 nausea and/or vomiting occur in spite of antiemetics, paclitaxel must be reduced to 150 mg/m^2 and carboplatin reduced to AUC of 4.5 mg/mL x min for the next cycle of treatment. If tolerated the dose can be increased back to baseline as soon as possible.

Stomatitis

If the subject has stomatitis on day 1 of any treatment cycle, chemotherapy treatment must be withheld until the stomatitis is cleared. For grade 3 or 4 stomatitis, paclitaxel must be reduced to 150 mg/m^2 and carboplatin reduced to AUC of 4.5 mg/mL x min until the stomatitis is completely cleared. This dose reduction is permanent.

If there is no recovery after 3 weeks from planned dose, the subject will discontinue chemotherapy permanently.

6.2.3.3 Hepatic Toxicity (Paclitaxel)

Bilirubin and AST

Bilirubin must be < 1.5 x ULN within 3 days prior to chemotherapy administration (except for subjects with UGT1A1 promoter polymorphism, ie, Gilbert syndrome. Subject's enrolled with Gilbert syndrome must have a total bilirubin < 3 x ULN).

The AST/ALT and bilirubin values on day 1 of the cycle will determine the paclitaxel dose:

AST/ALT	Paclitaxel dose modification<br>Bilirubin		
	≤ 1 x ULN	1 to 1.5 x ULN	≥ 1.5 x ULN
≤ 5 x ULN	200 mg/m^2	150 mg/m^2	hold, then 150 mg/m^2
> 5 x ULN	150 mg/m^2	150 mg/m^2	hold, then 150 mg/m^2

Carboplatin must also be held if paclitaxel is held for hepatotoxicity. There will be no dose reductions to the carboplatin for hepatotoxicity.

Treatment with both paclitaxel and carboplatin may be delayed for up to 3 weeks until bilirubin level is < 1.5 x ULN (< 3 x ULN for subjects with UGT1A1 promoter polymorphism).

If there is no recovery after 3 weeks from planned dose, the subject will discontinue chemotherapy permanently.

6.2.3.4 Cardiovascular Toxicity (Paclitaxel)

No treatment is required for asymptomatic bradycardia.

The paclitaxel infusion must be stopped for symptomatic arrhythmias and the arrhythmia must be managed according to standard practice. Chemotherapy will be discontinued permanently.

REDACTED

The infusion must also be stopped if the subject develops any chest pain and/or symptomatic hypotension (< 90/60 mm Hg or requires fluid replacement). An ECG must be performed and IV diphenhydramine and dexamethasone, or other appropriate therapy as per institutional guidelines, must be administered if hypersensitivity is considered. Epinephrine or bronchodilators may be used if chest pain is not considered cardiac. The subject will discontinue chemotherapy permanently.

6.2.3.5 Neurological Toxicity (Paclitaxel)

Any neurological toxicity must recover to grade 1 prior to any further chemotherapy being administered. If any neurological toxicity has not recovered to grade 1 after an additional 3 weeks (6 weeks from planned dose), the subject will discontinue chemotherapy permanently.

If any grade 2 neurological toxicity is observed, paclitaxel will be dose reduced to 150 mg/m^2. If the subject experiences grade 3 or worse neurological toxicity, paclitaxel will be dose reduced to 100 mg/m^2. All dose reductions are permanent.

6.2.3.6 Allergic Reaction/Hypersensitivity (Paclitaxel)

Any subjects who experience severe or life threatening symptoms of hypersensitivity will discontinue chemotherapy permanently.

For moderate symptoms, the paclitaxel infusion must be stopped. IV diphenhydramine 25 to 50 mg, and IV dexamethasone 10 mg, or other appropriate therapy as per institutional guidelines, must be administered. The paclitaxel infusion may be resumed after recovery of symptoms at a low rate (20 mL/hour for 15 minutes, then 40 mL/hour for 15 minutes, then, if no further symptoms, at full dose rate until infusion complete). If symptoms recur the infusion must be stopped.

For severe, life threatening symptoms of hypersensitivity, the paclitaxel infusion must be stopped. IV diphenhydramine 25 to 50 mg, and IV dexamethasone 10 mg, or other appropriate therapy as per institutional guidelines, must be administered. The addition of epinephrine or bronchodilators may be used if indicated.

All hypersensitivity reactions must be recorded as adverse events.

6.2.3.7 Other Toxicity

When a subject experiences any grade 3 or 4 toxicity not already mentioned above, chemotherapy will be withheld until the toxicity resolves to grade 1 (as defined by CTCAE version 3.0) or returns to the subject's baseline.

REDACTED

Subjects will then resume paclitaxel treatment at 100 mg/m^2 and carboplatin to a target AUC of 3.0 mg/mL x min. Dose reductions are permanent.

A subject experiencing grade 3 or 4 toxicity that persists for > 3 weeks or recurs after a dose delay and/or reduction will discontinue chemotherapy permanently.

6.2.4 Multiple Toxicity

If multiple or overlapping toxicity occurs, the most stringent dose modification criteria must be chosen.

6.2.5 Supportive Care for Chemotherapy

Supportive care with approved drugs for chemotherapy-induced side effects is allowed. Treatment with investigational supportive therapy is not allowed.

6.5 Treatment for the Randomized Squamous NSCLC Subjects

Prior to protocol amendment 2, subjects with squamous histology were also randomized. As of 19 November 2008, randomized subjects with squamous NSCLC were discontinued from receiving AMG 706 or placebo treatment. This protocol change was made based on recommendations from the independent Data Monitoring Committee (DMC) that observed a higher rate of hemoptysis in the squamous subpopulation.

Any squamous subjects remaining on study should continue to receive carboplatin and paclitaxel therapy on study, per protocol (up to the maximum of 6 cycles, see Section 6.2).

7. STUDY PROCEDURES

7.5.1 Response Evaluation

Tumor response assessment will be according to modified RECIST criteria (Appendix A) once every 6 ± 1 weeks while on study. Radiological assessment must include CT scan or MRI of the chest, abdomen, pelvis and any other sites of disease.

All the measurements must be performed with the same type of scan throughout the study.

A confirmatory scan will be performed at least 4 weeks after the assessment showing an initial partial (PR) or complete response (CR).

Any progressive disease by bone scan must be confirmed by x-ray, CT or MRI.

Subjects with symptoms suggestive of disease progression must be evaluated for tumor response at the time the symptoms occur.

REDACTED

8. REMOVAL AND REPLACEMENT OF SUBJECTS

8.1 Removal of Subjects

Subjects have the right to withdraw fully or partially from the study at any time and for any reason without prejudice to his or her future medical care by the physician or at the institution.

Withdrawal of full consent for a study means that the subject does not wish to receive further investigational treatment and does not wish to or is unable to continue further study participation. Any subject may withdraw full consent to participate in the study at any time during the study. The investigator will discuss with the subject the most appropriate way to withdraw to ensure the subject's health.

Withdrawal of partial consent means that the subject does not wish to take investigational product any longer but is still willing to collaborate in providing further data by continuing on study. Subjects may decline to continue receiving investigational product at any time during the study. These subjects, as well as those who have stopped receiving investigational product for other reasons (eg, investigator or sponsor concern) will be strongly encouraged to complete the safety follow-up procedures and long-term observation.

If a subject is withdrawn due to an adverse event, the investigator must arrange for the subject to complete a safety follow-up visit at 30 to 33 days after the last administration of AMG 706/placebo or chemotherapy, whichever is administered last. Subjects must be followed until all medically significant related adverse events have resolved or are considered stable.

Reasons for removal from study medication might include:

- Withdrawal of consent
- Administrative decision by the investigator or sponsor
- Pregnancy; (report on Pregnancy Notification Worksheet, see)
- Ineligibility
- Significant protocol deviation
- Subject noncompliance
- Adverse event (report on eCRF)
- Disease progression
- Lost to follow-up

Should a subject (or a legally acceptable representative) request or decide to withdraw from the study, all efforts will be made to complete and report the observations as thoroughly as possible up to the date of withdrawal.

REDACTED

10. STATISTICAL CONSIDERATIONS

10.1 Study Design

This is a phase 3, multicenter, randomized, placebo-controlled, double-blind study comparing the efficacy and tolerability of AMG 706 in combination with paclitaxel and carboplatin to placebo in combination with paclitaxel and carboplatin. Efficacy and tolerability of AMG 706 will be examined for all randomized subjects in the non-squamous full analysis set and by histology subtype (adenocarcinoma and non-squamous, non-adenocarcinoma Section 10.2.5). Subjects with unresectable stage IIIB with pericardial or pleural effusion or stage IV or recurrent non-squamous NSCLC will be randomized in a 1:1 ratio to receive either AMG 706 in combination with paclitaxel and carboplatin (Arm A), or placebo in combination with paclitaxel and carboplatin (Arm B). Randomization will be stratified based on stage (stage IIIB vs. stage IV or recurrent), weight loss (< 5% vs. ≥ 5% in the previous 6 months prior to randomization), sex (male vs. female) and prior adjuvant chemotherapy (yes vs. no).

The primary objective of this study is to determine if treatment with AMG 706 in combination with paclitaxel and carboplatin is superior to treatment with placebo in combination with paclitaxel and carboplatin in subjects with advanced NSCLC based on overall survival for all randomized subjects in the non-squamous full analysis set and in the adenocarcinoma full analysis set. An alpha-split testing strategy (Section 10.6) related to the non-squamous population and the adenocarcinoma subpopulation will be employed to control the type I error rate.

Approximately 1400 subjects (700 subjects per group) will be randomized in this study in a double-blind fashion.

10.2 Study Endpoints, Subsets, and Covariates

10.2.1 Primary Efficacy Endpoint

- Overall survival time: Overall survival time is calculated as the number of days from randomization to death (date of death - date of randomization + 1). Subjects who have not died (no record of death) will be censored at their last contact date.

10.2.2 Secondary Efficacy Endpoints

- Progression-free survival time (PFS): Progression-free survival time is calculated as the number of days from randomization to the date of radiological evidence of disease progression (date of CT or MRI scan, whichever indicates disease progression) or death, regardless of cause (date of progressive disease or death - date of randomization + 1). Subjects who are alive without a disease response assessment of progressive disease will be censored at the last disease assessment date. In the case where there are 2 or more missing tumor assessments followed by

disease progression at the next assessment or death, PFS will be censored at the last complete tumor assessment date prior to the missing assessment. Subjects who withdraw from treatment prior to progression without withdrawing consent will be followed for disease status whenever possible. Subjects who have no response assessments after baseline will be censored on the randomization date. All tumor assessment dates will be based on the actual date of the CT or MRI scans.

- Overall survival time in the PlGF analysis set
- Objective tumor response rate (ORR) (complete [CR] and partial response [PR]) according to modified RECIST criteria in subjects with measurable disease at baseline: The incidence of either a confirmed CR or PR per modified RECIST criteria (responder). Any complete response or partial response will be confirmed with a subsequent CT or MRI scan no less than 4 weeks after the criteria for response are first met
- Duration of response (calculated for only those subjects who respond): The number of days between the date of first tumor response assessment of objective response (including complete response and partial response), which is subsequently confirmed, to the time of the first tumor response assessment of progressive disease or death due to any cause (date of first progressive disease assessment or death - date of first objective response assessment + 1). This will be calculated only for those subjects who have a confirmed objective response based on a review of CT or MRI scans. Subjects who respond and have not progressed while on study will be censored at the date of assessment of the last CT or MRI scan. Subjects who withdraw consent to participate in the study prior to progression will be censored at the date of their last evaluable assessment of response. Subjects who withdraw from treatment prior to progression without withdrawing consent will be followed for disease status whenever possible.
- Pharmacokinetics of AMG 706 (eg, C_{min}, C_{max}) and metabolite concentrations when administered in combination with paclitaxel and carboplatin (in approximately 250 subjects at selected centers).
- Pharmacokinetics (eg, C_{max}) of carboplatin (total and unbound platinum concentrations) when administered in combination with paclitaxel and AMG 706 or placebo (in approximately 20 to 30 subjects at selected centers outside of the European Union).

10.2.3 Safety Endpoints

- Subject incidence of treatment-emergent AEs
- Changes in laboratory values

10.2.4 Exploratory Endpoints

- **Other p**otential biomarker development based on assessment of blood and tumor cells with the proposed mechanism of action of AMG 706 and response
- Effects of genetic variation in drug metabolism genes, cancer genes, and target genes on subject response to AMG 706 in combination with paclitaxel and carboplatin (separate informed consent)
- Patient reported outcomes using EQ-5D and the FACT-L questionnaires

REDACTED

10.2.5 Subsets

The principal analyses for the efficacy endpoints will employ the full analysis set (FAS) approach. PlGF analyses will be performed using the PlGF analysis set. Safety analyses will be performed using the safety analysis set. The non-squamous FAS set is defined as all non-squamous subjects who are randomized. Subjects will be included in the FAS according to their randomized treatment arm assignment. The adenocarcinoma FAS is defined as a subset of the non-squamous FAS and includes only subjects who have adenocarcinoma. The non-squamous PlGF analysis set is defined as all non-squamous subjects who were randomized to the AMG 706 treatment arm, have a value of PlGF recorded at both baseline and week 4, and received AMG 706 the day prior to the week 4 PlGF sample collection. The adenocarcinoma PlGF analysis set is defined as the subjects of the non-squamous PlGF analysis set and includes only subjects who have adenocarcinoma. The non-squamous safety analysis set is defined as all non-squamous subjects who are randomized and receive at least one dose of AMG 706 or placebo. The adenocarcinoma safety analysis set is defined as a subset of the non-squamous safety analysis set and includes only subjects who have adenocarcinoma. Summaries of safety data will also be provided for the complementary non-squamous, non-adenocarcinoma safety analysis set. The summary of safety will also be provided for the PlGF analysis set. Subjects will be included in the safety analyses according to the treatment received.

10.2.6 Covariates

The relationship of the following covariates to overall survival time, objective tumor response rate, and PFS will be explored:

- histology (adenocarcinoma vs non-squamous, non-adenocarcinoma)
- stage of disease at enrollment (stage IIIB vs stage IV or recurrent; recurrent vs. other)
- weight loss in the previous 6 months prior to randomization (< 5% vs. ≥ 5%)
- prior adjuvant chemotherapy (yes vs. no); chemo-naïve (yes vs. no)
- sex (male vs. female)
- region of enrollment (US/Canada/Australia vs. EU vs. rest of world; other categories may be defined if feasible)
- prior radiotherapy (yes vs. no)
- brain metastases (yes vs. no)
- age at study enrollment (< 65 years vs. ≥ 65 years)
- race

REDACTED

- smoking history (≥ 100 cigarettes in a lifetime vs. < 100 cigarettes in a lifetime)

The summaries of treatment emergent AEs will be provided for subgroups defined by age, sex, and race (if feasible).

10.3 Sample Size Considerations

This sample size was based on comparing the treatment groups with respect to the primary efficacy endpoint, overall survival (OS) in the non-squamous population and within the subset of subjects with adenocarcinoma histology. Historical data suggest that a median OS of 10 months is expected for the placebo plus paclitaxel and carboplatin group. An overall 4% significance level will be used with 3.04% allocated to the non-squamous study population and 2.04% allocated to the adenocarcinoma subpopulation, assuming $\sqrt{0.7}$ correlation between the test statistics. Taking into account the alpha spending at the interim, the significance level at the final analysis will be 3% and 2% respectively. Assuming an exponential model for the time to survival, in the non squamous population a total of 742 deaths are required with 80% power to detect a hazard ratio of 1.25 (median OS of 10 months versus 12.5 months) using a 2-sided log-rank test at a 3% significance level ($\alpha = 0.03$). Within the adenocarcinoma subpopulation, a total of 593 deaths are required with 80% power to detect a hazard ratio of 1.3 (median OS of 10 months versus 13 months) using a 2-sided log-rank test at a 2% significance level ($\alpha = 0.02$). With enrollment over 33 months, it is expected that the target number of events will occur at 40 months from the first subject enrolled. A total of 1060 non-squamous subjects (530 subjects/group) will need to be randomized.

It should be noted that a 5% significance level was specified for the overall population (N = 1400) including the squamous subjects. Analysis including squamous subjects is no longer a primary objective and will be descriptive in nature.

The primary efficacy analysis will be performed when approximately 742 non-squamous deaths have occurred (target event goal) and approximately 593 deaths have occurred in the adenocarcinoma subpopulation. The primary analysis will be produced for both the non-squamous full analysis set and the adenocarcinoma full analysis set. Based on the above assumptions, the event goals for both analysis sets are estimated to occur approximately 40 months after the first subject is enrolled in the study. The time to the target events may be adjusted to assure that the required number of events are reached. Amgen will set a single data cut-off date for the primary efficacy analysis in anticipation of 742 non-squamous deaths and 593 deaths within the adenocarcinoma subpopulation based on the later date of reaching the target events. Hence, the efficacy analysis will

REDACTED

be based on the data from randomization through the cut-off date (ie, events occurring after the **cut-off** date are not included in the efficacy analysis). A data cut-off date will also be set for the interim analysis (see Section 10.5).

Pharmacokinetics of AMG 706 and metabolites when administered in combination with paclitaxel and carboplatin will be measured prior to all treatment (predose) on day 1 of cycles 3, 5 and every third visit, and approximately 1 to 2 hours after AMG 706 or placebo administration (postdose) on day 1 of cycle 3 in approximately 250 subjects at selected centers. A sample size of 250 subjects is approximately 20% of the planned enrollment, and will allow for adequate AMG 706 population pharmacokinetic evaluations, if appropriate.

Pharmacokinetics of carboplatin (total and unbound platinum) when administered in combination with AMG 706 and paclitaxel will be measured within 5 minutes after the end of infusion on day 1 of cycles 3 and 5 in approximately 20 to 30 subjects at selected centers outside of the European Union. Approximately 10 subjects will be treated with carboplatin/paclitaxel + placebo and 10 subjects will be treated with carboplatin and paclitaxel in combination with AMG 706. The C_{max} for carboplatin after a single administration had the variability of approximately %CV = 40% in the Amgen 20050191 study with 26% attributable to intra-subject variability.

Table 2 presents the size of the 90% confidence interval for the ratio of the geometric means (with AMG 706 : without AMG 706) based on several point estimates assuming a total subject %CV of 40% with 26% attributable to intra-subject variability. Table 2 assumes that all available PK parameter data are used for estimation including 10 subjects in each treatment arm with at least 1 evaluable C_{max} result (5 subjects in each arm have 2 evaluable C_{max} results).

Table 2. 90% Confidence Intervals on the Ratio of Geometric Means

Point estimate for the ratio	90% CI		
	Lower bound	Upper bound	Width
1	0.81	1.23	0.42
1.05	0.85	1.29	0.44
2	1.63	2.46	0.83

90% CI: average of the lower and upper bounds from 90% confidence intervals calculated from 1,000 simulated studies

10.4 Access to Individual Subject Treatment Assignments

This is a double-blind study. Subjects, investigators, study monitors and the study team will be blinded to the treatment administered. Staff associated with tracking, assaying, and analyzing PK samples may have access to the PK-related information only.

10.5 Interim Analysis and Early Stopping Guidelines

One formal interim analysis of OS will be performed when approximately 370 deaths have occurred in non-squamous population.

At this interim analysis the study may be stopped for overwhelming evidence of efficacy, when efficacy boundaries for both hypotheses are crossed. Adopting a significance level at the interim more conservative than the methods of Lan and DeMets (*Biometrika* 70:659–663, 1983), with O'Brien and Fleming (*Biometrics* 35:549–556, 1979) boundaries, the interim analysis will be conducted at a significance level of 0.0001 (α = 0.0001) for the non-squamous study population and the adenocarcinoma subpopulation respectively. The final analysis for OS will be at the 0.03 and 0.02 levels of significance (2-sided α = 0.03, 0.02) for the respective populations.

The study may also be stopped due to futility at the interim when the futility boundary for either hypothesis is crossed. The futility stopping boundaries are non-binding to allow for additional considerations in situations such as the adenocarcinoma subpopulation is still promising but not the overall non-squamous population.

An independent Data Monitoring Committee (DMC) will review safety and efficacy data at the interim analyses. An independent CRO (Contract Research Organization) will perform these analyses. The DMC will provide a recommendation regarding study continuation based on the safety and efficacy parameters (survival) to the designated Amgen representatives, including the Amgen Oncology Therapeutic Area (OTA) Head. In the event that the study is terminated early based on a DMC recommendation, Amgen will notify the appropriate regulatory authorities. At the interim analysis, if the DMC recommends stopping the study for overwhelming evidence of efficacy or futility, subjects receiving AMG 706 placebo will discontinue placebo tablets and they may be switched to AMG 706 at the recommendation of the DMC or Amgen.

In addition, the DMC will periodically review safety data at regularly scheduled meetings, according to a pre-specified DMC charter. Intensive safety monitoring will be performed in the early portion of the study. Formal safety reviews will occur after approximately 10, 20, and 40 subjects have been randomized to the AMG 706 or placebo arm and

REDACTED

received at least 1 cycle of treatment. In order to preserve the study blind, DMC meetings will occur after 20, 40, and 80 subjects have been randomized in total and receive at least 1 cycle of treatment. The rationale is that the 1:1 randomization ratio used in this study will provide approximately even numbers of subjects from each treatment arm. Subsequently, periodic safety reviews will occur after approximately 100 and 300 subjects have been randomized to the AMG 706 or placebo arm (ie, after 200, and 600 subjects have been randomized in total) and received at least 1 cycle of treatment or if there is agreement between the DMC and Amgen to discontinue or modify the frequency of these review meetings. Accrual to the study will not be interrupted due to the scheduled safety reviews. The DMC or Amgen study team may request an ad hoc meeting for any reason, including significant unexpected safety event, unplanned unblinding of study results, follow-up of an observation during a planned DMC meeting, or a report external to the study, such as publication of study results from a competing product.

At each review, subject incidence rates of adverse events (including all serious, treatment-related, serious treatment-related and events requiring the discontinuation of AMG 706 or placebo) will be tabulated by system organ class, preferred term and severity grade. Listings and/or narratives of “on-study” deaths, deaths within 30 days of receiving AMG 706, paclitaxel, carboplatin or placebo and serious and significant adverse events, including any early withdrawals due to adverse events, will be provided. In addition, an OS Kaplan-Meier (K-M) curve for all subjects in the non-squamous full analysis set and OS K-M curves by histology subtype (adenocarcinoma and non-squamous, non-adenocarcinoma) will be provided.

Records of all meetings will be archived. The DMC will communicate major safety concerns and recommendations regarding study modification or termination to Amgen.

Further details will be provided in the DMC charter.

10.6 Planned Methods of Analysis

10.6.1 General Approach/Considerations

The primary analyses will be conducted based on the non-squamous full analysis set (FAS), consisting of all randomized non-squamous subjects and by histology subtype (adenocarcinoma FAS and non-squamous, non-adenocarcinoma FAS). Safety analyses will be performed using both the non-squamous safety analysis set and both the adenocarcinoma and non-squamous, non-adenocarcinoma safety analysis sets.

REDACTED

The final analysis for treatment effect will be performed at a significance level of 3% in the non-squamous population and 2% in the adenocarcinoma subpopulation. All tests will be 2-sided unless otherwise indicated. The interim analysis of OS will be conducted at a significance level of 0.0001 (α = 0.0001) as described in Section 10.5. The primary analyses will be performed based on a data cut-off date for the primary efficacy analysis in anticipation of 742 non-squamous deaths overall and 593 deaths within the adenocarcinoma subpopulation. Hence, the efficacy analysis will be based on the data from randomization through the data cut-off date (ie, events occurring after the data cut-off date are not included in the efficacy analysis). A data cut-off date will also be set for the interim analysis.

A fixed sequence testing procedure will be followed for the key secondary efficacy endpoints. PFS endpoint will be tested following significance of OS, PIGF related biomarker hypotheses will be tested following significance on PFS, and finally the objective response rate will be tested following significance of PIGF related biomarker hypotheses.

The same alpha split levels will be used for non-squamous (3%) and for adenocarcinoma (2%) subpopulation when testing the secondary endpoints. No alpha reallocation is permitted should the testing on the non-squamous population or the adenocarcinoma subpopulation be stopped.

10.6.2 Analysis of Key Study Endpoints

The primary analyses will be conducted on the non-squamous full analysis set, consisting of all randomized non-squamous subjects, and on the subset of subjects with adenocarcinoma histology. Subjects will be included in the analysis according to their randomized treatment arm assignment. An overall 4% significance level will be used with 3% level for the non-squamous study population and 2% level for the adenocarcinoma subpopulation at the final analysis, assuming $\sqrt{0.7}$ correlation between the test statistics. It is estimated that adenocarcinoma is approximately 74% of non-squamous NSCLC. As a conservative approach, as long as the lower limit of the 95% confidence interval of the proportion of adenocarcinoma subjects is at least 70%, the overall type 1 error rate for the non-squamous study population is controlled to not exceed 4%. If the final estimator of the lower limit of the 95% confidence interval is less than 70%, the actual lower limit will be used to allocate alpha for the adenocarcinoma subpopulation.

REDACTED

The primary efficacy analysis will be performed when approximately 742 deaths have occurred in the non-squamous population and 593 deaths have occurred in the adenocarcinoma subpopulation. This is estimated to occur approximately 38 months after the first subject is enrolled in the study. An updated survival analysis will be performed at the end of the study. Following study unblinding and review of the primary analysis, subjects receiving AMG 706 placebo will discontinue placebo tablets and may be switched to AMG 706 at the discretion of Amgen.

For the non-squamous FAS, stratified log-rank test (using the randomization stratification factors of stage (stage IIIB vs. stage IV or recurrent), weight loss (<5% vs. ≥ 5 % in the previous six months prior to randomization), sex (male vs. female), and prior adjuvant chemotherapy (yes vs. no)) will be used to compare the treatment arms with respect to OS with the null hypothesis that there is no difference between the treatment arms versus a 2-sided alternative at 3% significance level. In addition, the stratified Cox model will also be used to estimate the hazard ratio for the treatment effect with respect to OS (using the stratification factors of stage (stage IIIB vs. stage IV or recurrent), weight loss (<5% vs. ≥ 5 % in the previous six months prior to randomization), sex (male vs. female), and prior adjuvant chemotherapy (yes vs. no)) and to produce a 95% confidence interval for the hazard ratio. For each treatment arm, the Kaplan-Meier (K-M) survival curves will be presented. The K-M medians (if estimable) will also be derived, along with their 2-sided 95% confidence intervals. As a sensitivity analysis, the weighted test statistic Z_N will be calculated. It is a weighted average of the test statistics for adenocarcinoma subpopulation (Z_M) and non-squamous, non-adenocarcinoma subpopulation (Z_{N-M}) based on the stratified log-rank test in the respective subpopulation (using the randomization stratification factors of stage (stage IIIB vs. stage IV or recurrent), weight loss (<5% vs. ≥ 5 % in the previous six months prior to randomization), sex (male vs. female), and prior adjuvant chemotherapy (yes vs. no)). The weights are based on the proportion of subjects in the targeted subpopulation,

ie, $Z_N = \sqrt{p} * Z_M + \sqrt{(1-p)} * Z_{N-M}$, where p is the proportion of adenocarcinoma subjects.

For the adenocarcinoma FAS, stratified log-rank test (using the randomization stratification factors of stage (stage IIIB vs. stage IV or recurrent), weight loss (<5% vs. ≥ 5 % in the previous six months prior to randomization), sex (male vs. female), and prior adjuvant chemotherapy (yes vs. no)) will be used to compare the treatment arms with respect to OS with the null hypothesis that there is no difference between the treatment arms versus a 2-sided alternative at 2% significance level. In addition, the stratified Cox

REDACTED

model will also be used to estimate the hazard ratio for the treatment effect with respect to OS (using the stratification factors of stage (stage IIIB vs. stage IV or recurrent), weight loss (<5% vs. ≥ 5 % in the previous six months prior to randomization), sex (male vs. female), and prior adjuvant chemotherapy (yes vs. no)) and to produce a 95% confidence interval for the hazard ratio. For each treatment arm, the Kaplan-Meier (K-M) survival curves will be presented. The K-M medians (if estimable) will also be derived, along with their 2-sided 95% confidence intervals.

Fixed sequence testing procedure will be followed for the secondary efficacy endpoints. Separate testing procedures will be employed for the non-squamous population and the adenocarcinoma subpopulation. For the non-squamous population, if the primary endpoint of OS is significant at the 0.03 level of significance, PFS will be tested at the 0.03 level of significance. If PFS is significant at the 0.03 level of significance, each of the PlGF related biomarker hypotheses will be tested at the 0.03 level of significance sequentially. If both PlGF related biomarker hypotheses are significant at the 0.03 level, ORR will be tested at the 0.03 level of significance. The same testing sequence will be taken for the adenocarcinoma subpopulation with a 0.02 significance level if OS is significant at the 0.02 level.

The same methodology described for OS will be applied to PFS. The stratified log-rank tests will be used to compare the treatment arms for PFS for the non-squamous population and for the adenocarcinoma subpopulation, respectively. In addition stratified Cox model will be used to estimate the hazard ratio for treatment effect for the non-squamous population and the adenocarcinoma subpopulation, respectively. The associated 95% confidence interval for PFS for each population of interest will also be provided. The Kaplan-Meier (K-M) curves will be presented for each treatment group. The K-M medians (if estimable) will also be derived, along with their 2-sided 95% confidence intervals.

The assessment of OS in the PlGF analysis set will first be evaluated using a Cox model with the log-transformed PlGF fold change between baseline and week 4 as a continuous variable with no other covariates in the model. The hazard ratio associated with each unit increase of log-transformed PlGF fold change will be provided with corresponding 95% confidence interval. If PlGF fold change as a continuous variable is shown to be significant at the respective significance level, the log-transformed PlGF fold change between baseline and week 4 will be assessed as a binary variable (PlGF high vs. PlGF low) using an unadjusted logrank test. The hazard ratio and the 95%

REDACTED

confidence interval for the PlGF status (PlGF high vs. PlGF low) K-M curves will be presented for OS with the estimates for OS and the associated 95% CIs in the rates at selected time points for each PlGF status. The data from the PlGF undetermined subjects will also be described.

The number and percentage of subjects with measurable disease within each category of response (CR, PR, SD, PD, non-evaluable, and missing) will be summarized. The percentage will be calculated by dividing the number of subjects within each category of response by the number of subjects with measurable disease. Each subject will be counted within only one response group, with the best response during the study as the classification group. The treatment arms will be compared with respect to objective response rate (PR and CR) using the stratified Cochran-Mantel-Haenszel test with the stratification factors as covariates based on the subjects with measurable disease for the non-squamous population (at 3% significance level as needed) and for the adenocarcinoma subpopulation (at 2% significance level as needed). Two-sided 95% confidence interval will be calculated for the differences in objective response rates between the 2 treatment arms using the normal approximation to the binomial distribution. Descriptive statistics will be provided for best overall tumor response for each treatment arm based on the subjects with measurable disease. The number and proportion of subjects with measurable disease within each category of response (CR, PR, SD, PD, and missing) will be presented. The number of subjects with non-measurable disease at baseline will be summarized for each treatment group.

For duration of response, Kaplan-Meier (K-M) curves will be presented for each treatment group and for each population of interest. No formal statistical testing will be performed to compare the treatment groups on this endpoint.

For the non-squamous, non-adenocarcinoma subpopulation, the same analyses will be provided for all the endpoints described above for descriptive purposes.

REDACTED

10.7 Additional Analyses

10.7.1 Analysis of Safety Endpoints

Study data will be monitored on an ongoing basis by the clinical study team to ensure subjects' safety. Additional safety reviews will be performed by the DMC periodically throughout the study. These reviews will include all available data on incidence of adverse events, serious adverse events including deaths, and events leading to withdrawal. The safety data review by Amgen's study team and the Global Safety

Organization will be blinded. Any unblinding of SAEs by Global Safety due to regulatory requirements will be shared with the DMC.

All reported adverse events (AEs) will be assigned system organ class, and preferred term, according to the Medical Dictionary for Regulatory Activities (MedDRA).

Safety data will be summarized for the non-squamous population safety analysis set and separately for the adenocarcinoma and non-squamous, non-adenocarcinoma safety analysis sets. AEs will be considered to be treatment emergent if the event occurs on or after the first administration of protocol specified treatment. The subject incidence rates of adverse events will be tabulated by system organ class, preferred term and severity grade for all treatment-emergent, serious, treatment-related, and serious treatment-related adverse events. Each of these outputs will include tabulation by maximum severity for each system organ class and preferred term as reported by the investigator based on Common Terminology Criteria for Adverse Events (CTCAE), version 3.0. Summary tables will be provided separately for adverse events leading to investigational product discontinuation and adverse events leading to study withdrawal. All “on-study” deaths, ie, deaths that occur within 30 days after last dose of study treatment (AMG 706, AMG 706 placebo or chemotherapy, whichever is administered last), will be summarized. Listings and/or narratives of “on-study” deaths, serious and significant AEs, including early withdrawals due to AEs, also will be provided.

Laboratory parameters for hematology and serum blood chemistry will be summarized at baseline and last observed value. Additionally, the maximum and minimum observed post-baseline values will be summarized along with the change from baseline to the maximum observed value, minimum observed value and last observed value. Tables of shifts in severity (by CTCAE version 3.0) from baseline for selected laboratory parameters and selected time points may also be provided. Graphical representations of aggregate data may also be presented for parameters of interests.

The summaries of the subject incidence rates of treatment-emergent AEs by system organ class and preferred term reported AEs will also be provided for subgroups defined by age, sex, and race (if feasible).

The summary of safety will also be provided for the PlGF analysis set displayed by PlGF low and high.

REDACTED

10.7.2 Analysis of Patient Reported Outcomes (PRO)

PRO measures include the EQ-5D and FACT-L. Subjects will complete the EQ-5D and FACT-L questionnaires on day 1 of cycles 1, 3 and 6 and every third visit thereafter if continuing to receive AMG 706 or placebo and a final assessment will be completed at the safety follow-up visit.

The outcomes from the EQ-5D to be analyzed are the health state preference (also known as the visual analog scale [VAS]) and utility score (health state index). The outcomes from the FACT-L are the 4 subscales (physical well-being [PWB], social/family well-being [SWB], emotional well-being [EWB] and functional well-being [FWB]), the lung cancer subscale (LCS), the FACT-L total score and the TOI (sum of the physical well-being, functional well-being and the LCS scores).

Descriptive summaries on the observed data will be provided for the FACT-L outcomes (total score, 4 subscales, LCS subscale and TOI) and the EQ-5D (health state preference and utility score) at each assessed time point. Missing PRO data and reason for missing data will be reported.

The AUC will be computed for each subject for FACT-L outcomes (total score, 4 subscales, LCS subscale and TOI).

The treatment arms will be compared with respect to the FACT-L outcomes (total score, 4 subscales, LCS and TOI) using analysis of covariance ANCOVA models, controlling for the baseline PRO score and stratification variables. Two-sided 95% confidence intervals will be calculated for the differences between the 2 treatment arms. When assessing symptom changes, data on concomitant interventions affecting these outcomes will also be controlled. Longitudinal models will also be examined for treatment comparisons on the FACT-L outcomes.

The minimal clinically meaningful change on the LCS is a 2 to 3 point difference, while a 5 to 6 point change in the TOI score reflects a clinically meaningful change.

Summaries will be generated for the non-squamous population and separately for the adenocarcinoma and non-squamous, non-adenocarcinoma subpopulations.

10.7.3 Analysis of Pharmacokinetic Data (AMG 706 and Metabolites)

AMG 706 C_{min} values (predose drawn on day 1 of cycles 3 and 5 and every third visit) and C_{max} value (post dose drawn approximately 1 to 2 hours after AMG 706 on day 1 **of** cycle 3) from approximately 125 subjects will be obtained to evaluate intra-subject variability. The corresponding metabolite concentrations from the same time points will

REDACTED

be evaluated. Descriptive statistics of the individual AMG 706 and metabolites concentration values will be provided.

If deemed appropriate, additional analyses such as descriptive statistics of the individual AMG 706 and metabolites values by tumor response and population pharmacokinetic evaluations may be performed.

10.7.4 Analysis of Pharmacokinetic Data (Carboplatin C_{max})

Carboplatin (total and unbound platinum) C_{max} values (end of infusion drawn on day 1 of cycles 3 and 5) from approximately 20 to 30 subjects will be obtained. Descriptive statistics of the individual carboplatin C_{max} values on day 1 of cycles 3 and 5 will be provided by treatment received (AMG 706 or placebo). The point estimate and 90 percent confidence intervals for the ratio of carboplatin C_{max} between the treatment groups will be summarized.

If deemed appropriate, additional analyses of the effect of carboplatin C_{max} on tumor response and population evaluations may be performed.

10.7.5 Analysis of Pharmacogenetic and Biomarker Data

All statistical analysis on biomarkers will be considered exploratory, except for PlGF. Summary statistics will be provided as appropriate and the relationship between parameters will be assessed. The difference in biomarker results from samples taken before, during and after AMG 706 treatment will be investigated.

Summaries may be generated for the non-squamous population and separately for the adenocarcinoma and non-squamous, non-adenocarcinoma subpopulation.

10.7.6 Analysis of the Squamous Cell histology Data

Analysis for the squamous cell subjects will be descriptive in nature and will include summaries of safety, overall survival, progression-free survival, objective response (only in subjects with measurable disease), and duration of response.

REDACTED

Appendix A. Modified RECIST Criteria

Quick References

Eligibility

Only patients with measurable disease at baseline should be included in protocols where objective tumor response is the primary endpoint.

Measurable disease

Measurable disease is defined as the presence of at least one measurable lesion. If the measurable disease is restricted to a solitary lesion, its neoplastic nature should be confirmed by cytology/histology.

Measurable lesions

Lesions that can be accurately measured in at least one dimension with longest diameter ≥ 20 mm using conventional techniques or ≥10 mm with spiral CT scan.

Non-measurable lesions

All other lesions, including small lesions (longest diameter < 20 mm with conventional techniques or < 10 mm with spiral CT scan), ie, bone lesions, leptomeningeal disease, ascites, pleural/pericardial effusion, inflammatory breast disease, lymphangitis cutis/pulmonis, cystic lesions, and also abdominal masses that are not confirmed and followed by imaging techniques.

All measurements should be taken and recorded in metric notation, using a ruler or calipers. All baseline evaluations should be performed as closely as possible to the beginning of treatment and never more than 4 weeks before the beginning of the treatment.

The same method of assessment and the same technique should be used to characterize each identified and reported lesion at baseline and during follow-up.

Clinical lesions will only be considered measurable when they are superficial (eg, skin nodules and palpable lymph nodes). For the case of skin lesions, documentation by color photography, including a ruler to estimate the size of the lesion, is recommended.

Methods of Measurement

CT and MRI are the best currently available and reproducible methods to measure target lesions selected for response assessment. Conventional CT and MRI should be performed with cuts of 10 mm or less in slice thickness contiguously. Spiral CT should be performed using a 5 mm contiguous reconstruction algorithm. This applies to tumors of the chest, abdomen and pelvis. Head and neck tumors and those of extremities usually require specific protocols.

Lesions on chest X-ray are acceptable as measurable lesions when they are clearly defined and surrounded by aerated lung. However, CT is preferable.

When the primary endpoint of the study is objective response evaluation, ultrasound (US) should not be used to measure tumor lesions. It is, however, a possible alternative to clinical measurements of superficial palpable lymph nodes, subcutaneous lesions and thyroid nodules. US might also be useful to confirm the complete disappearance of superficial lesions usually assessed by clinical examination.

The utilization of endoscopy and laparoscopy for objective tumor evaluation has not yet been fully and widely validated. Their uses in this specific context require sophisticated equipment and a high level of expertise that may only be available in some centers. Therefore, the utilization of such techniques for objective tumor response should be restricted to validation purposes in specialized centers. However, such techniques can be useful in confirming complete pathological response when biopsies are obtained.

REDACTED

Tumor markers alone cannot be used to assess response. If markers are initially above the upper normal limit, they must normalize for a patient to be considered in complete clinical response when all lesions have disappeared.

Cytology and histology can be used to differentiate between PR and CR in rare cases (eg, after treatment to differentiate between residual benign lesions and residual malignant lesions in tumor types such as germ cell tumors).

Baseline documentation of “Target” and “Non-Target” lesions

All measurable lesions up to a maximum of 5 lesions per organ and 10 lesions in total, representative of all involved organs should be identified as *target lesions* and recorded and measured at baseline.

Target lesions should be selected on the basis of their size (lesions with the longest diameter) and their suitability for accurate repeated measurements (either by imaging techniques or clinically).

A sum of the longest diameter (LD) for *all target lesions* will be calculated and reported as the baseline sum LD. The baseline sum LD will be used as reference by which to characterize the objective tumor.

All other lesions (or sites of disease) should be identified as *non-target lesions* and should also be recorded at baseline. Measurements of these lesions are not required, but the presence or absence of each should be noted throughout follow-up.

Response Criteria Evaluation of target lesions

* Complete Response (CR):	Disappearance of all target lesions
* Partial Response (PR):	At least a 30% decrease in the sum of the LD of target lesions, taking as reference the baseline sum LD
* Progressive Disease (PD):	At least a 20% increase in the sum of the LD of target lesions, taking as reference the smallest sum LD recorded since the treatment started or the appearance of one or more new lesions
* Stable Disease (SD):	Neither sufficient shrinkage to qualify for PR nor sufficient increase to qualify for PD, taking as reference the smallest sum LD since the treatment started

Evaluation of non-target lesions

* Complete Response (CR):	Disappearance of all non-target lesions and normalization of tumor marker level
* Incomplete Response/ Stable Disease (SD):	Persistence of one or more non-target lesion(s) or/and maintenance of tumor marker level above the normal limits
* Progressive Disease (PD):	Appearance of one or more new lesions and/or unequivocal progression of existing non-target lesions (1)

(1) Although a clear progression of “non target” lesions only is exceptional, in such circumstances, the opinion of the treating physician should prevail and the progression status should be confirmed later on by the review panel (or study chair).

REDACTED

Evaluation of best overall response

The best overall response is the best response recorded from the start of the treatment until disease progression/recurrence (taking as reference for PD the smallest measurements recorded since the treatment started). In general, the patient's best response assignment will depend on the achievement of both measurement and confirmation criteria.

Target lesions	Non-Target lesions	New Lesions	Overall response
CR	CR	No	CR
CR	Incomplete response/SD	No	PR
PR	Non-PD	No	PR
SD	Non-PD	No	SD
PD	Any	Yes or No	PD
Any	PD	Yes or No	PD
Any	Any	Yes	PD

Patients with a global deterioration of health status requiring discontinuation of treatment without objective evidence of disease progression at that time should be classified as having "symptomatic deterioration". Every effort should be made to document the objective progression even after discontinuation of treatment.

In some circumstances it may be difficult to distinguish residual disease from normal tissue. When the evaluation of complete response depends on this determination, it is recommended that the residual lesion be investigated (fine needle aspirate/biopsy) to confirm the complete response status.

Confirmation

The main goal of confirmation of objective response is to avoid overestimating the response rate observed. In cases where confirmation of response is not feasible, it should be made clear when reporting the outcome of such studies that the responses are not confirmed.

To be assigned a status of PR or CR, changes in tumor measurements must be confirmed by repeat assessments that should be performed no less than 4 weeks after the criteria for response are first met. Longer intervals as determined by the study protocol may also be appropriate.

In the case of SD, follow-up measurements must have met the SD criteria at least once after study entry at a minimum interval (in general, not less than 6-8 weeks) that is defined in the study protocol

Duration of overall response

The duration of overall response is measured from the time measurement criteria are met for CR or PR (whichever status is recorded first) until the first date that recurrence or PD is objectively documented, taking as reference for PD the smallest measurements recorded since the treatment started.

REDACTED

Duration of stable disease

SD is measured from the start of the treatment until the criteria for disease progression are met, taking as reference the smallest measurements recorded since the treatment started.

The clinical relevance of the duration of SD varies for different tumor types and grades. Therefore, it is highly recommended that the protocol specify the minimal time interval required between 2 measurements for determination of SD. This time interval should take into account the expected clinical benefit that such a status may bring to the population under study.

REDACTED
